# A pan-cancer analysis of the role of hexokinase II (HK2) in human tumors

**DOI:** 10.1038/s41598-022-23598-8

**Published:** 2022-11-05

**Authors:** Ruiqi Li, Shuchong Mei, Qiang Ding, Qingming Wang, Li Yu, Fuming Zi

**Affiliations:** 1 Department of Hematology, The Second Affiliated Hospital of Nanchang University, No.1 Minde Road, Donghu District, Nanchang, 330006 Jiangxi People’s Republic of China; 2 Institute of Hematology, Nanchang University, Nanchang, China; 3 Key Laboratory of Hematology, Nanchang, 330006 Jiangxi People’s Republic of China

**Keywords:** Oncogenes, Databases

## Abstract

More and more evidence show that HK2 is closely related to tumors. But no pan-cancer analysis is available. This paper aimed to explore the potential roles of HK2 across thirty-three tumors based on the datasets of the cancer genome Atlas (TCGA) and gene expression omnibus. *HK2* is highly expressed in most tumors and related to the progression of some tumors. *HK2* expression was associated with the infiltration of T follicular helper cells for the TCGA tumors of uveal melanoma, breast invasive carcinoma (BRCA), breast invasive carcinoma-luminalA (BRCA-LumA), head and neck squamous cell carcinoma (HNSC), head and neck squamous cell carcinoma with HPV positive (HNSC-HPV^+^), and cancer-associated fibroblasts for the tumors of brain lower grade glioma and stomach adenocarcinoma. Our first pan-cancer study offers a relatively comprehensive understanding of the roles of HK2 in different tumors.

## Introduction

Considering the complexity of tumorigenesis and the different functions of the same gene in different tumors, it is very important to carry out the analysis of pan-cancer expression, evaluate its role in different tumors and its correlation with clinical prognosis, as well as the potential molecular mechanism of the gene. It is helpful for researchers in different fields to better grasp the role of this gene in tumor as a whole. TCGA database and GEO database contain functional genomic datasets of different tumors, which can help us with pan-cancer analysis^1,2^.

One hallmark of tumors is metabolic reprogramming, i.e., the “Warburg effect” is their propensity to metabolize glucose to lactic acid even in the presence of oxygen^3^. The pivotal player in this frequent cancer phenotype is mitochondrial-bound hexokinase. Hexokinase 2 (HK2) is the major bound hexokinase isoform expressed in cancers that exhibit a “Warburg effect”^4^. It plays a central role in the cellular uptake and utilization of glucose and is highly expressed in a variety of cancer cells, but only in a few normal adult tissues (skeletal muscle, heart, adipose tissue)^4–6^. HK2 helps immortalize cancer cells and escapes chemotherapy inhibition. With the reemergence and acceptance of the “Warburg effect” as a prominent phenotype of clinical cancers, metabolic targeting as a therapeutic strategy is rational^3,4,6^.

In this study, we used TCGA and GEO databases to conduct pan-cancer analysis of HK2, including the expression of the *HK2* gene in different tumors, the effect of HK2 on tumor survival, immune-infiltrating cells and related cellular pathway changes, etc., to explore the potential molecular mechanism of HK2 in the pathogenesis or clinical prognosis of different tumors.

## Materials and methods

### Gene expression and analysis

We enter HK2 in the “Gene_DE” module of TIMER2 (tumor immune estimation resource, version 2) website (http://timer.cistrome.org/) and observed the expression of HK2 between tumor and normal tissues of the different tumors of the TCGA project. For tumors without normal control tissues, we used the “Expression analysis-Box Plot” module of the GEPIA2 (Gene Expression Profiling Interactive Analysis, version 2) database (http://gepia2.cancer-pku.cn/#analysis) as a supplement^7^ to obtain the expression of tumor tissues and corresponding normal tissues of the GTEx (Genotype-Tissue Expression) database, under the settings of *P*-value cutoff = 0.01, log_2_FC (fold change) cutoff = 1, and “Match TCGA normal and GTEx data”. Furthermore, we got violin plots of the HK2 expression in different pathological stages (stage I, II, III, and IV) of all tumors via the “Expression analysis-Stage Plot” module of GEPIA2.

We used the UALCAN portal (http://ualcan.path.uab.edu/analysis-prot.html) to conduct protein expression analysis with the CPTAC (Clinical proteomic tumor analysis consortium) module^8^.

Human Protein Atlas (HPA) is a database that focused on exploring the human protein in cells, tissues, and organs based on the combination of several omics technologies^9^. In this study, the protein expression level of breast cancer, renal cancer, colon cancer, lung cancer, ovarian cancer, and endometrial cancer were obtained from the tumor tissues and corresponding normal tissues of the HPA dataset.

### Survival prognosis analysis

The “Survival Analysis-Survival Map” module of GEPIA2 was used to obtain the overall survival (OS) and disease-free survival (DFS) data of HK2 in all TCGA tumors^7^^.^ We used cutoff-high (50%) and cutoff-low (50%) values as the expression thresholds for defining the high-expression and low-expression cohorts. The log-rank test was used in the hypothesis test, and the survival plots were also obtained through the “Survival Analysis” module of GEPIA2.

### Genetic alteration analysis

For the genetic alteration characteristics of HK2, we used the cBioPortal web (https://www.cbioportal.org/)^10^ and chose the “TCGA PanCancer Atlas Studies-Query By Gene” in the “Quick select” section and entered “HK2”. The alteration frequency, mutation type and CNA (Copy number alteration) of HK2 of all TCGA tumors were observed in the “Cancer Types Summary” module. The mutated site of HK2 was displayed in the schematic diagram of the protein structure or the 3D (Three-dimensional) structure via the “Mutations” module. The overall survival, disease-free survival, progression-free survival, and disease-free survival were obtained from the “Comparison” module of the TCGA cancer cases with or without HK2 genetic alteration. Kaplan–Meier plots with log-rank P-value were generated as well.

### Immune infiltration analysis

The “Immune-Gene” module of the TIMER2 web was used to explore the association between HK2 expression and immune infiltration of all TCGA tumors. The immune cells of cancer-associated fibroblasts (CAFs) and T follicular helper cells (Tfh) were selected. The EPIC, MCPCOUNTER, XCELL, TIDE, CIBERSORT, and CIBERSORT-ABS algorithms were applied for immune infiltration estimations. The *P*-values and partial correlation (cor) values were acquired via the purity-adjusted Spearman’s rank correlation test. The data were demonstrated as a heatmap and a scatter plot.

### HK-2-related gene enrichment analysis

Protein name (“HK2”) and organism (“Homo sapiens”) were entered in the STRING website (https://string-db.org/) for protein–protein interaction. Subsequently, we set the following main parameters: meaning of network edges (“evidence”), active interaction sources (“experiments”), minimum required interaction score [“Low confidence (0.150)”], and max number of interactors to show (“no more than 50 interactors” in 1st shell). Then, the determined HK2-binding proteins were available.

To obtain the top 100 HK2-correlated targeting genes based on the datasets of all TCGA tumors and normal tissues, the “Expression Analysis-Similar Gene Detection” module of GEPIA2 was used. We also used the “Expression Analysis-Correlation Analysis” module of GEPIA2 to perform a coupled gene Pearson correlation analysis of HK2 and selected genes. The log2 TPM was applied for the dot plot. Moreover, we used the “Exploration-Gene_Corr” module of TIMER2 to obtain the heatmap data of the selected genes.

We used the Jvenn website (http://bioinformatics.psb.ugent.be/webtools/Venn/) to conduct an intersection analysis to compare the top 100 HK2-binding genes and interacted with above ≤ 50 genes^11^. Then, the two parts of data were combined to perform KEGG (Kyoto encyclopedia of genes and genomes) pathway analysis^12^. The above total gene list was uploaded to the DAVID (Database for annotation, visualization, and integrated discovery) website (https://david.ncifcrf.gov/) with the settings of selected identifier (“OFFICIAL_GENE_SYMBOL”) and species (“Homo sapiens”). Moreover, we analyzed the above gene list with the “Functional Annotation Chart” module of DAVID and chose only the KEGG pathway. The file was downloaded and data of *P* value ≤ 0.05 were selected. The enriched pathways were finally visualized with the Bioinformatics website (http://www.bioinformatics.com.cn).

## Results

### Gene expression analysis data

**Figure 1:**
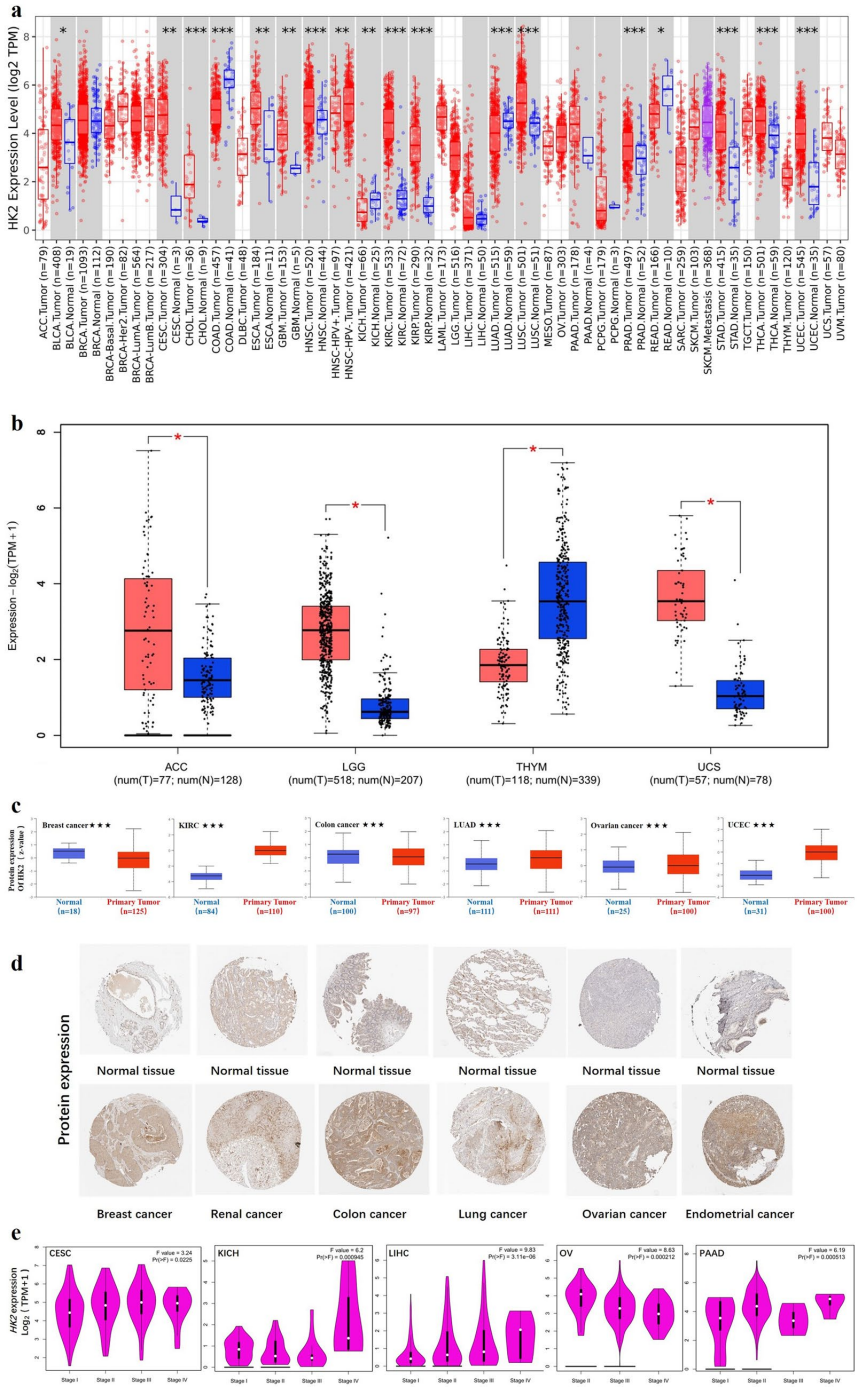
The expression level of *HK2* gene in different tumors and pathological stages. (**a**) The expression status of the *HK2* gene in different cancers or specific cancer subtypes was analyzed through TIMER2. * *P* < 0.05; ** *P* < 0.01; *** *P* < 0.001. (**b**) For the type of ACC, LGG, THYM, and UCS in the TCGA project, the corresponding normal tissues of the GTEx database were included as controls. The box plot data were supplied. ** *P* < 0.01. (**c**) The expression level of HK2 total protein based on the CPTAC dataset between normal tissue and primary tissue of breast cancer, KIRC, colon cancer, LUAD, ovarian cancer, and UCEC. *** *P* < 0.001. (**d**) The expression level of HK2 protein based on the HPA database between normal tissue and primary tissue of breast cancer, renal cancer, colon cancer, lung cancer, ovarian cancer, and endometrial cancer. (**e**) Based on the TCGA data, the expression levels of the *HK2* gene were analyzed by the main pathological stages (stage I, stage II, stage III, and stage IV) of CESC, KICH, LIHC, ovarian cancer, and PAAD. Log2 (TPM + 1) was applied for the log-scale.

In this study, we aimed to explore the role of human HK2 (HM_000189.5 for mRNA or NP_000180.2 for protein) in tumors. We first analyzed the expression of HK2 in different tumors and nontumor tissues. The TIMER2 database was used to analyze the expression of *HK2* in various cancers of TCGA. As shown in Fig. 1a, the expression of *HK2* in the tumor tissues of CHOL (Cholangiocarcinoma), HNSC (Head and neck squamous cell carcinoma), KIRC (Kidney renal clear cell carcinoma), KIRP (Kidney renal papillary cell carcinoma), LUSC (Lung squamous cell carcinoma), PRAD (Prostate adenocarcinoma), STAD (Stomach adenocarcinoma), THCA (Thyroid carcinoma), UCEC (Uterine corpus endometrial carcinoma) (*p* < *0.001*), CESC (Cervical squamous cell carcinoma and endocervical adenocarcinoma), ESCA (Esophageal carcinoma), GBM (Glioblastoma multiforme) (*p* < *0.01*), and BLCA (Bladder urothelial carcinoma) (*p* < *0.05*) is higher than the corresponding normal tissues.

We further evaluated the expression of HK2 between the normal tissues and tumor tissues of ACC (Adrenocortical carcinoma), LGG (Brain lower grade glioma), THYM (Thymoma), and UCS (Uterine carcinosarcoma) (Fig. 1b, p < *0.01*) with GTEx dataset as a complement of TIMER2 database. However, there was no significant difference for other tumors, such as BRCA (Breast invasive carcinoma), LIHC (Liver hepatocellular carcinoma), PAAD (Pancreatic adenocarcinoma), PCPG (Pheochromocytoma and paraganglioma) as shown in Fig. 1a.

The results of the CPTAC datasets showed higher expression of HK2 total protein in the primary tissue of breast cancer, KIRC, colon cancer, LUAD (Lung adenocarcinoma), ovarian cancer and UCEC (Fig. 1c, *p* < *0.001)* than in normal tissues. The above results were further verified by HPA database as demonstrated in Fig. 1d.

The “Pathological Stage Plot” module of GEPIA2 was used to analyze the correlation between *HK2* expression and the pathological stages of cancers, including CESC, KICH (Kidney Chromophobe), LIHC, ovarian cancer and PAAD (Fig. 1d, all *p* < *0.05*) but not others.

### Survival analysis data

**Figure 2:**
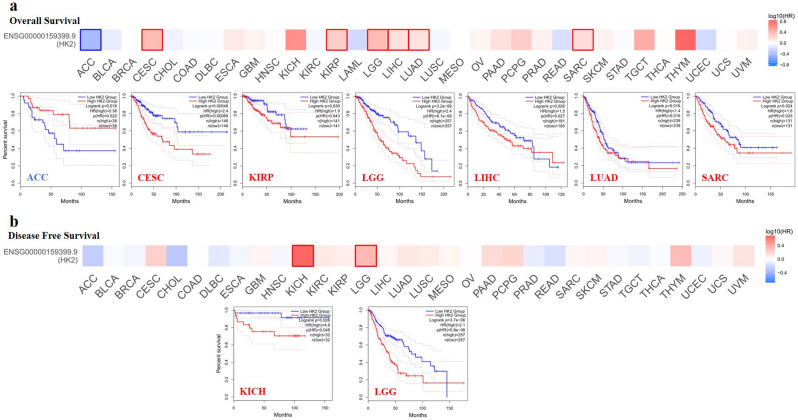
Correlation between *HK2* gene expression and survival prognosis of cancers in TCGA. The GEPIA2 was used to perform overall survival (**a**) and disease-free survival (**b**) analyses of different tumors in TCGA by *HK2* gene expression. The survival map and Kaplan–Meier curves with positive results are given.

According to the expression levels of *HK2*, we divided the cancer cases into high-expression and lower-expression groups and investigated the correlation of *HK2* expression with the prognosis of patients with different tumors, mainly using the datasets of TCGA and GEO, respectively. As shown in Fig. 2a and b, highly expressed *HK2* was linked to poor prognosis of overall survival (OS) for cancers of CESC (*p* = 0.00069), KIRP (*p* = 0.043), LGG (*p* = 0.0000061), LIHC (*p* = 0.027), LUAD (*p* = 0.016), SARC (Sarcoma) (*p* = 0.025) and disease-free survival (DFS) for cancers of KICH (*p* = 0.048), LGG (*p* = 0.0000058) within the TCGA project. Additionally, low expression of the *HK2* gene was related to the poor OS for ACC (*p* = 0.022) as shown in Fig. 2a.

### Genetic alteration analysis data

**Figure 3:**
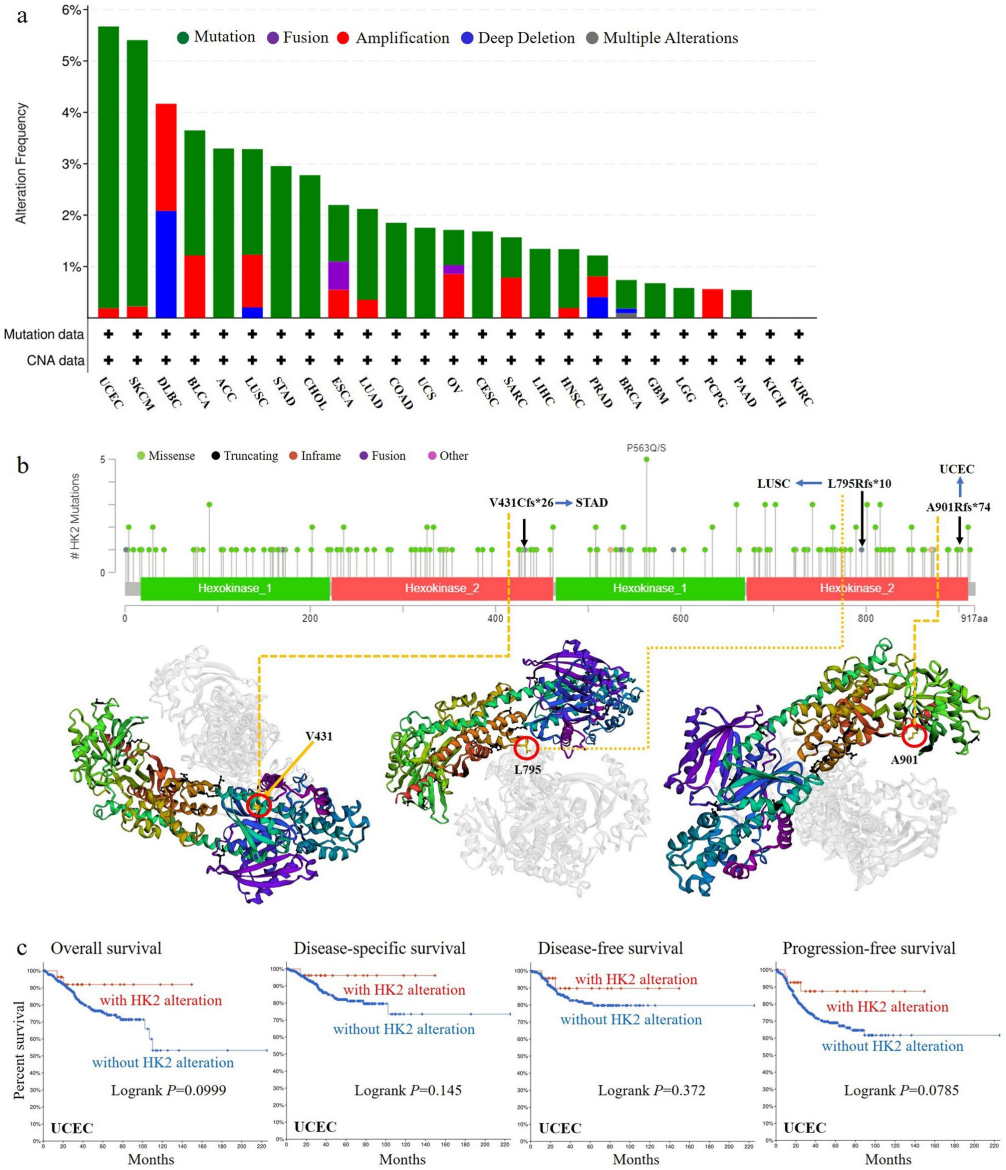
Mutation feature of HK2 in different tumors of TCGA. The mutation features of HK2 for the TCGA tumors were analyzed using the cBioPortal tool. The alteration frequency with mutation type (**a**) and mutation site (**b**) are displayed. The mutation site (V431Cfs*26, L795Rfs*10, and A901Rfs*74) in the 3D structure of HK2 were also displayed (**b**). The potential correlation between mutation status and overall, disease-specific, disease-free, and progression-free survival of UCEC (**c**) was also analyzed using the cBioPortal tool.

The genetic alteration status of HK2 in different tumor samples of the TCGA cohorts was analyzed. As shown in Fig. 3a, the highest alteration frequency of HK2 (> 5%) appears for patients with UCEC and SKCM with “mutation” as the primary type. The “amplification” type and “deep deletion” type of CNA was the primary type in the DLBC, which show an alteration frequency of ~ 2%. Significantly, all PCPG cases with genetic alteration (~ 1% frequency) had “amplification” of HK2 (Fig. 3a). The types, sites and case number of the HK2 genetic alteration are further shown in Fig. 3b. As shown in Fig. 3b, missense mutation of HK2 was the main type of genetic alteration. We can also observe that V431Cfs*26, L795Rfs*10, and A901Rfs*74 alteration were detected in cases of STAD, LUSC, and UCEC, respectively, which were able to induce a frame shift mutation of the HK2 gene, translation from V (Valine) to C (Cystine) at the 431 site, L (Leucine) to R (Arginine) at the 795 site, and A (Alanine) to R (Arginine) at the 901 site of HK2 protein. The 3D structure of HK2 protein was demonstrated in Fig. 3b of the V431 site, L795, and A901 site. Additionally, we explored the association between genetic alteration of HK2 and the survival prognosis of different types of cancers. However, there are no statistical differences between HK2 mutation and the survival of various tumors. The data of Fig. 3c indicate that UCEC cases with altered HK2 seemed to have a survival advantage in overall survival (*p* = 0.0999), disease-specific survival (*p* = 0.145), disease-free survival (*p* = 0.372), and progression-free survival (*p* = 0.0785), compared with cases without HK2 alteration.

### Immune infiltration analysis data

**Figure 4:**
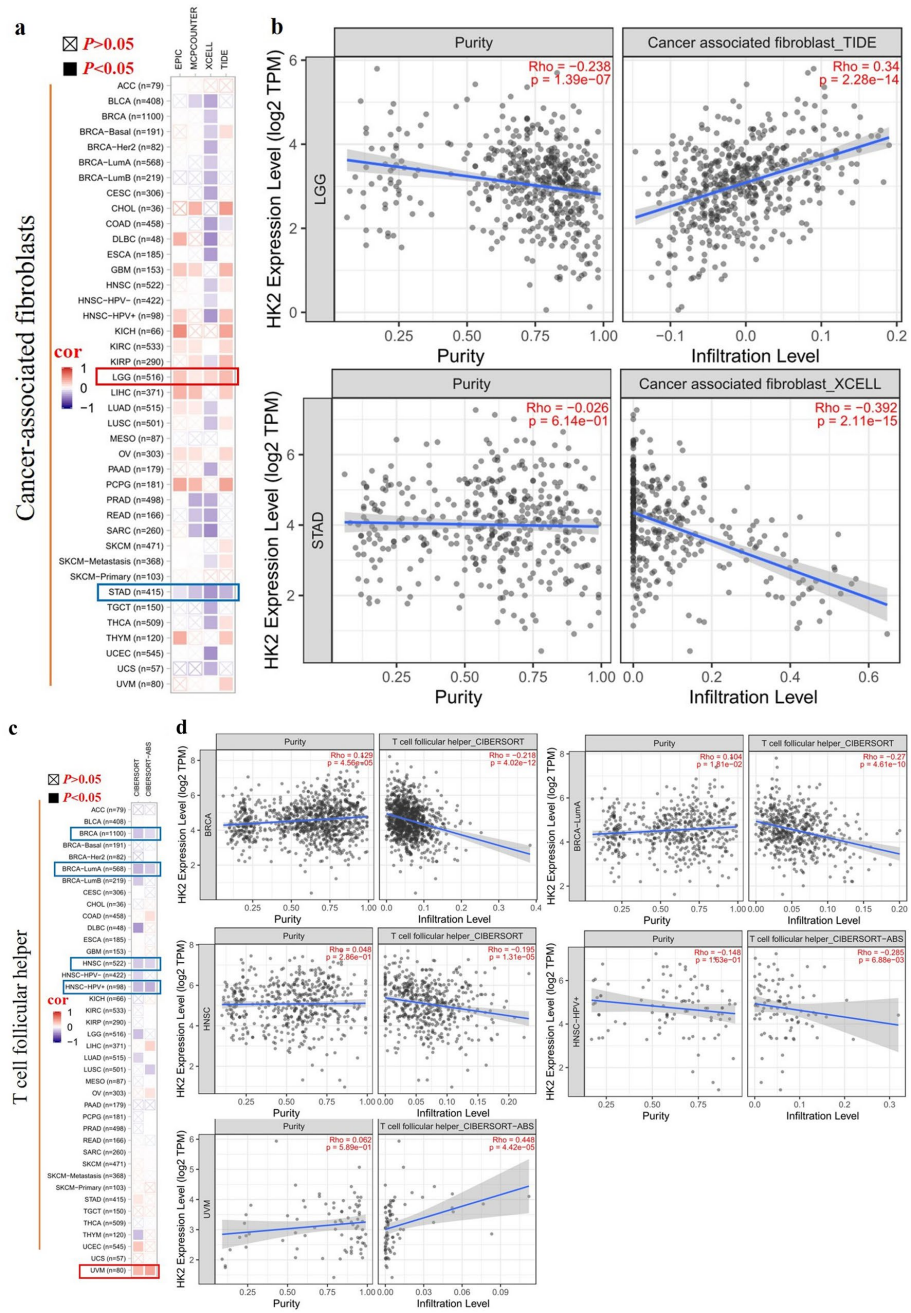
Correlation analysis between *HK2* expression and immune infiltration of cancer-associated fibroblasts and T follicular helper cells. Different algorithms were used to explore the potential correlation between the expression level of the HK2 gene and the infiltration level of cancer-associated fibroblasts and T follicular helper cells across all types of cancer to TCGA.

The tumor microenvironment is closely related to tumor initiation, progression, drug resistance, and metastasis. Tumor-infiltrating immune cells such as cancer-associated fibroblasts (CAFs), tumor-associated macrophages (TAMs), dendritic cells (DCs), myeloid-derived suppressor cells (MDSCs), and T cells are important components of the tumor microenvironment^13,14^. Therefore, we used the TIMER, CIBERSORT, CIBERSORT-ABS, TIDE, XCELL, MCPCOUNTER, and EPIC algorithms to explore the potential relationship between the infiltration of immune cells and HK2 gene expression in different cancer types of TCGA. As demonstrated in Fig. 4a and b, we found a positive correlation between HK2 expression and the infiltration of CAFs for tumors of LGG and a negative correlation of STAD. T follicular helper cell (Tfh) is another important tumor-infiltrating immune cell of the tumor microenvironment^15–17^. As demonstrated in Fig. 4c and d, we observed a positive correlation between HK2 expression and the infiltration of Tfh for tumors of UVM (Uveal melanoma) and a negative correlation between BRCA, BRCA-LumA, HNSC, HNSC-HPV^+^.

### Enrichment analysis of HK-2-related partners

**Figure 5:**
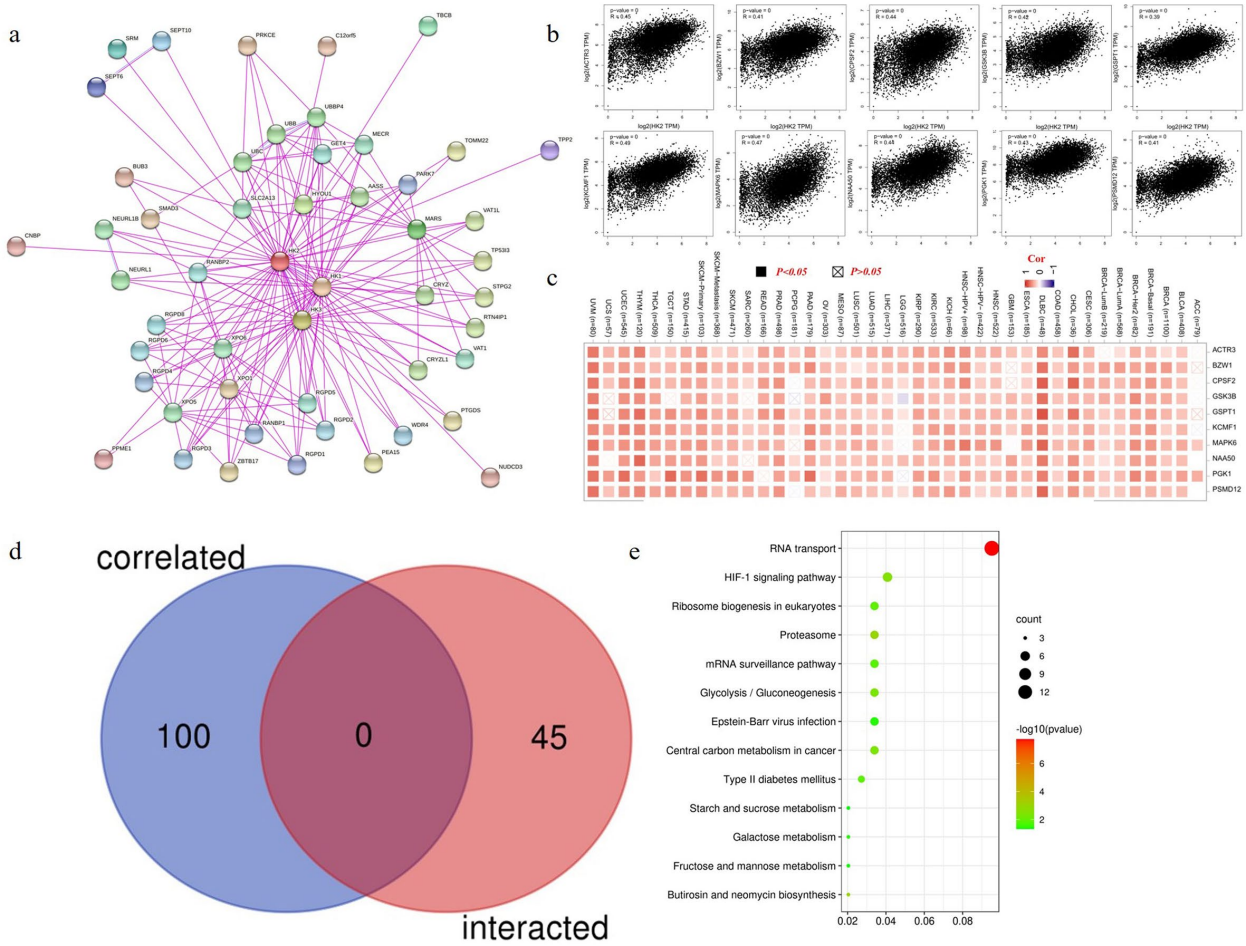
HK2-related gene enrichment analysis. (**a**) The STRING was used to obtain the available experimentally determined HK2-binding proteins. (**b**) Using the GEPIA2 approach, we obtained the top 100 HK2-correlated genes in TCGA projects and analyzed the expression correlation between HK2 and selected targeting genes, including ACTR3, BZW1, CPSF2, GSK3B, GSPT1, KCMF1, MAPK6, NAA50, PGK1, and PSMD12. (**c**) The corresponding heatmap data in the detailed cancer types are displayed. (**d**) An intersection analysis of the HK2-binding and correlated genes was conducted. (**e**) Based on the HK2-binding and interacted genes, a KEGG pathway analysis was performed.

To reveal the mechanism of the *HK2* gene in tumorigenesis, the targeting HK2-binding proteins and the *HK2* expression-associated genes for pathway enrichment analysis were screened. With the help of the STRING tool, we got a total of 50 HK2-binding proteins. The interaction network of these proteins was shown in Fig. 5a. We used the GEPIA2 tool to combine all tumor expression data of TCGA and obtained the top 100 genes that related to HK2 expression. As shown in Fig. 5b, the HK2 expression was positively correlated with that of ACTR3 (Actin related protein 3) (R = 0.45), BZW1 (Basic leucine zipper and W2 domains 1) (R = 0.41), CPSF2 (Cleavage and polyadenylation specific factor 2) (R = 0.44), GSK3B (Glycogen synthase kinase 3 beta) (R = 0.42), GSPT1 (G1 to S phase transition 1) (R = 0.39), KCMF1 (Potassium channel modulatory factor 1) (R = 0.49), MAPK6 (Mitogen-activated protein kinase 6) (R = 0.47), NAA50 (N-alpha-acetyltransferase 50) (R = 0.44), PGK1 (Phosphoglycerate kinase 1) (R = 0.43), and PSMD12 (Proteasome 26S subunit, non-ATPase 12) (R = 0.41) genes (all *p* < 0.001). The heatmap data was shown in Fig. 5c demonstrating a positive correlation between HK2 and the above ten genes in most of the cancer types. The cross-analysis of the two groups revealed no common member. (Fig. 5d).

We united the two datasets to carry out KEGG enrichment analyses, demonstrating that “RNA transport” might participate in the effect of HK2 on tumor pathogenesis as shown in Fig. 5e.

## Discussion

It has been reported that HK2 plays an important role in tumor biology^18,19^. Whether HK2 plays a role in different tumors through common molecular mechanisms is unclear. Through literature searching, we failed to find any articles that made a pan-cancer analysis of HK2 from a holistic perspective. So, it is necessary to make a global analysis of HK2 in tumors. Therefore, we examined HK2 genes in a total of 33 different tumors based on TCGA, CPTAC, and GEO database data, as well as molecular characteristics of gene expression, survival prognosis, genetic alteration, or immune infiltration.

Gene expression of HK2 was highly expressed in most tumors except BRCA, LIHC, and PCPG. However, HK2 was lower expressed in tumor tissues than in normal tissues of COAD (Colon adenocarcinoma), KICH, READ (Rectum adenocarcinoma), and THYM. Similarly, the survival prognosis analysis data of the HK2 gene suggested distinct conclusions for different tumors.

For breast cancer, the expression of HK2 total protein was higher in tumor tissue than in normal tissue. However, elevated HK2 expression did not affect the overall survival of breast cancer patients. In MDA-MB-453 xenografts, researchers have demonstrated that HK2 expression is decreased in tumors responding to trastuzumab^20^. However, in vitro study showed that knocking down HK2 did not enhance doxorubicin-induced apoptosis^21^. A retrospective study has revealed that patients with high HK2 expression have poor survival in patients with brain metastases of breast cancer^21^. So, the role of HK2 in breast cancer deserves to be further investigated.

For liver cancer, according to the bioinformatics analysis, we found that there were no differences in gene and protein expression of HK2 in tumor tissue than in normal tissue. However, previous studies have demonstrated that compared with non-dysplastic cirrhosis, the expression level of HK2 is higher in liver cell change/dysplasia in cirrhosis and hepatocellular carcinoma^22,23^. The higher expression of HK2 is also associated with the inferior overall survival of LIHC as shown in Fig. 2 and other research^24^. Previous study and bioinformatics analysis have revealed that differences in tumor HK2 expression were associated with cancer stage (*p* = 0.001)^24^. HK2 has been shown to interact with the mitochondria and contribute to liver cancer progression^25^. Recently, a fifth hexokinase, hexokinase domain containing 1 (HKDC1), was shown to have significant overexpression in liver cancer compared to healthy liver tissue^26^. Studies revealed that inhibiting HK2-mediated glycolysis can enhance the anti-hepatocellular carcinoma activity of sorafenib^27,28^. HK2 may be a possible therapeutic target for liver cancer.

For lung adenocarcinoma, high HK2 protein expression is associated with poor overall survival as shown in Figs. 1 and 2. Hypoxia can induce HK2 gene expression which protects human epithelial-like A549 cells against oxidative injury^29,30^. In vitro studies have shown that HK2 inhibition can suppress lung tumor growth^31,32^. Depletion of HK2 confers sensitization to chemotherapeutic drugs^33^. A recent study has revealed that HK2 identifies a novel circulating tumor cell population associated with poor prognosis in lung cancer patients^34^. In addition to its role in metabolic regulation, recent study have shown that HK2 can increase stemness of small cell lung cancer cells by increasing ubiquitin-specific protease 11-mediated CD133 stability^35^.

Most of the studies have revealed that lower expression of HK2 is associated with better overall survival such as CESC, KIRP, LGG, LIHC, LUAD, and SARC^24,34,36–38^. But for adrenocortical carcinoma, low expression of the *HK2* gene was related to the poor OS for ACC (*p* = 0.022) as shown in Fig. 2a. Duan et al. reported that cytoplasmic expression of HK1 was significantly higher in ACC than in normal adrenal cortical tissue samples^39^. In cervical carcinoma cells, the HK1 but not HK2 knockdown induced a phenotypic change characteristic of epithelial-mesenchymal transition, which accelerated tumor growth and metastasis^40^. As suggested in recent studies, HK1 is also involved in the carcinogenesis of some solid tumors^41–43^.

In this paper, we first reported the relationship between HK2 gene alteration and the survival prognosis of different tumor types. However, there was no statistically significant difference in survival between HK2 mutations and various tumors. In addition, we analyzed the information of HK2-binding proteins and HK2 expression-related genes across all tumors for a series of enrichment analyses and determined that “RNA transport” might be involved in the effect of HK2 on tumor pathogenesis. We adopted multiple immune deconvolution methods to discuss a statistically negative correlation between *HK2* expression and the immune infiltration level of T follicular helper cells in the tumors of BRCA, BRCA-LumA, HNSC, and HNSC-HPV^+^. In addition, our findings first suggested the association of *HK2* expression had a statistically positive correlation with infiltration of CAFs in LGG and a negative correlation in STAD.

In this paper, we first reported the relationship between HK2 gene alteration and the survival prognosis of different tumor types. However, there was no statistically significant difference in survival between HK2 mutations and various tumors. In addition, we analyzed the information of HK2-binding proteins and HK2 expression-related genes across all tumors for a series of enrichment analyses and determined that “RNA transport” might be involved in the effect of HK2 on tumor pathogenesis. We adopted multiple immune deconvolution methods to discuss a statistically negative correlation between HK2 expression and the immune infiltration level of T follicular helper cells in the tumors of BRCA, BRCA-LumA, HNSC, and HNSC-HPV^+^. In addition, our findings first suggested the association of HK2 expression had a statistically positive correlation with infiltration of CAFs in LGG and a negative correlation in STAD.

In conclusion, our first pan-cancer analysis of HK2 showed that the expression of HK2 in most tumors was different from that in normal tissues, and was statistically correlated with clinical prognosis and immune cell infiltration across multiple tumors. Different tumors may lead to different conclusions, so more research is needed. The above results can help us understand the role of HK2 in tumorigenesis from the perspective of clinical tumor samples.

## Supplementary Information

Supplementary Information 1.

Supplementary Information 2.

Supplementary Information 3.

Supplementary Information 4.

Supplementary Information 5.

Supplementary Information 6.

Supplementary Information 7.

## Data Availability

All data generated or analyzed during this study are included in this article and its supplementary information files. These data are also available in the following databases (http://timer.cistrome.org/, http://gepia2.cancer-pku.cn/#analysis, http://ualcan.path.uab.edu/analysis-prot.html, https://www.proteinatlas.org/humanproteome/pathology, https://www.cbioportal.org/, https://string-db.org/, http://bioinformatics.psb.ugent.be/webtools/Venn/, https://david.ncifcrf.gov/, http://www.bioinformatics.com.cn).
