# Supplementary material for: A pan-cancer analysis of the role of hexokinase II (HK2) in human tumors
Source: Sci Rep. 2022 Nov 5;12:18807. doi: 10.1038/s41598-022-23598-8 (PMC9637150; doi:10.1038/s41598-022-23598-8)

Fig 1a: The expression status of the *HK2* gene in different cancers or specific cancer subtypes was analyzed through TIMER2. * *P*<0.05; ** *P*<0.01; *** *P*<0.001.


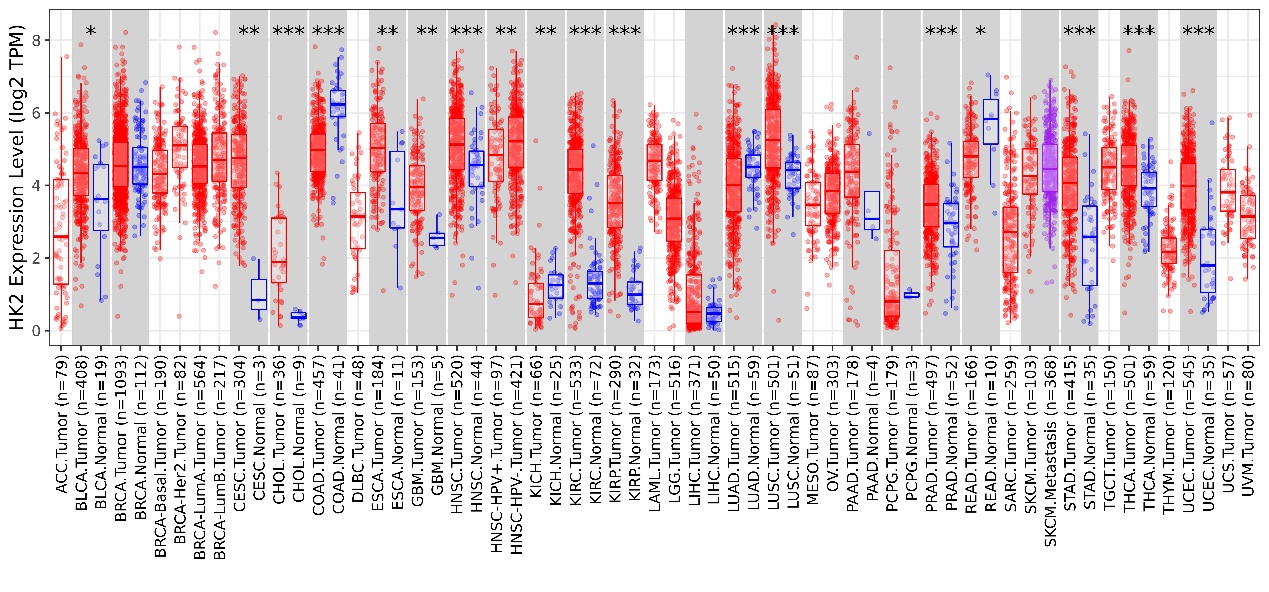


Fig 1b: For the type of ACC, LGG, THYM, and UCS in the TCGA project, the corresponding normal tissues of the GTEx database were included as controls. The box plot data were supplied. ** *P*<0.01.


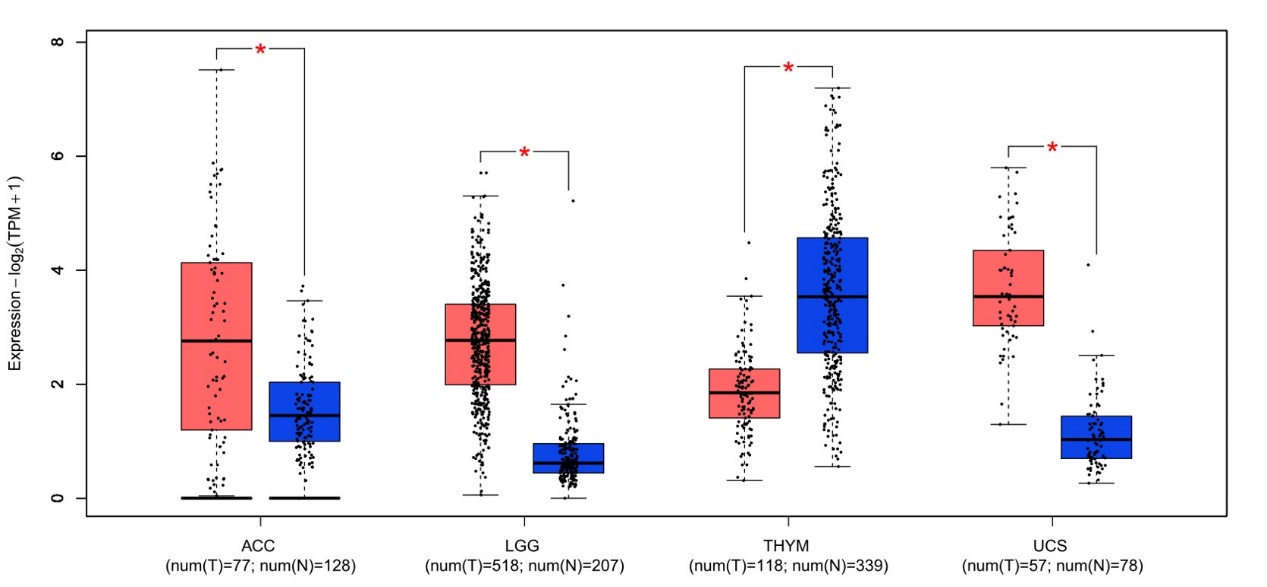


Fig 1c: The expression level of HK2 total protein based on the CPTAC dataset between normal tissue and primary tissue

Breast cancer


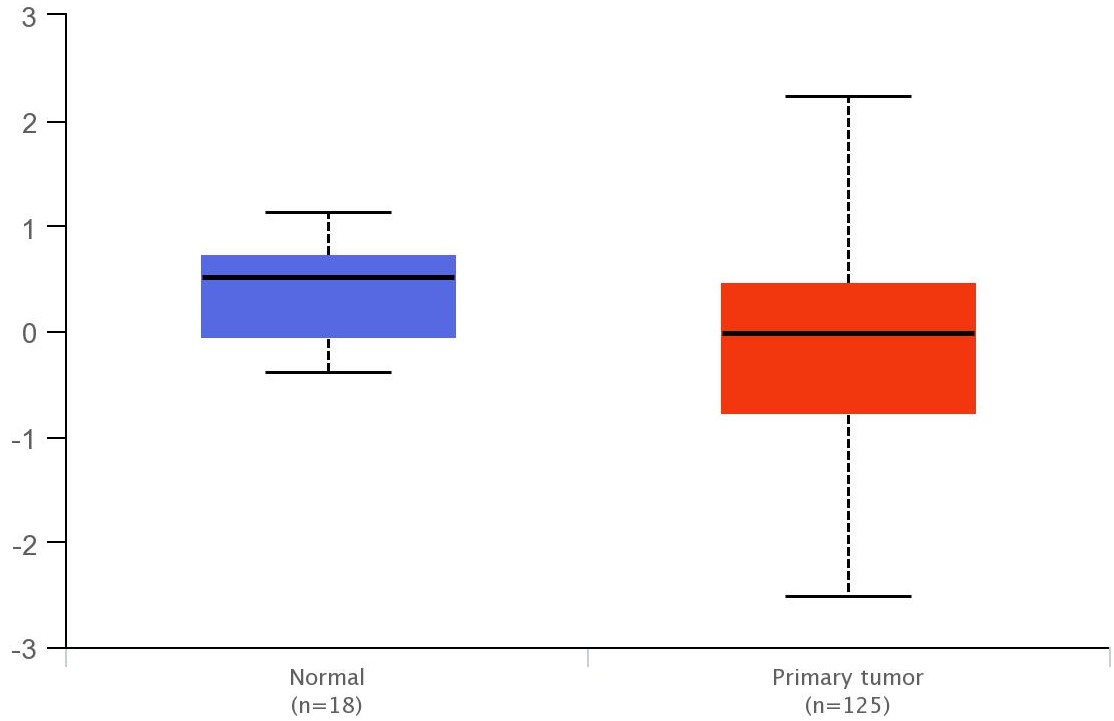


KIRC


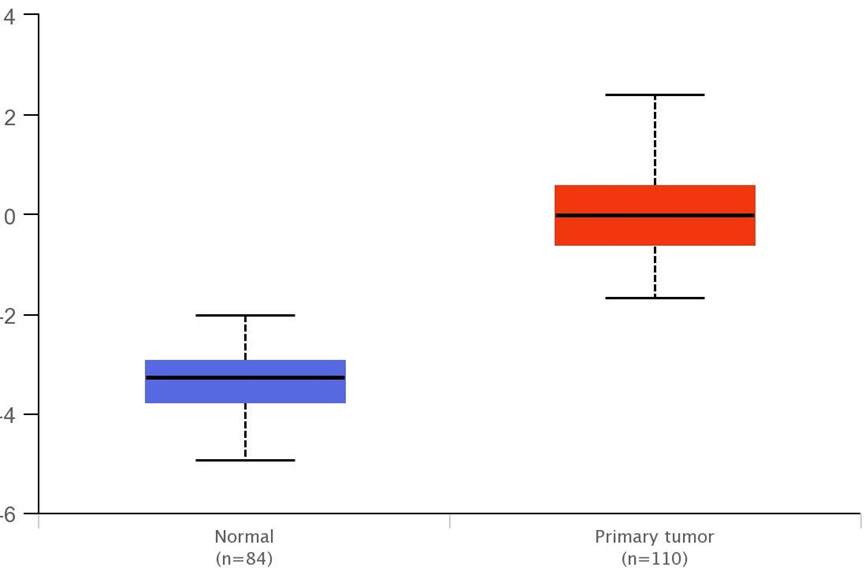


Colon cancer


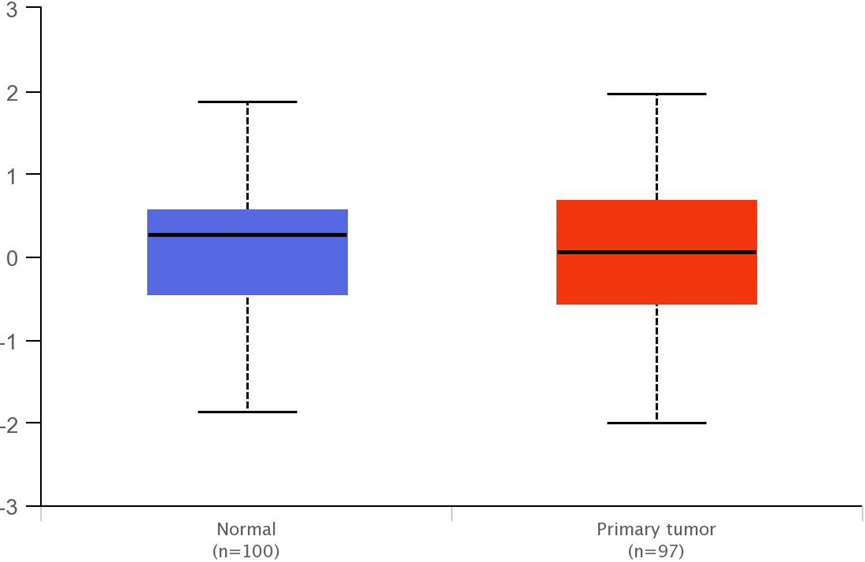


LUAD


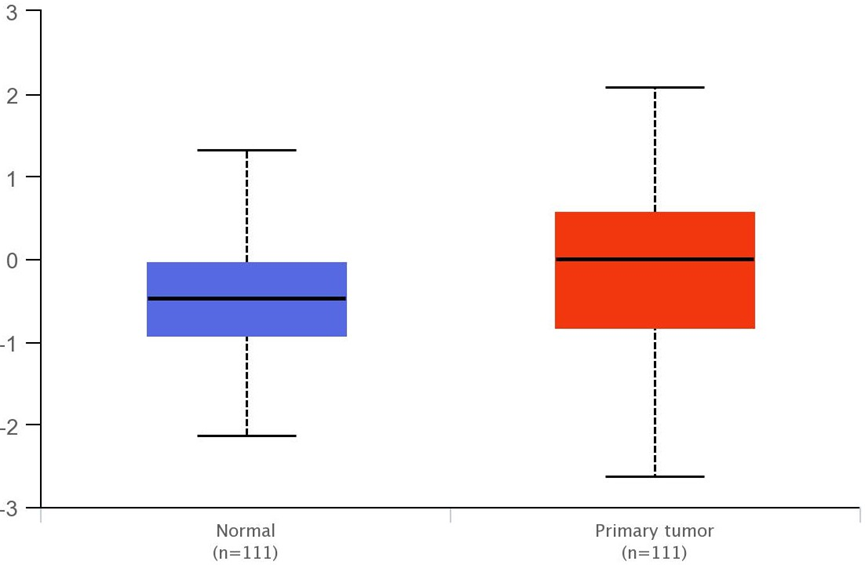


Ovarian cancer


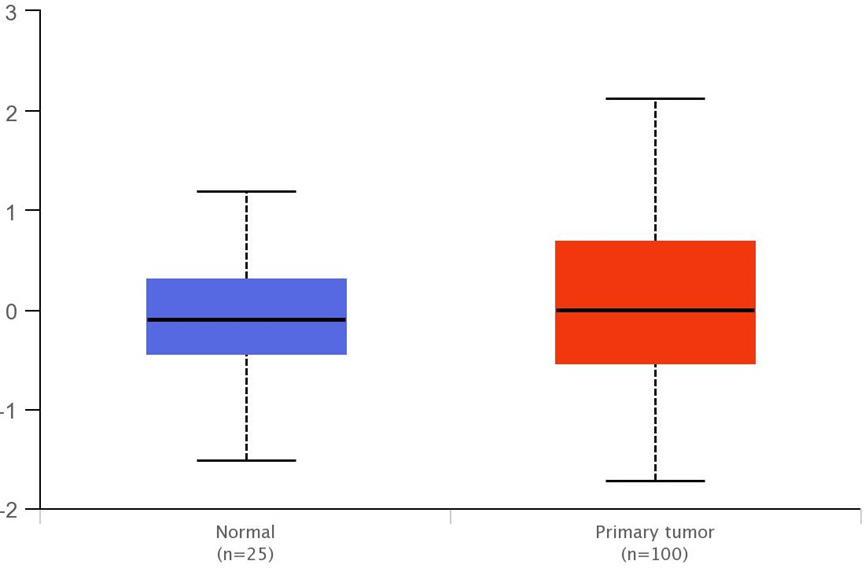


UCEC


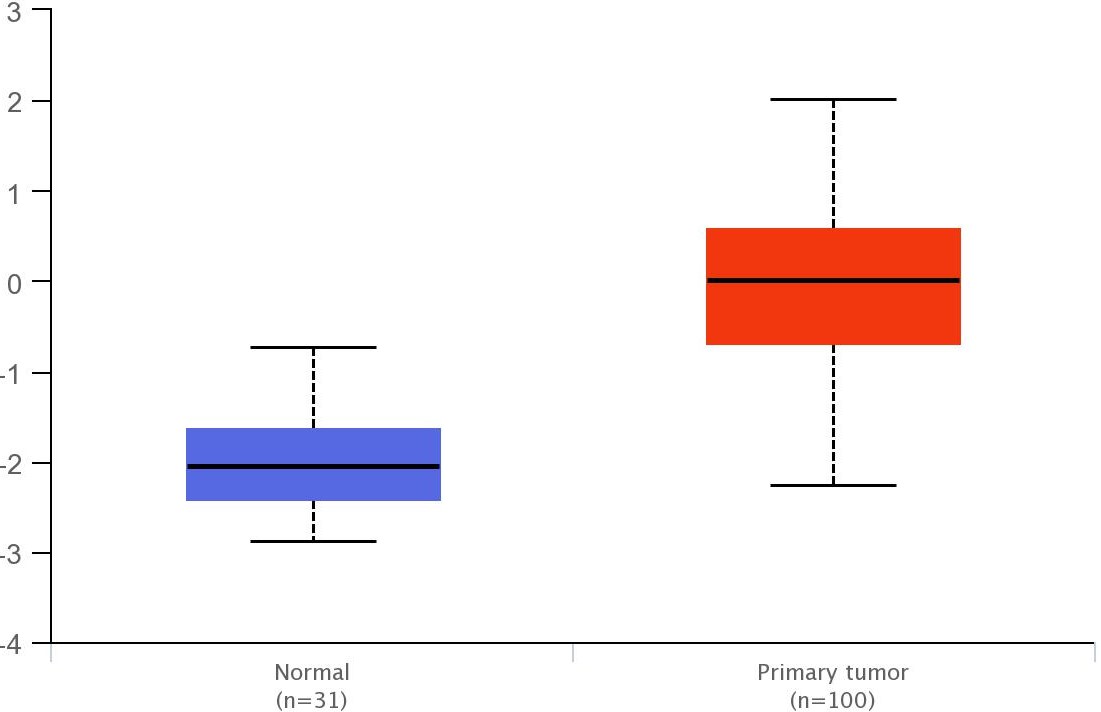


Fig 1d: The expression level of HK2 total protein based on the HPA dataset between normal tissue and primary tissue

Breast cancer


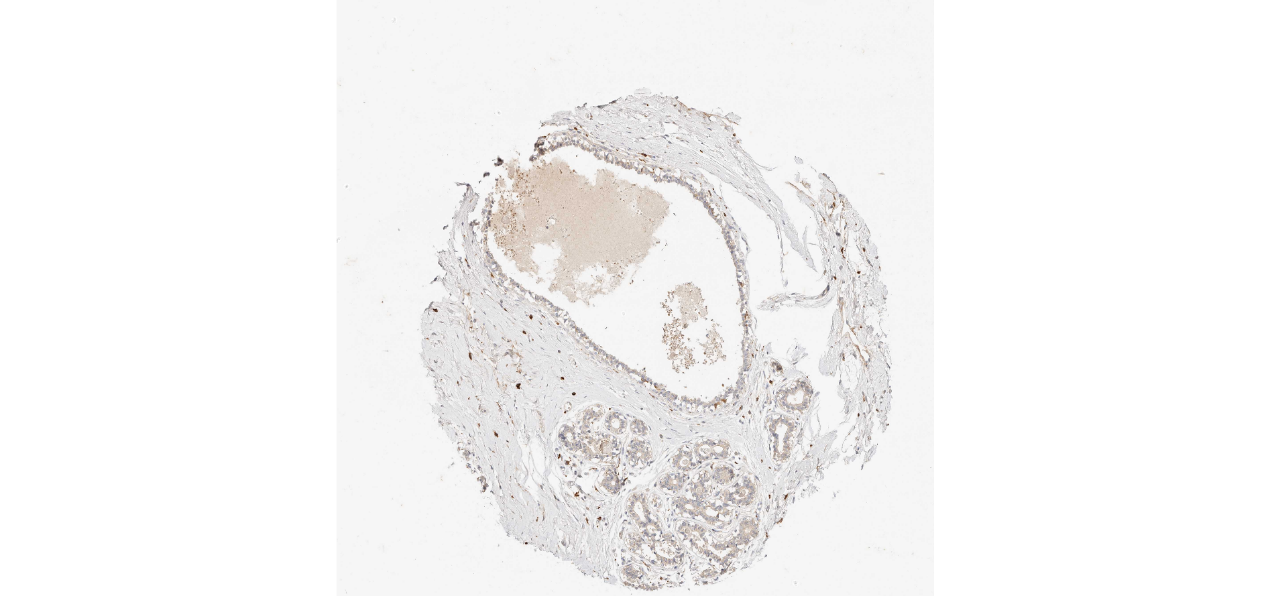

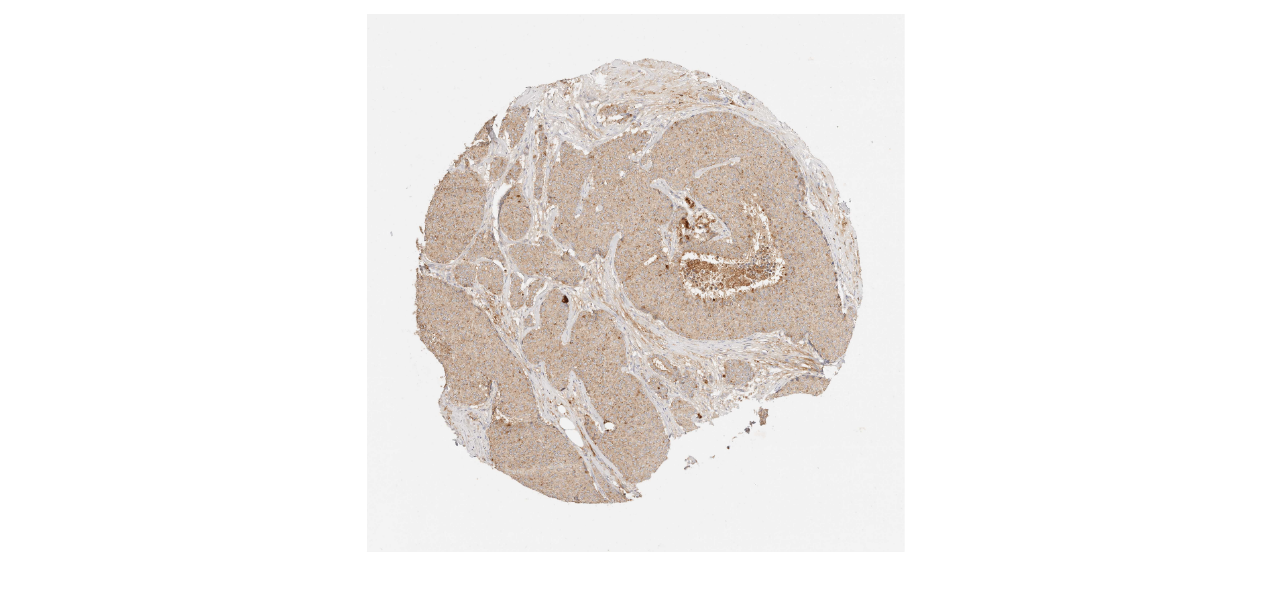


Renal cancer


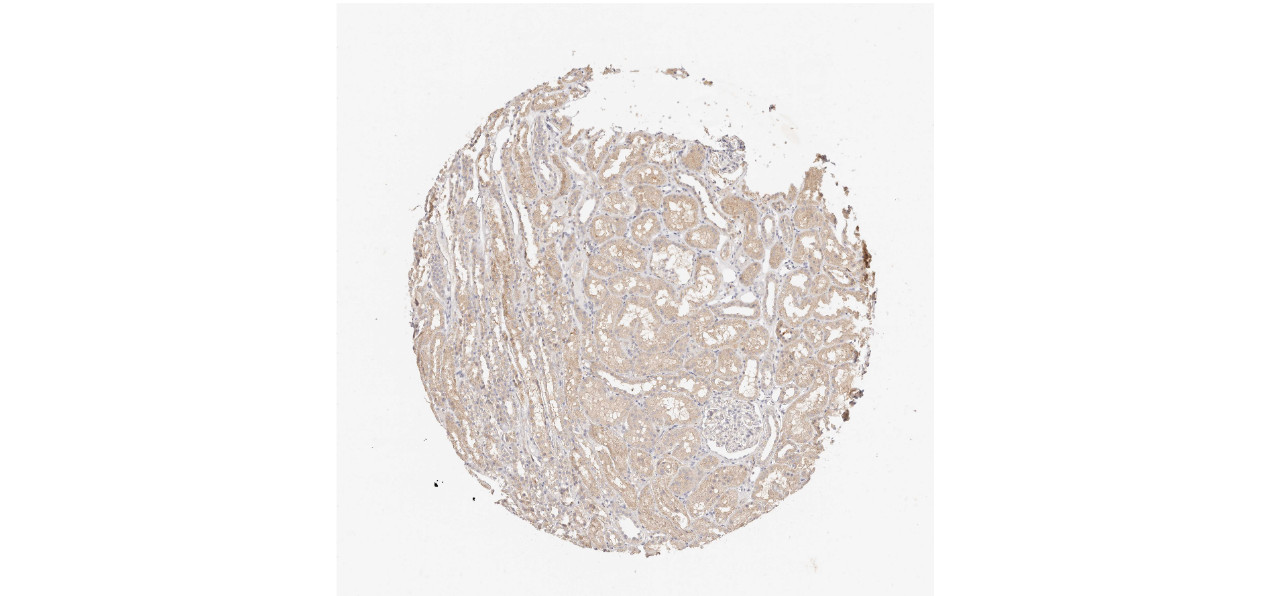


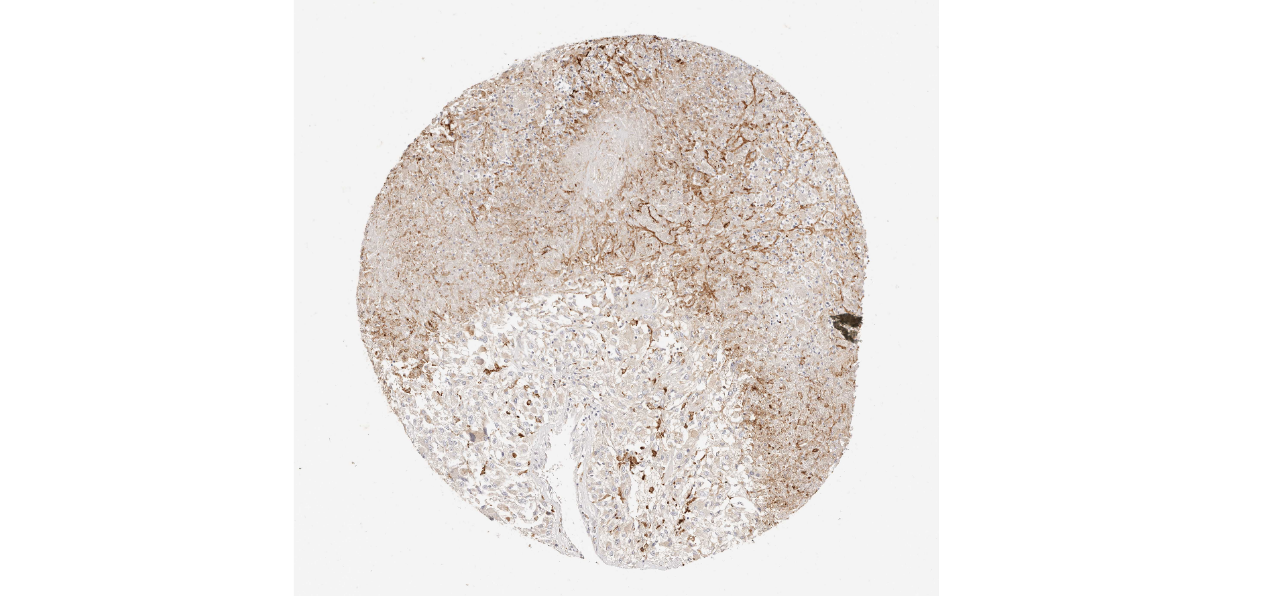


Colon cancer


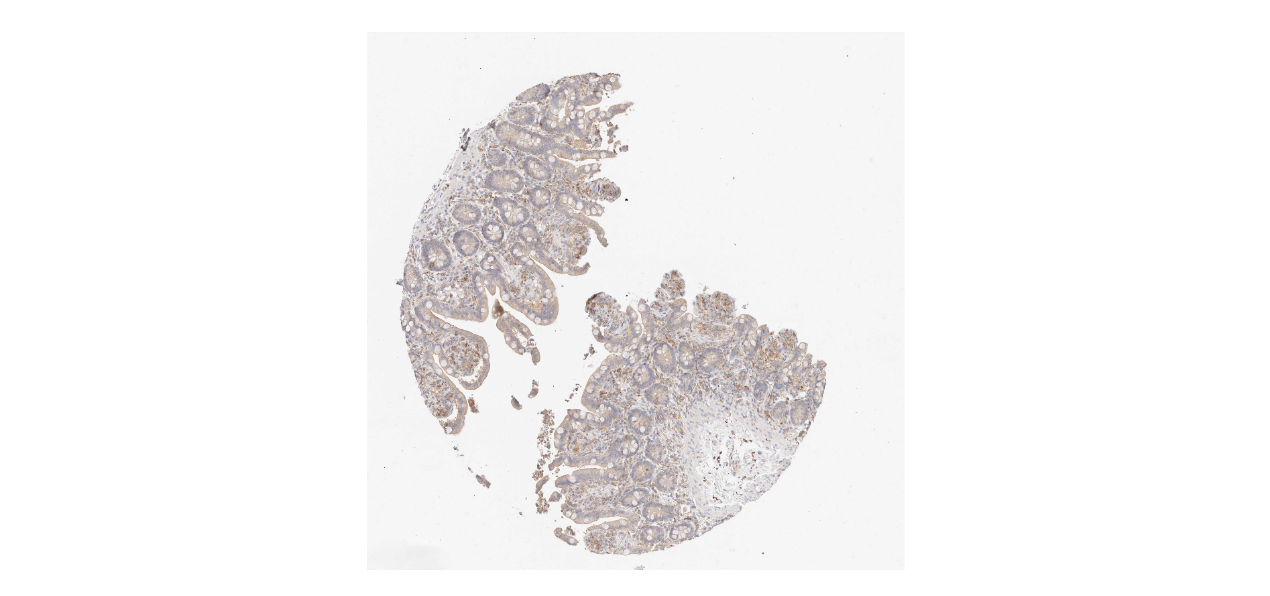


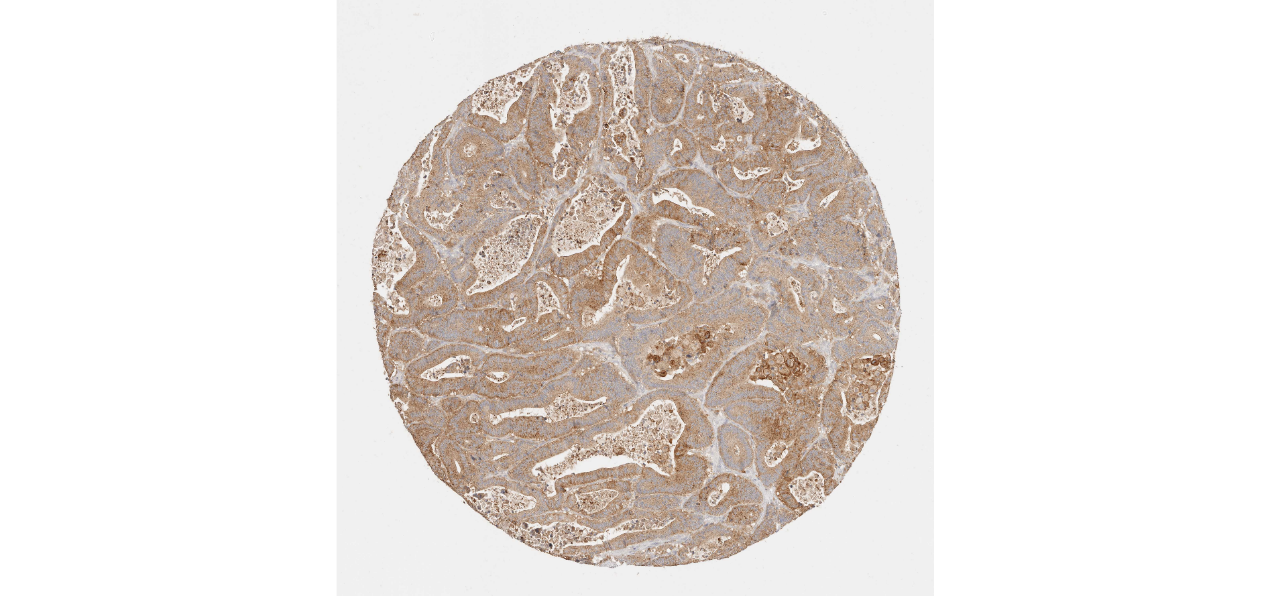


Lung cancer


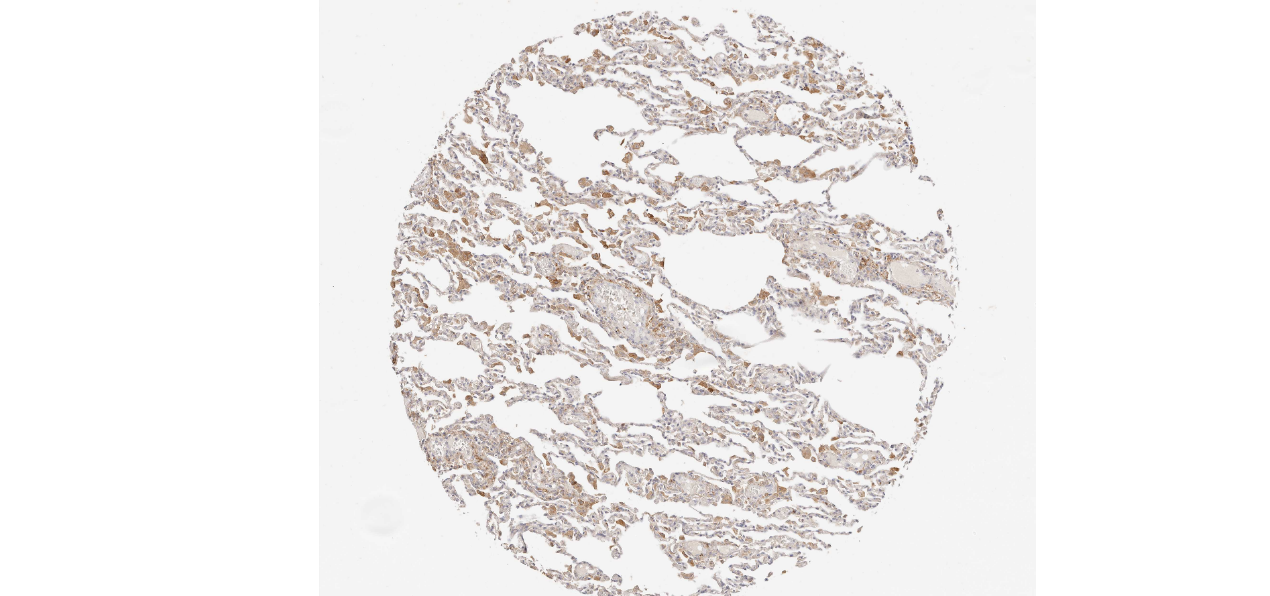


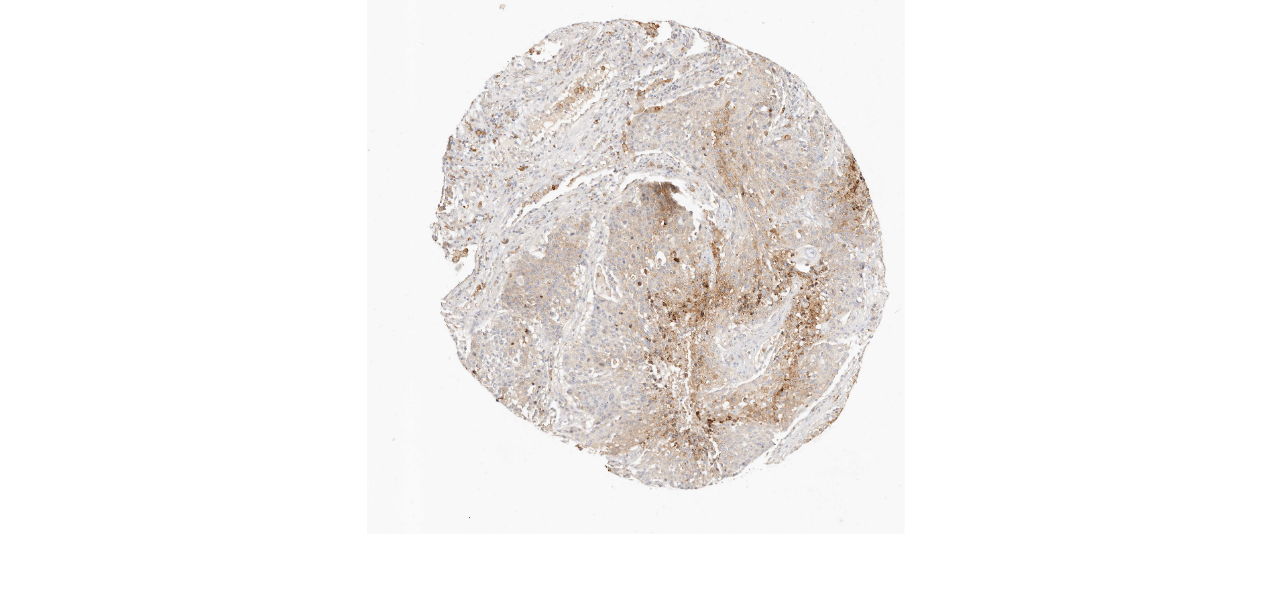


Ovarian· cancer


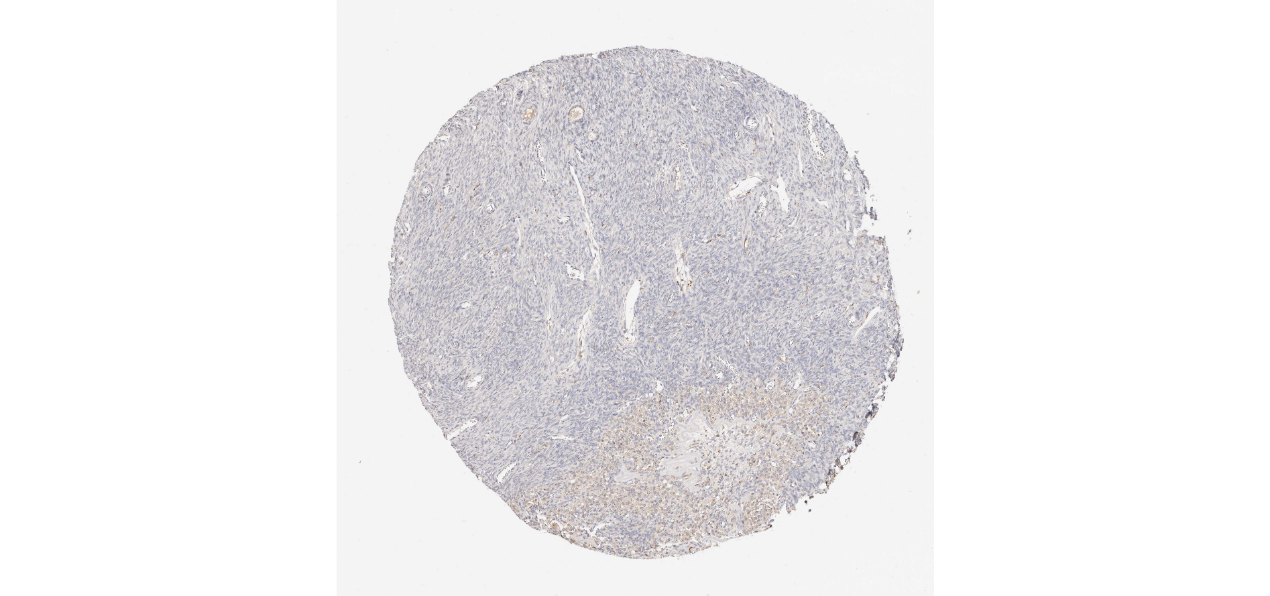


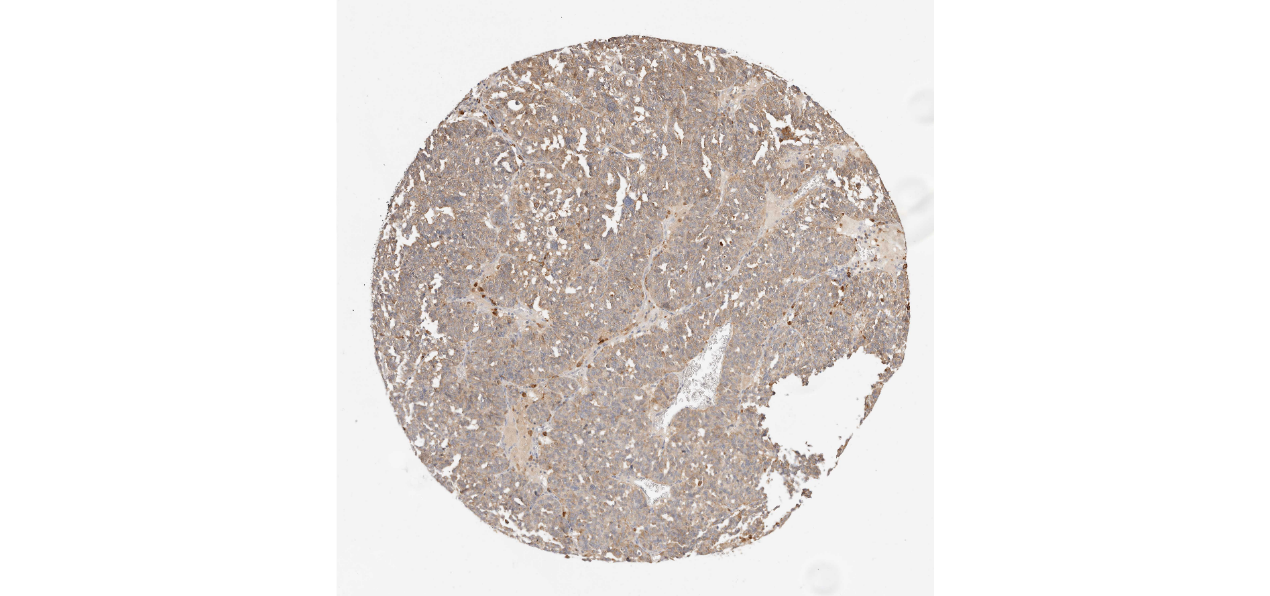


Endometrial cancer


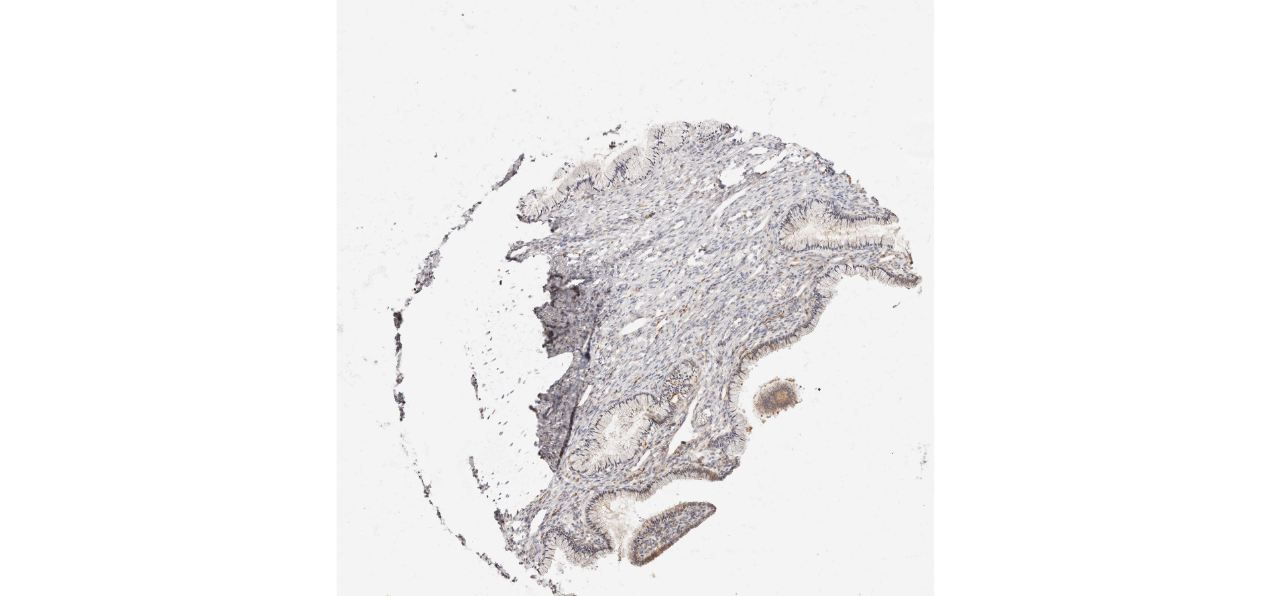


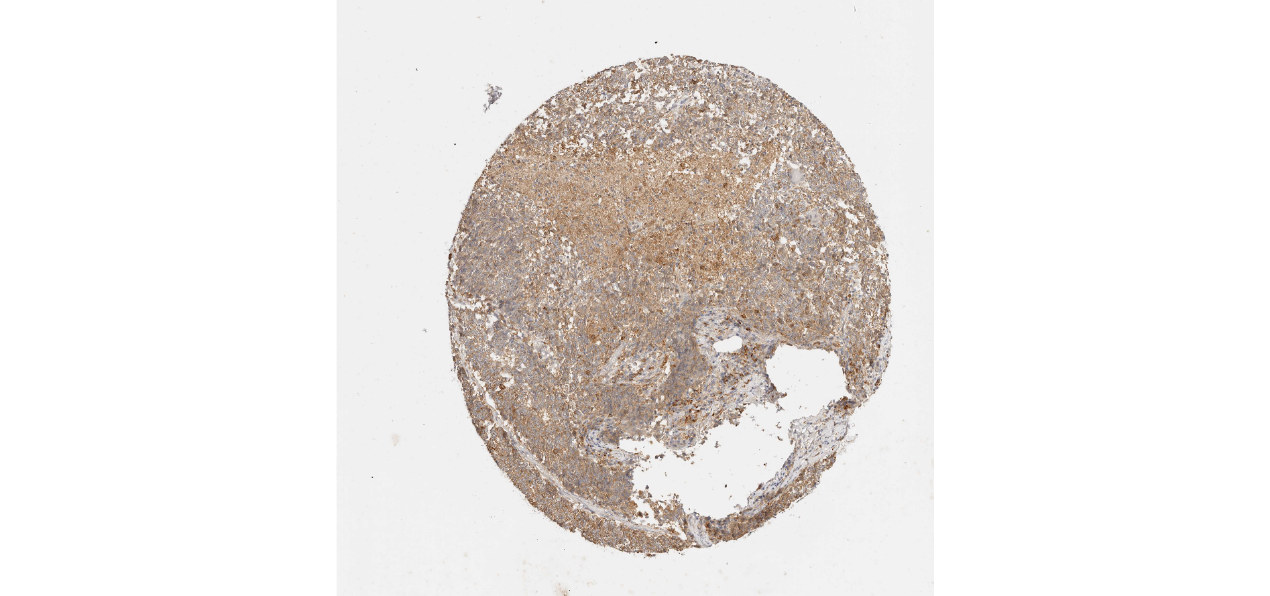


Fig 1e: Based on the TCGA data, the expression levels of the *HK2* gene were analyzed by the main pathological stages (stage I, stage II, stage III, and stage IV) of CESC, KICH, LIHC, ovarian cancer, and PAAD. Log2 (TPM+1) was applied for the log-scale.

CESC


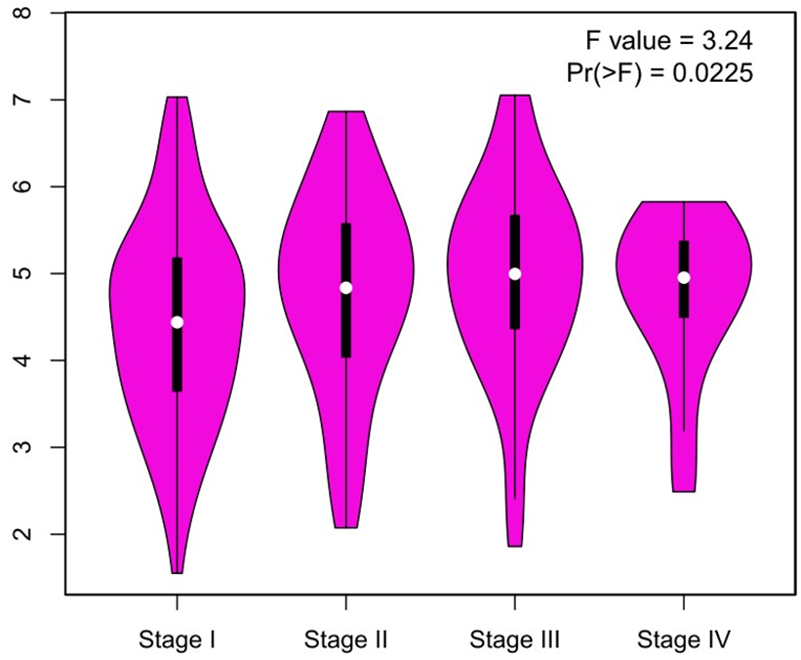


KICH


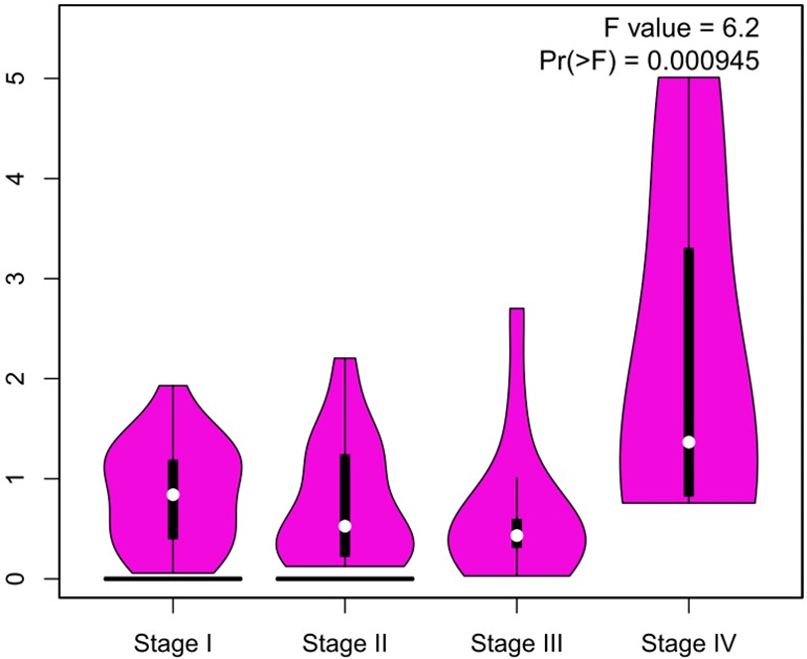


LIHC


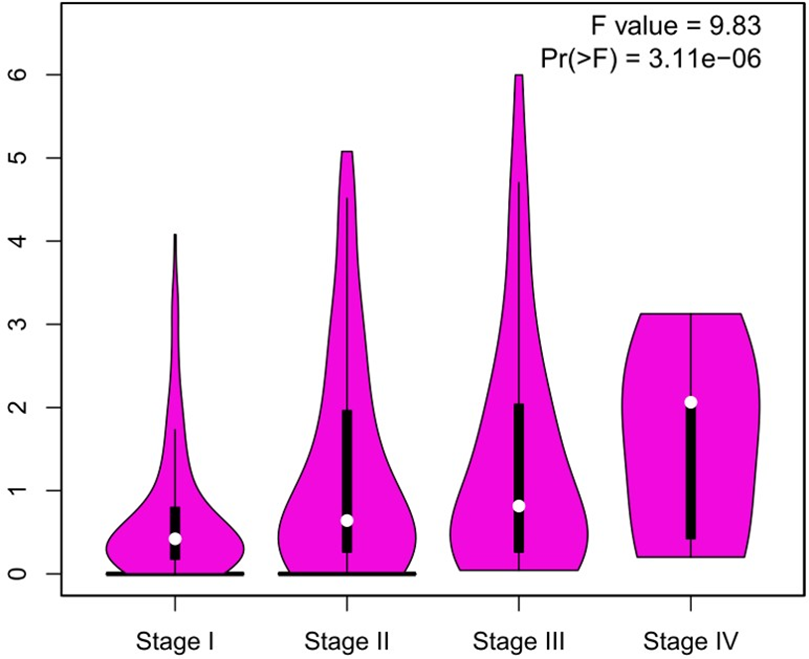


OV


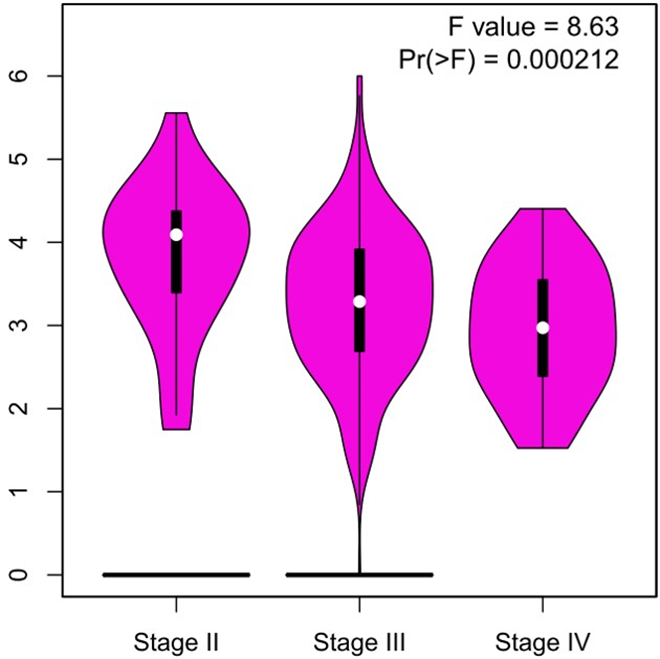


PAAD


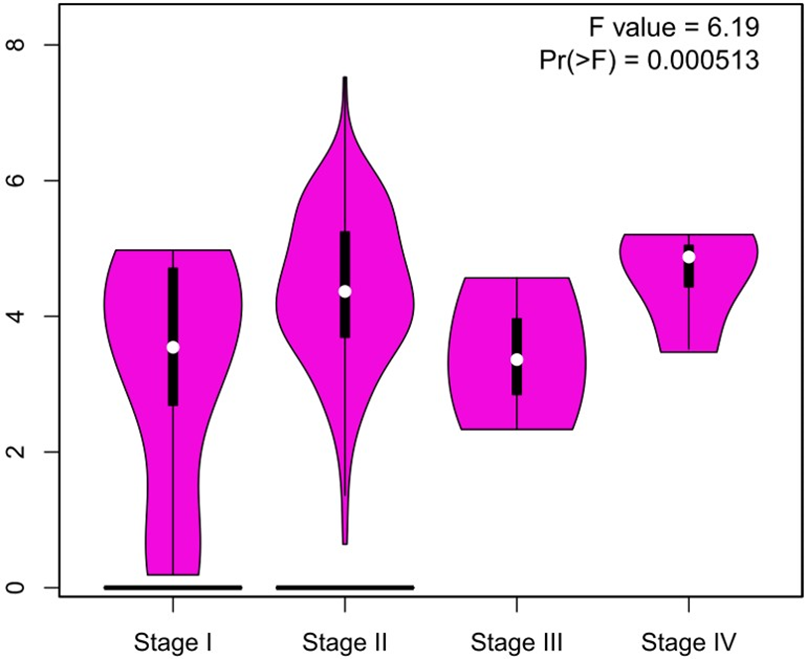

Supplement: Supplementary file 1 — Supplementary Information 1. [file 41598_2022_23598_MOESM1_ESM.docx]
