# Supplementary material for: A pan-cancer analysis of the role of hexokinase II (HK2) in human tumors
Source: Sci Rep. 2022 Nov 5;12:18807. doi: 10.1038/s41598-022-23598-8 (PMC9637150; doi:10.1038/s41598-022-23598-8)

Fig 2a: Correlation between *HK2* gene expression and overall survival.


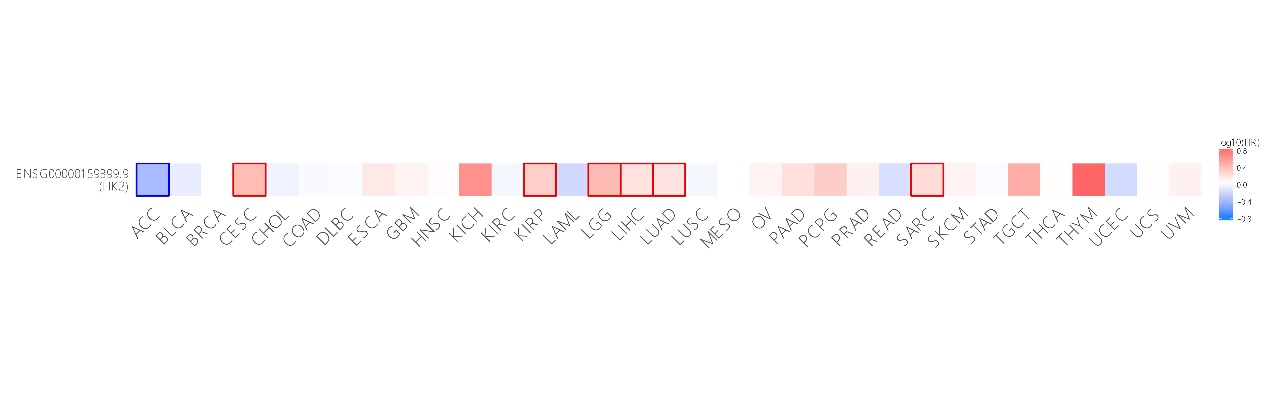


The survival map and Kaplan-Meier curves with positive results are given

ACC


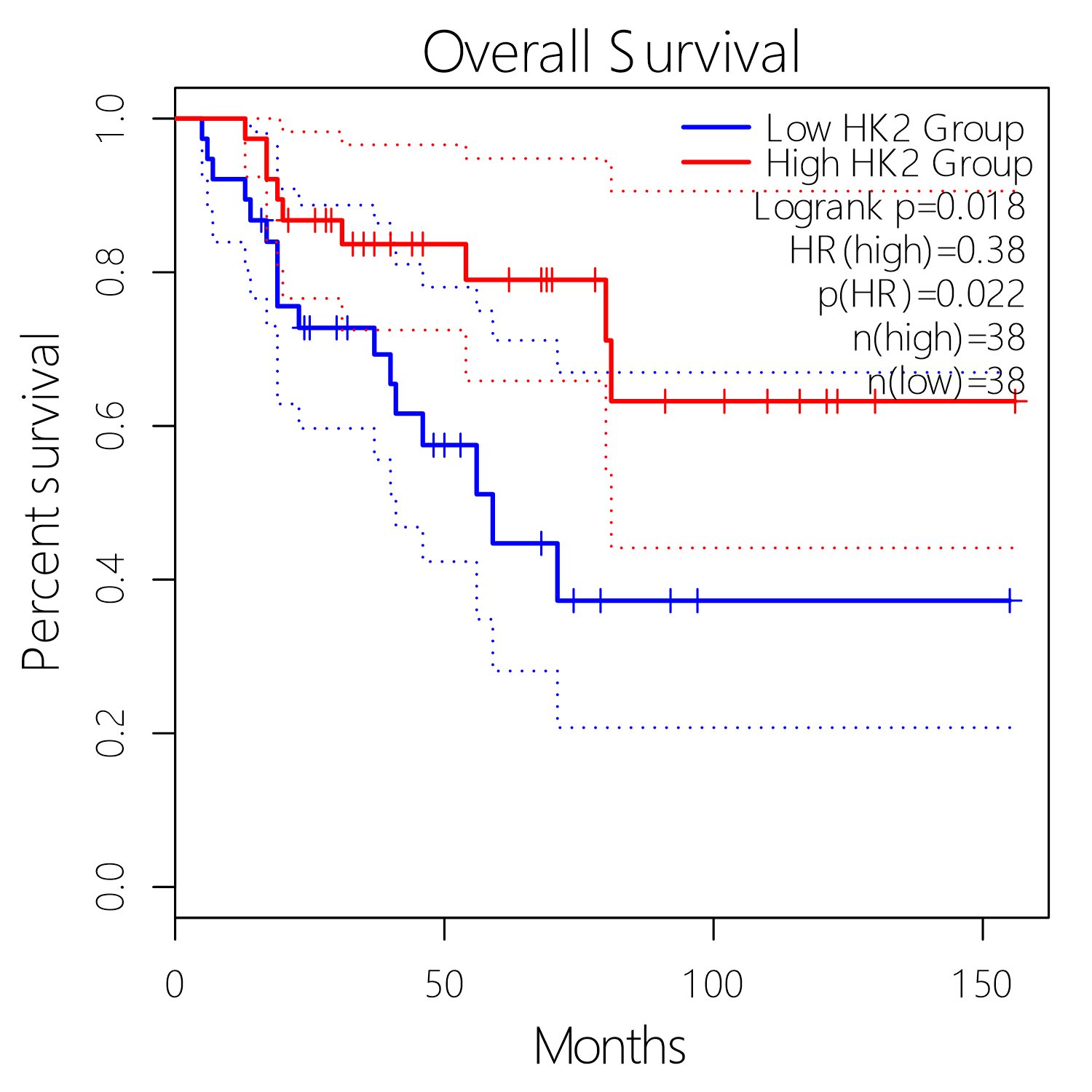


CESC


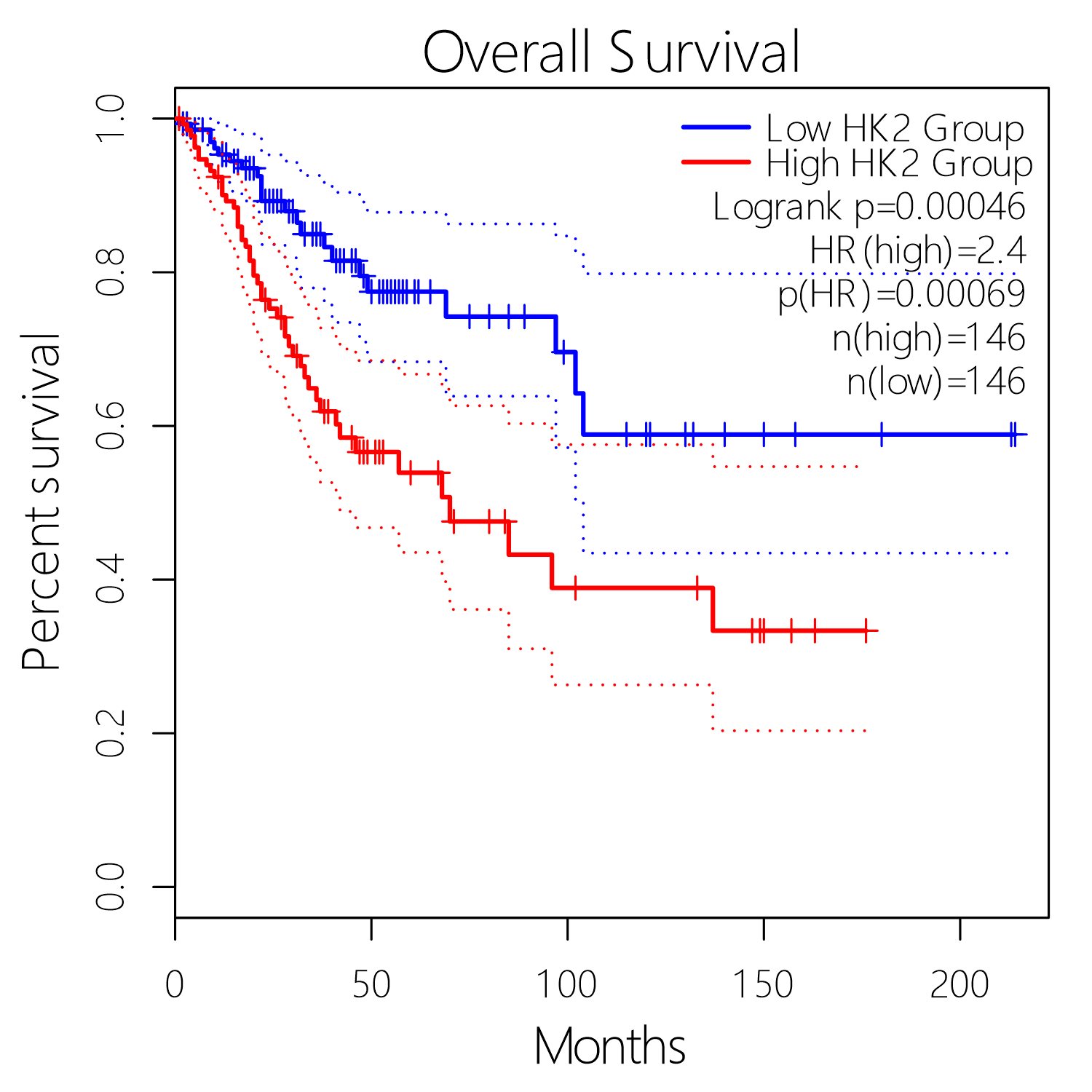


KIRP


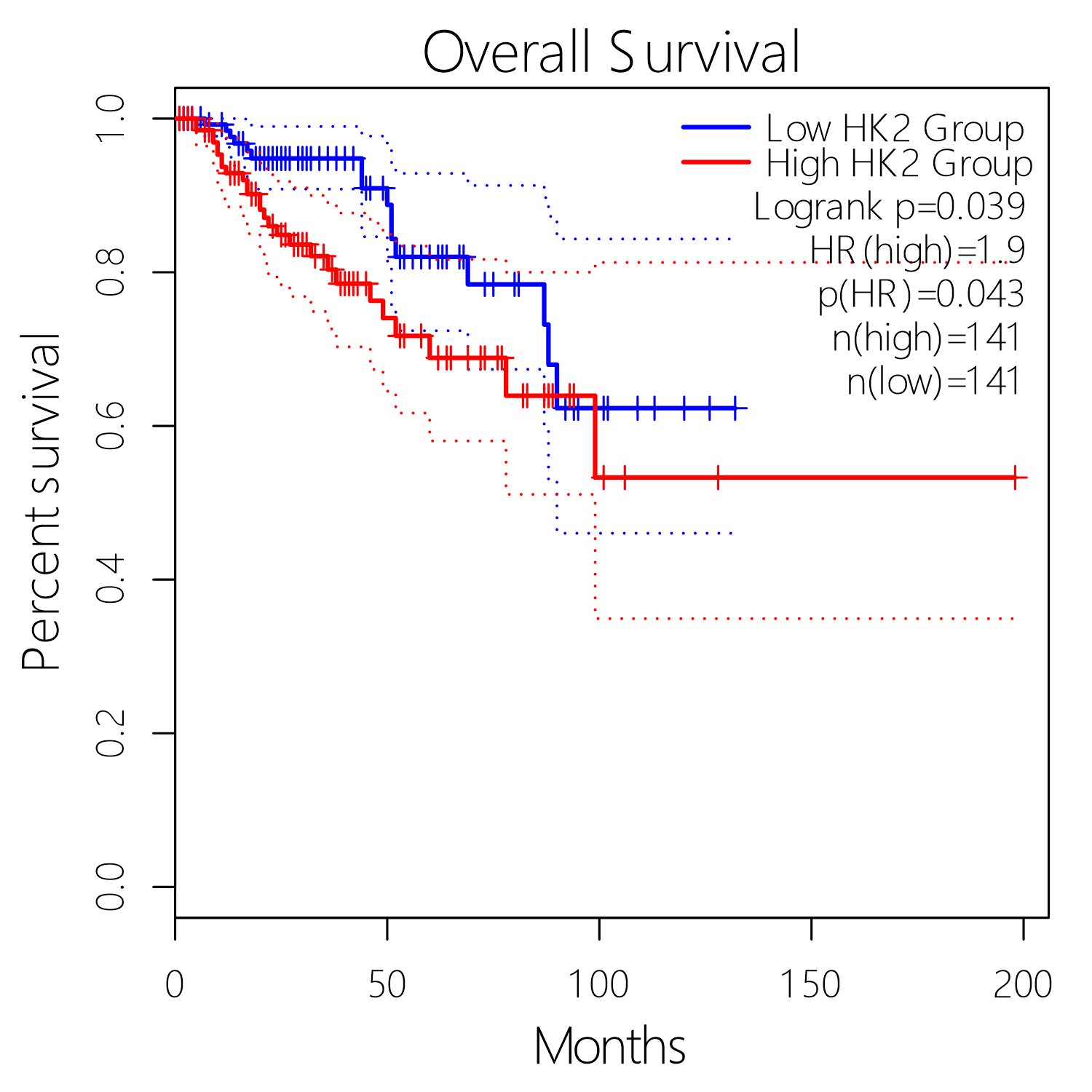


LGG


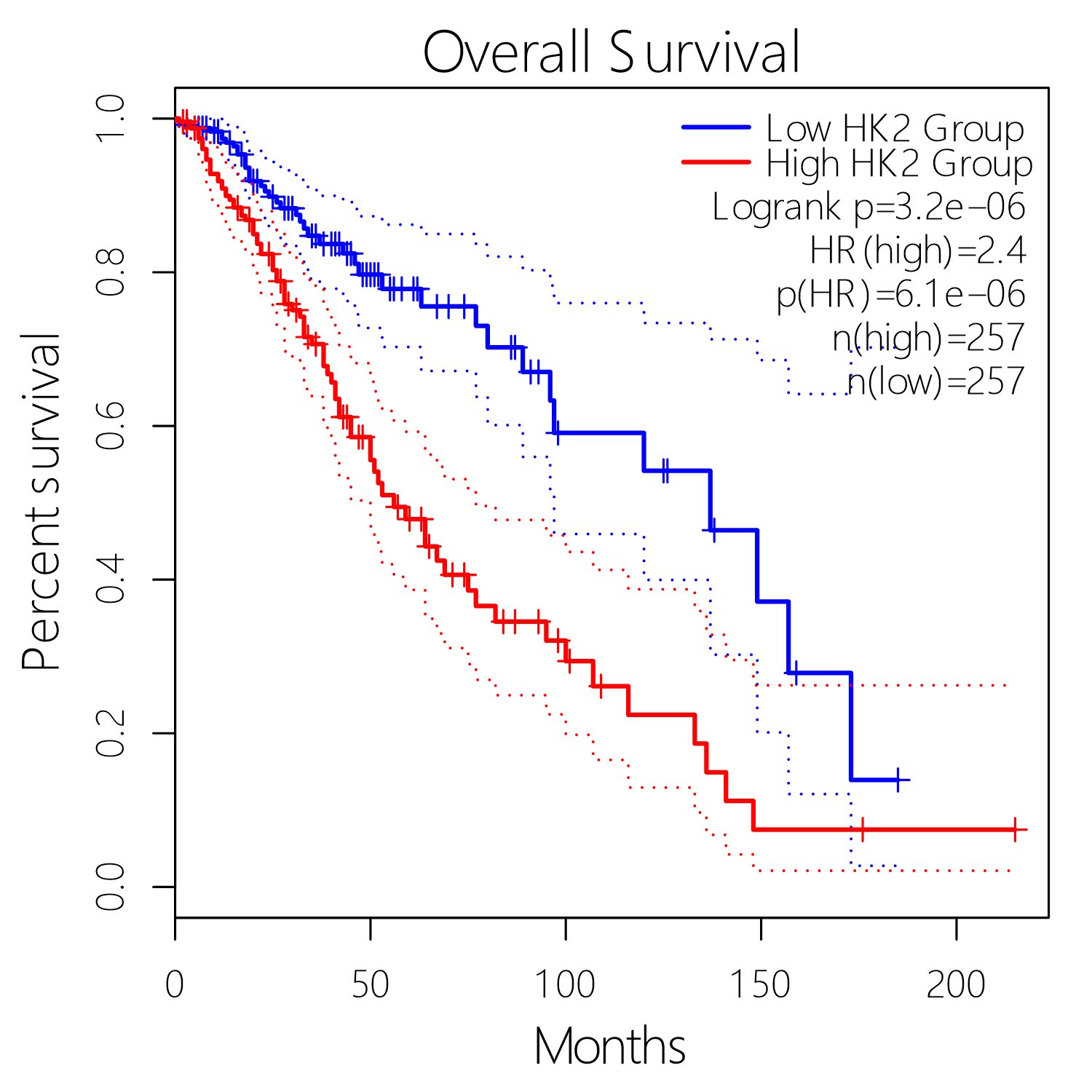


LIHC


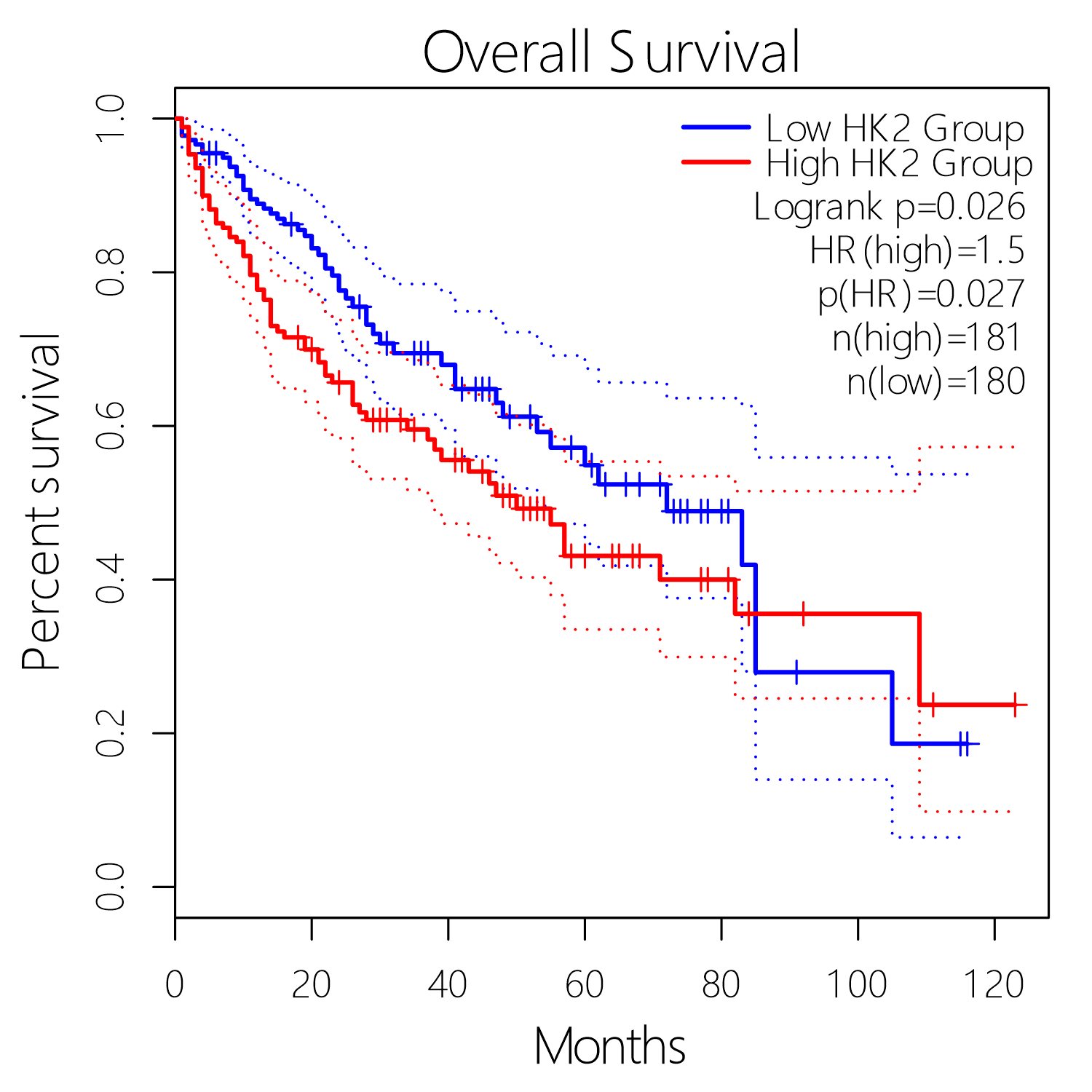


LUAD


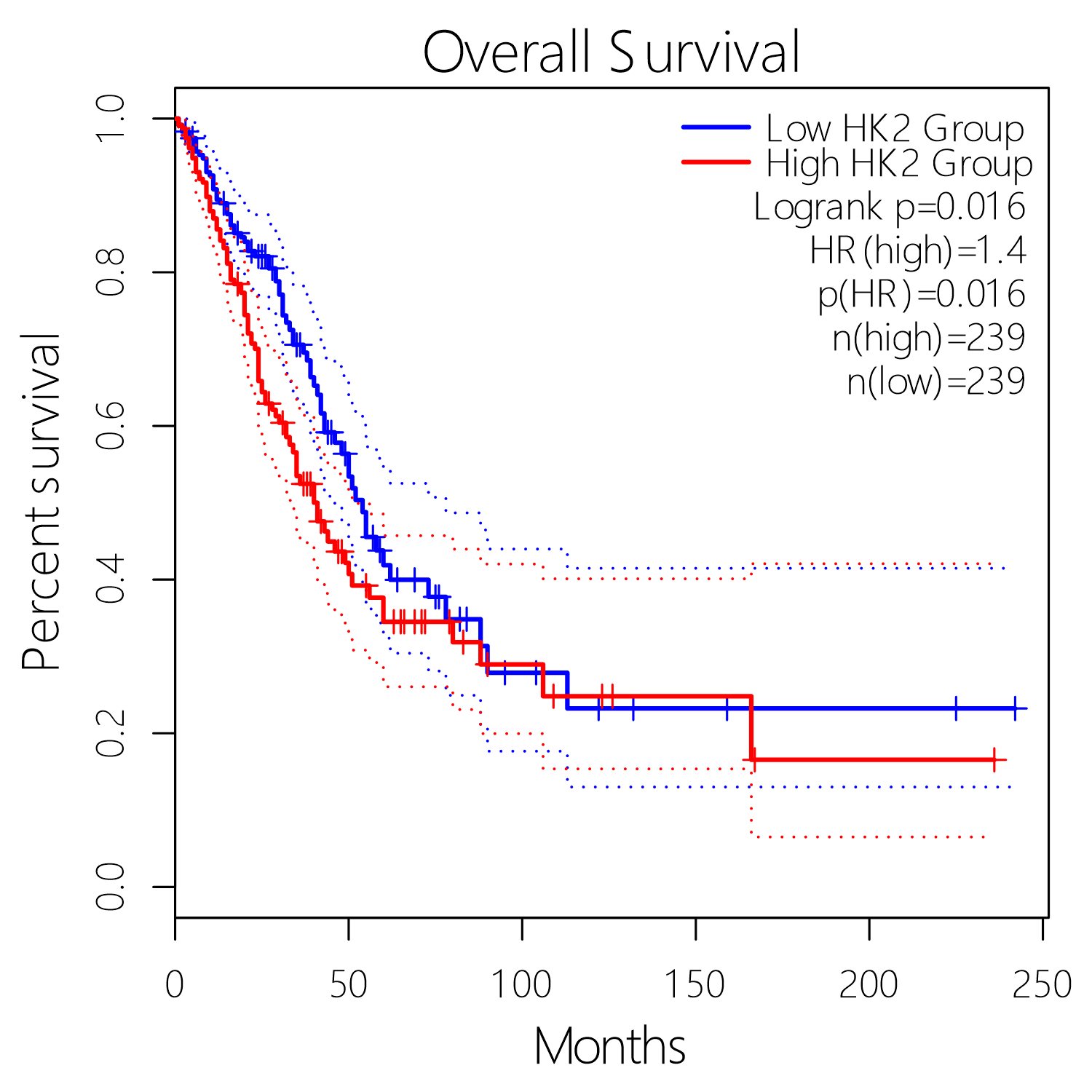


SARC


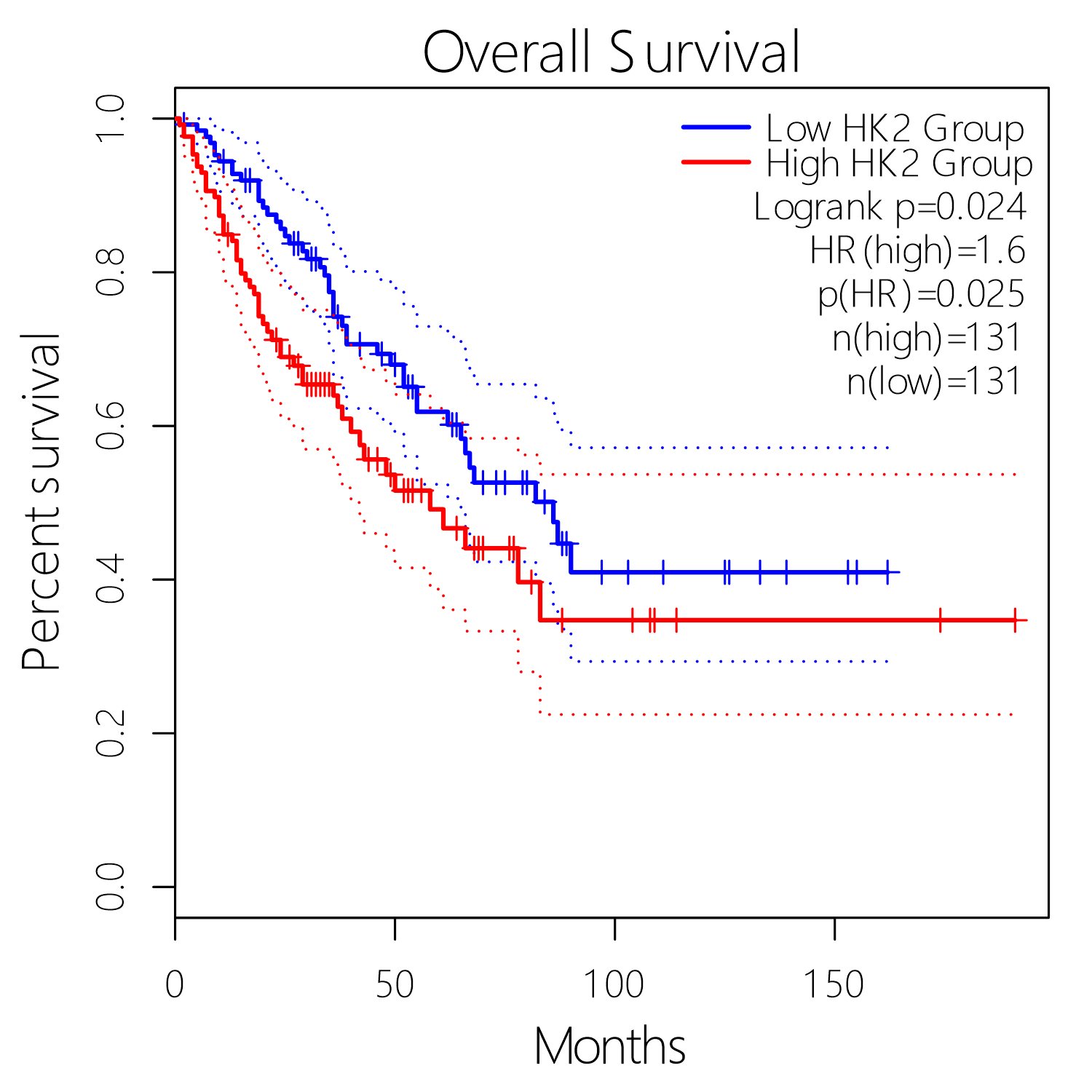


Fig 2b: Correlation between *HK2* gene expression and disease-free survival


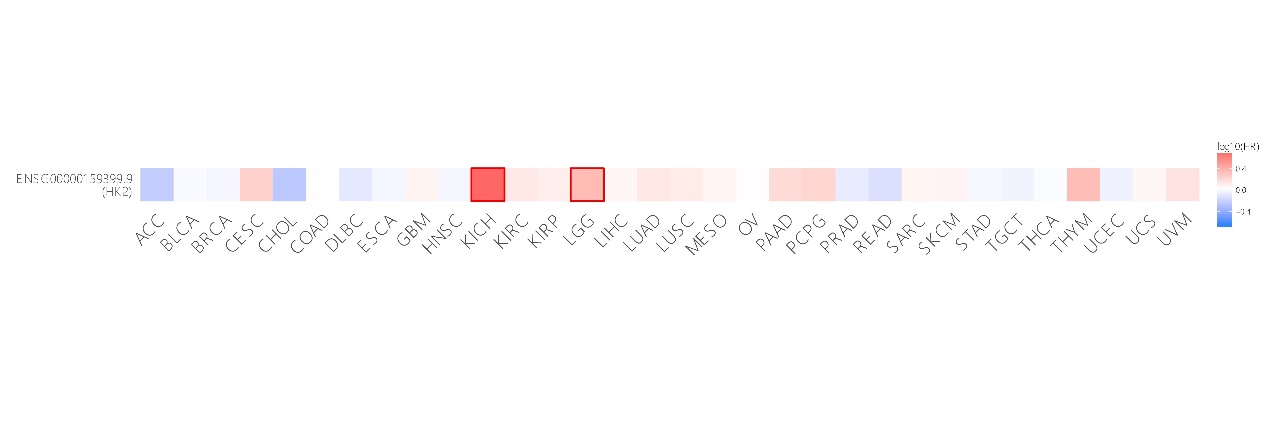
The survival map and Kaplan-Meier curves with positive results are given.

KICH


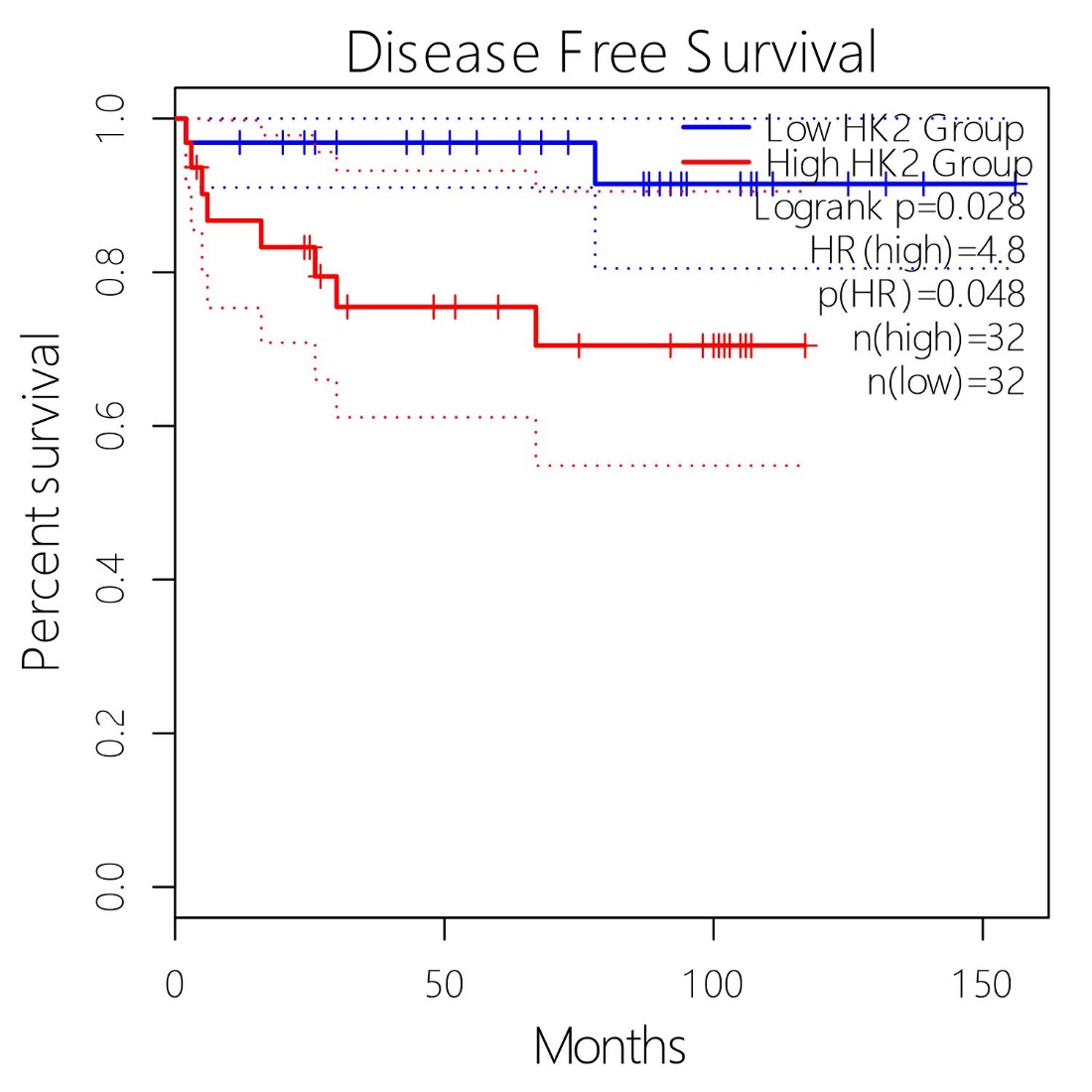


LGG


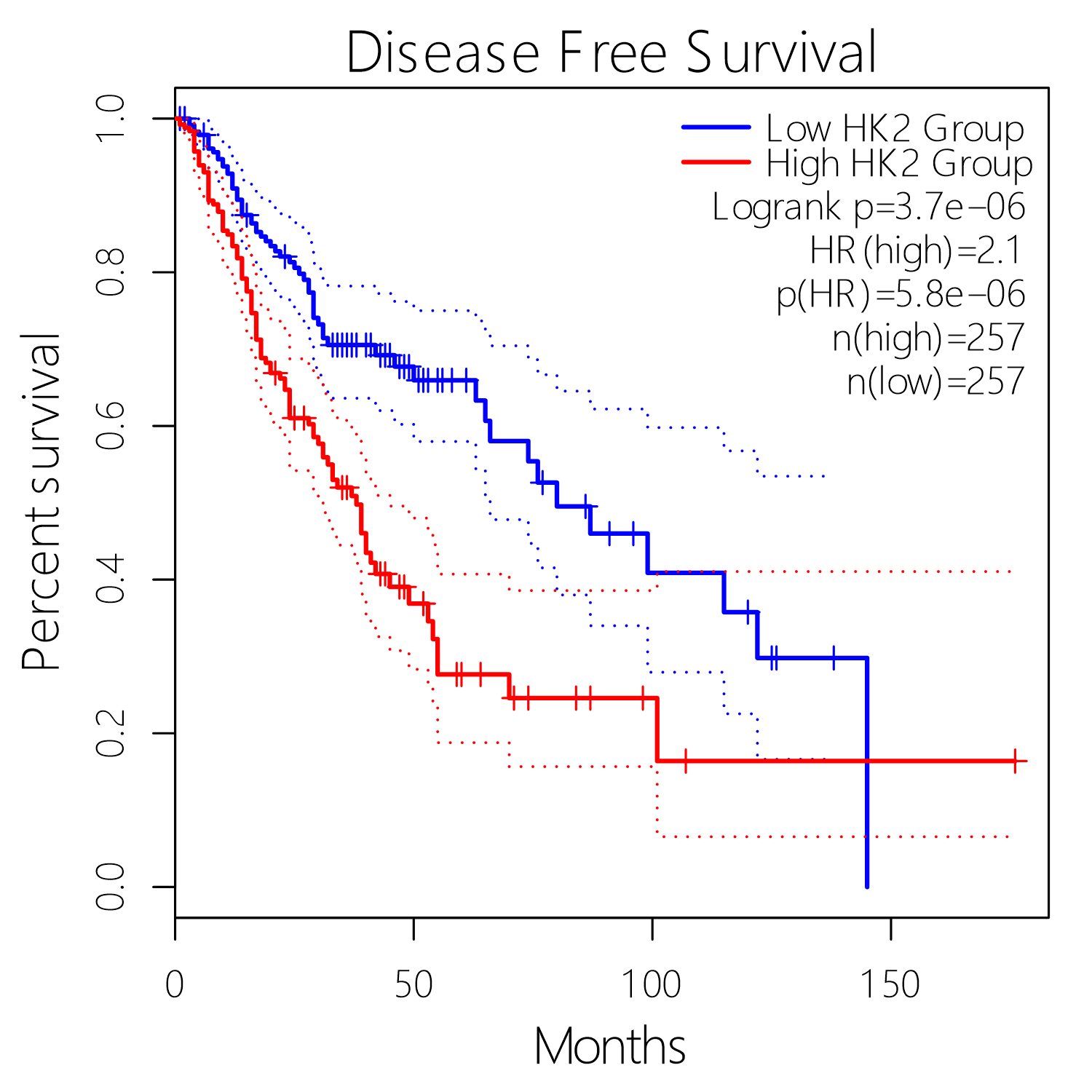

Supplement: Supplementary file 2 — Supplementary Information 2. [file 41598_2022_23598_MOESM2_ESM.docx]
