# Supplementary material for: A pan-cancer analysis of the role of hexokinase II (HK2) in human tumors
Source: Sci Rep. 2022 Nov 5;12:18807. doi: 10.1038/s41598-022-23598-8 (PMC9637150; doi:10.1038/s41598-022-23598-8)

Fig 3a: The alteration frequency with mutation type


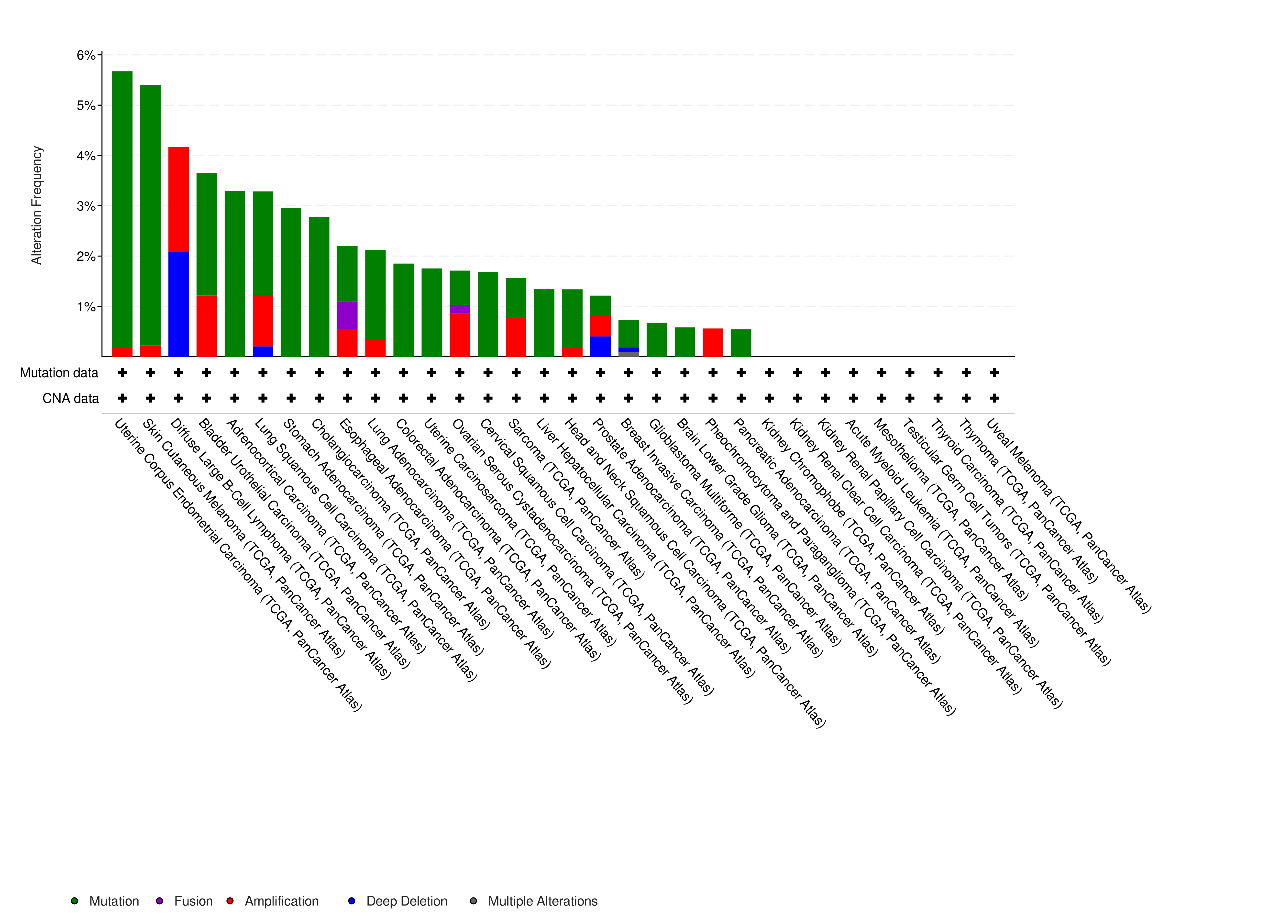


Fig 3b: The mutation site (V431Cfs*26, L795Rfs*10, and A901Rfs*74) in the 3D structure of HK2


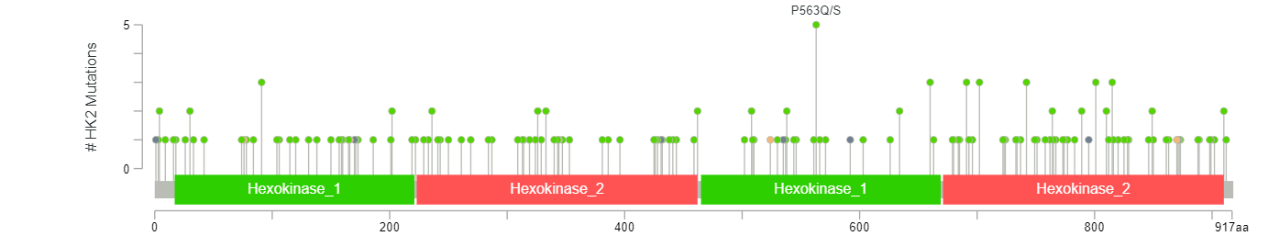


V431C
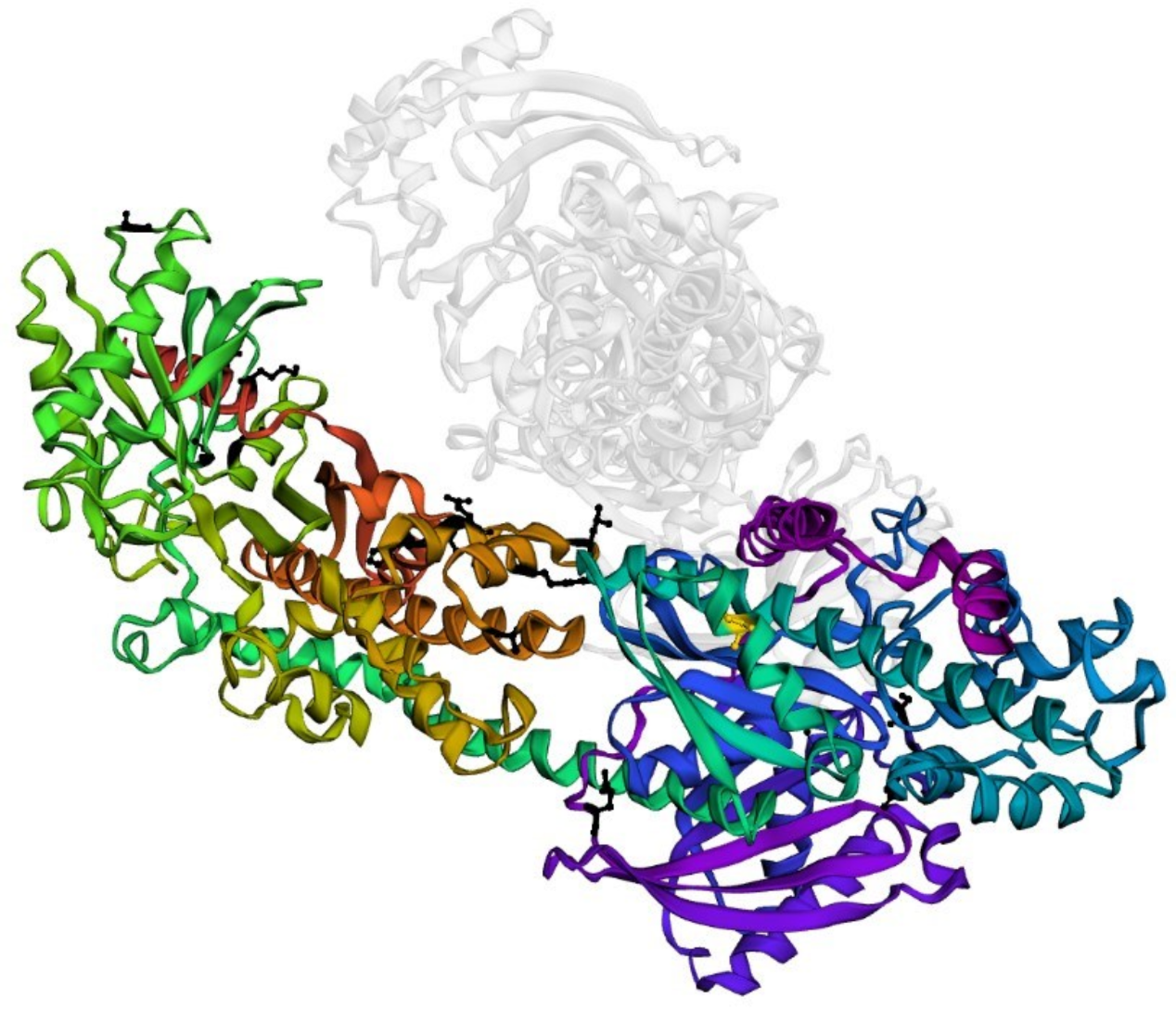


L795R
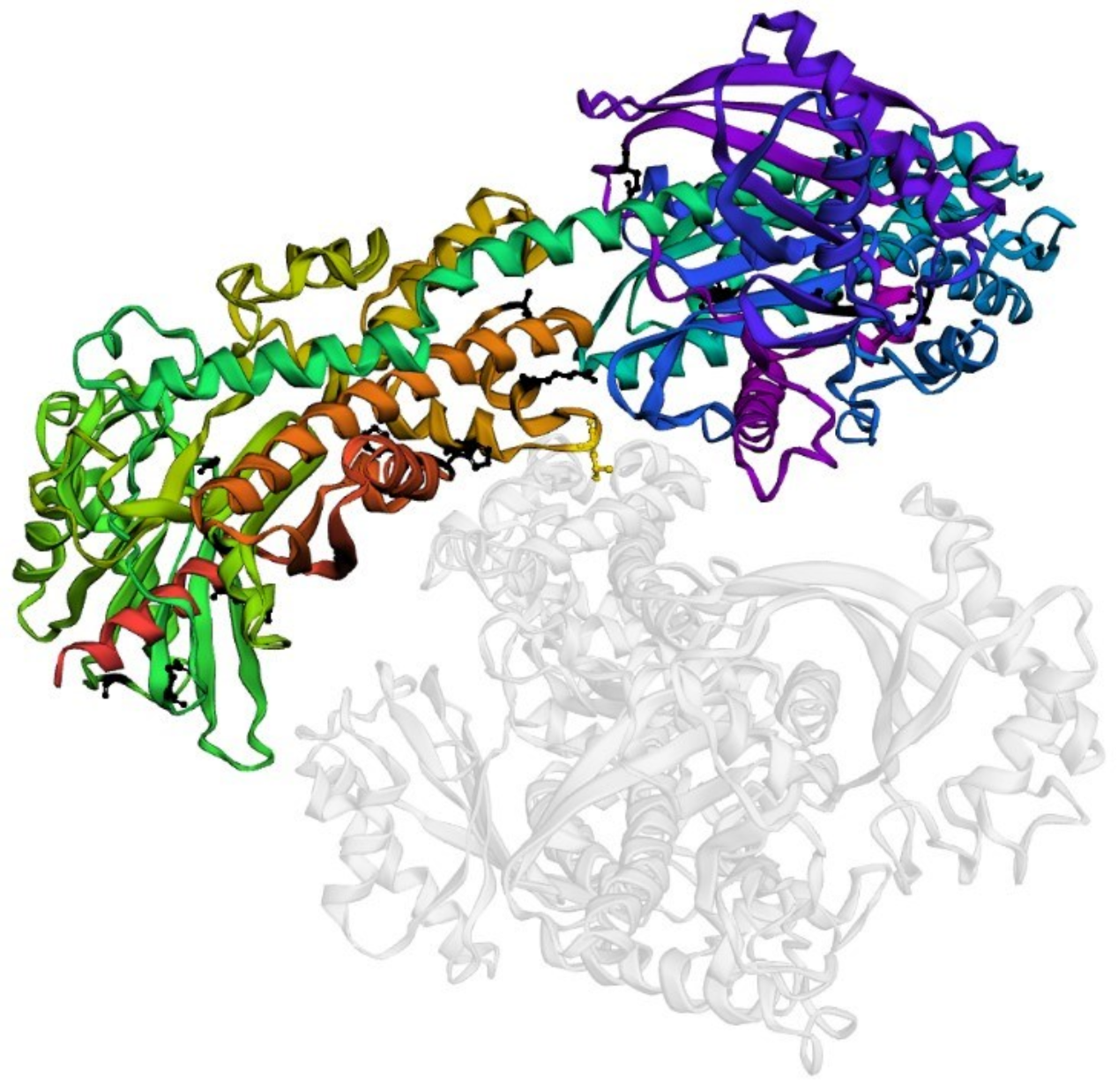


A901R


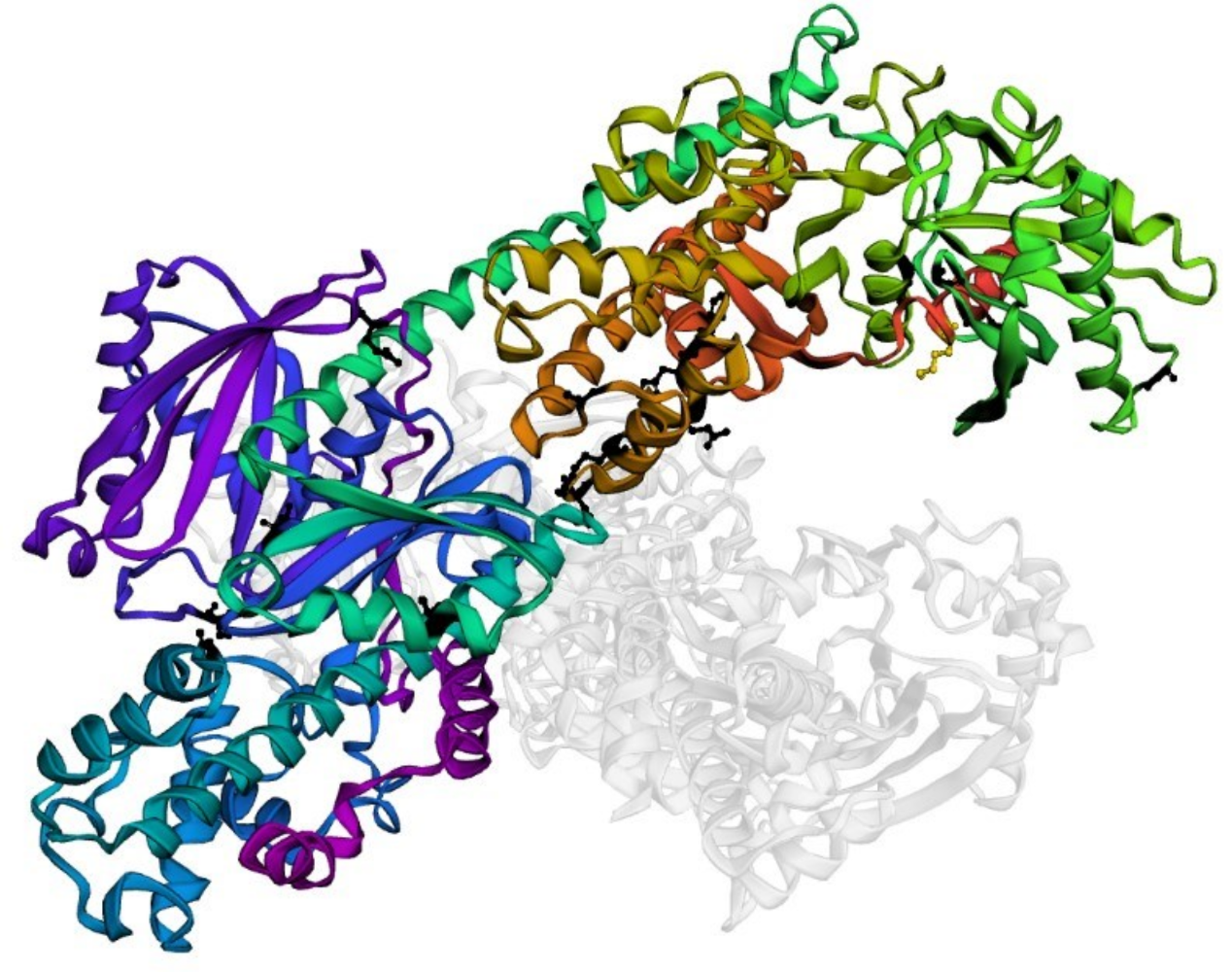


Fig 3c: The potential correlation between mutation status and overall, disease-specific, disease-free, and progression-free survival of UCEC.

Overall survival


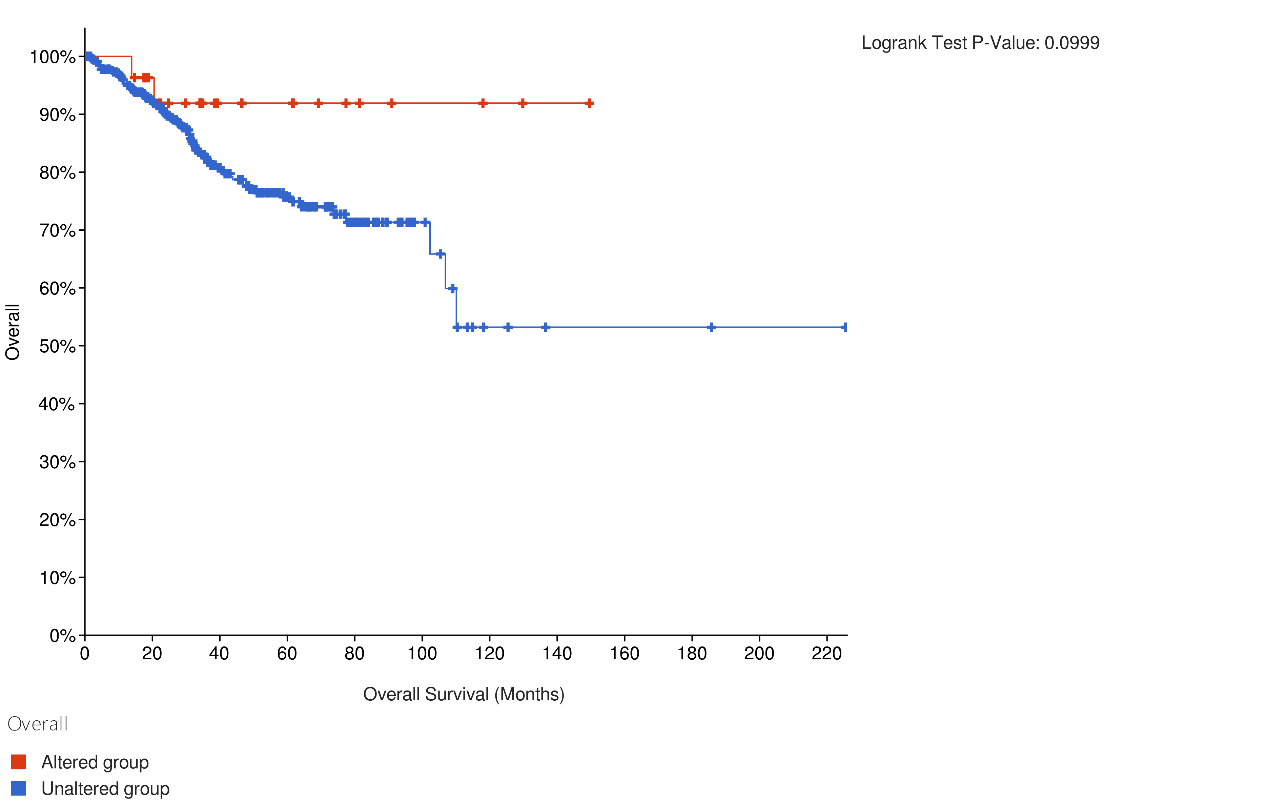


Disease-specific survival


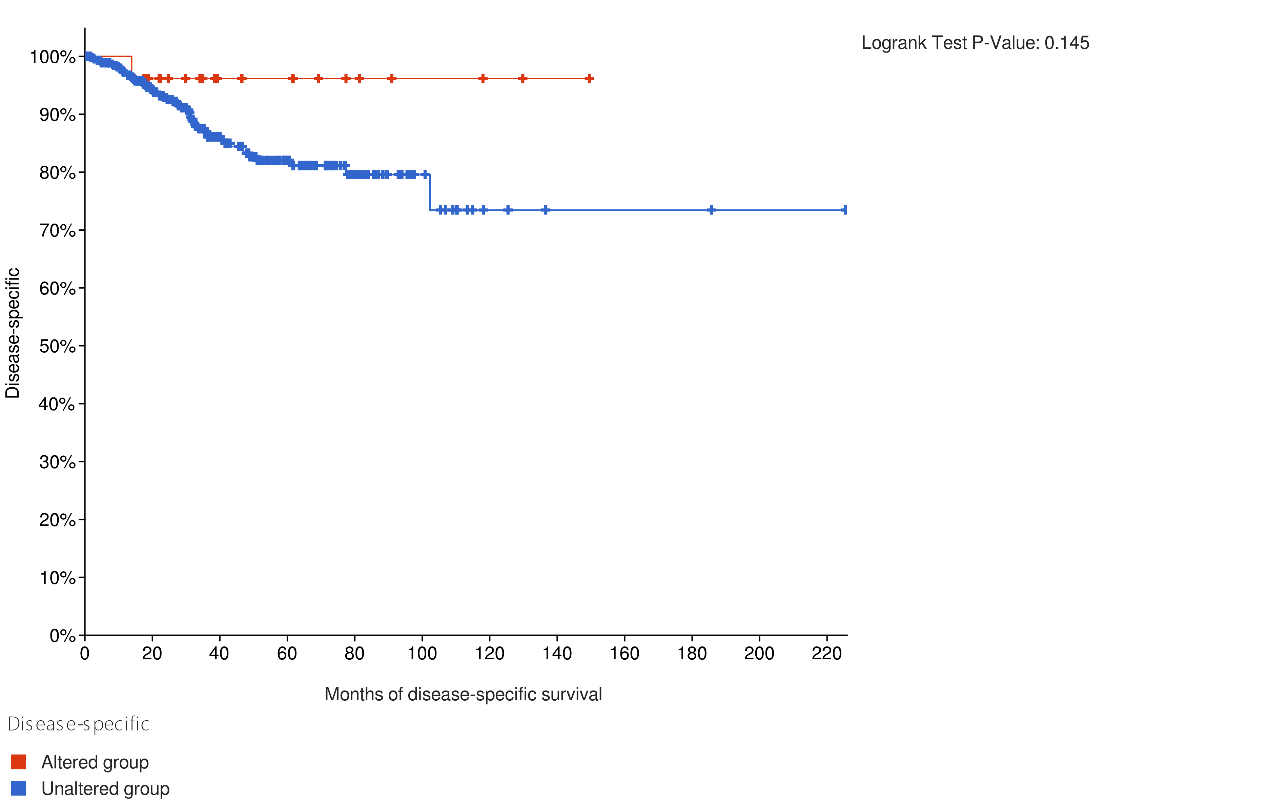


Disease-free survival


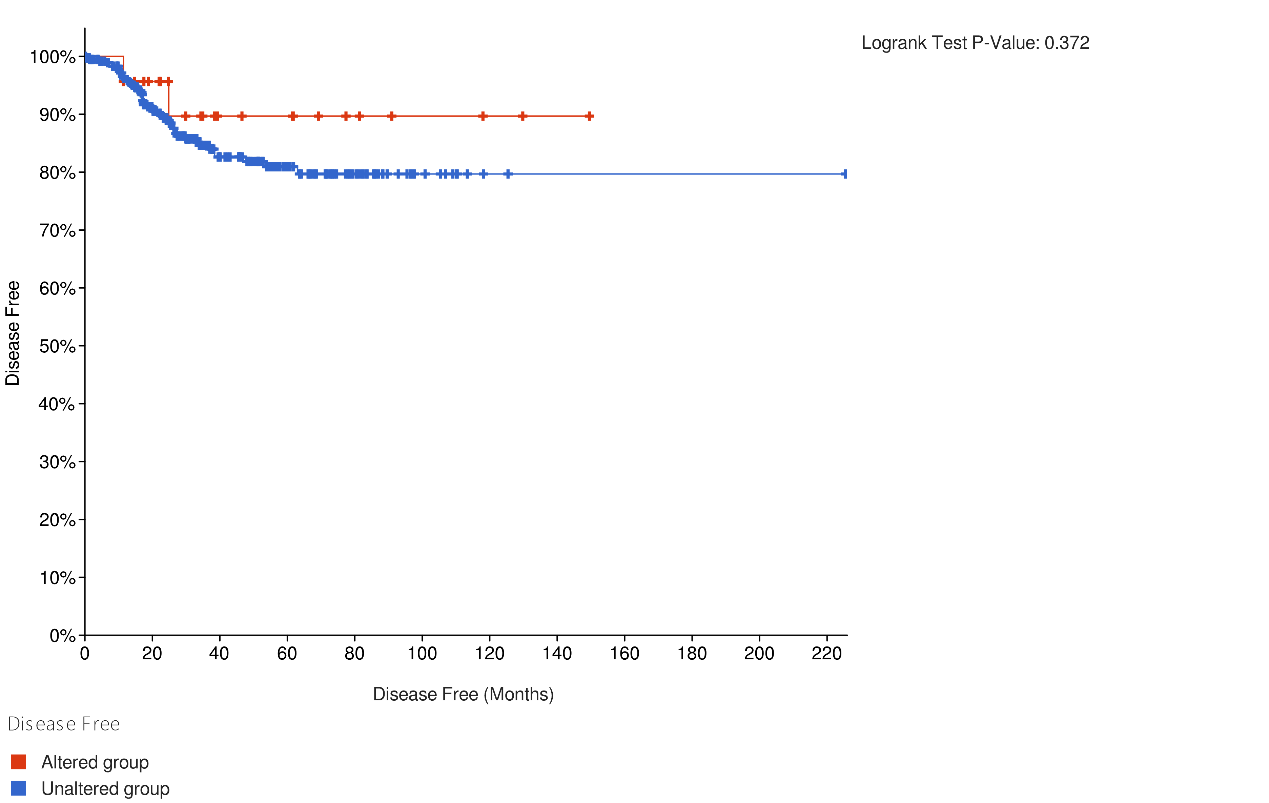


Progression-free survival


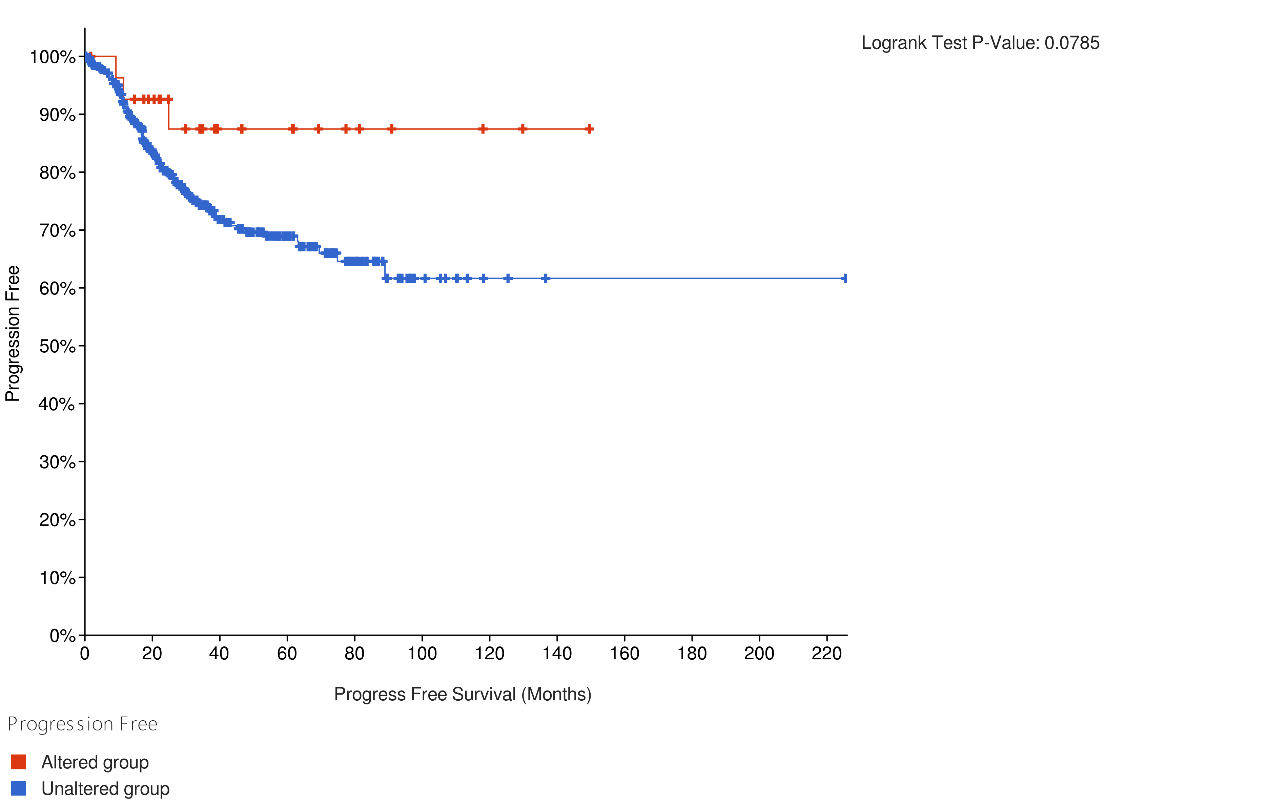

Supplement: Supplementary file 3 — Supplementary Information 3. [file 41598_2022_23598_MOESM3_ESM.docx]
