# Supplementary material for: A pan-cancer analysis of the role of hexokinase II (HK2) in human tumors
Source: Sci Rep. 2022 Nov 5;12:18807. doi: 10.1038/s41598-022-23598-8 (PMC9637150; doi:10.1038/s41598-022-23598-8)

Fig 4a: Different algorithms were used to explore the potential correlation between the expression level of the HK2 gene and the infiltration level of cancer-associated fibroblasts


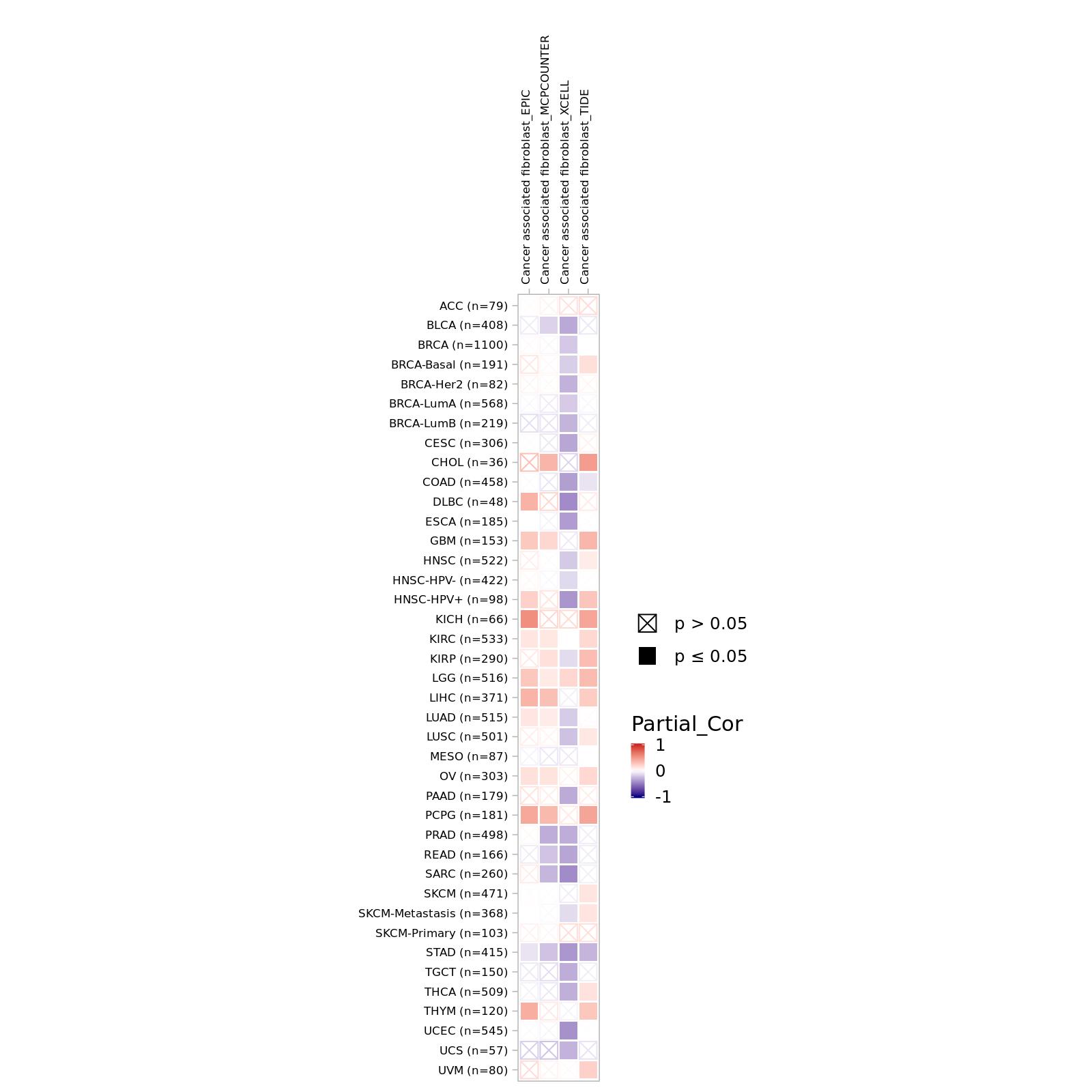


Fig 4b: Correlation between the expression level of the HK2 gene and the infiltration level of cancer-associated fibroblasts of LGG and STAD.

LGG


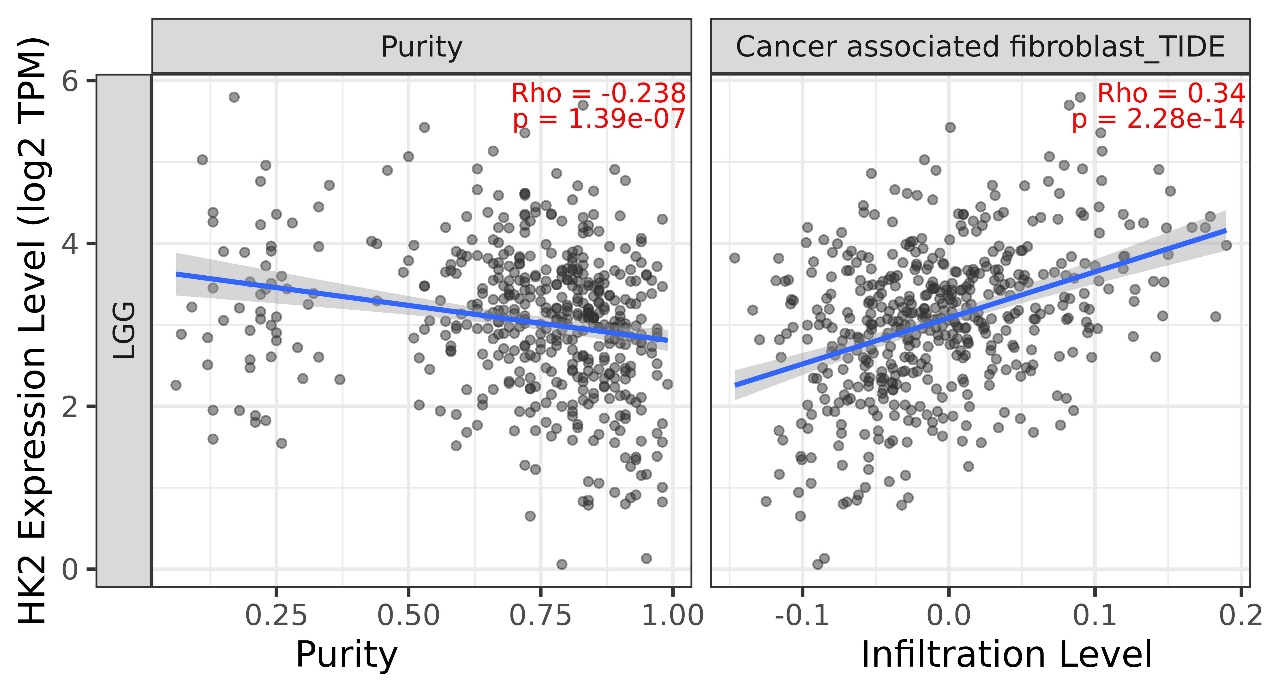


STAD


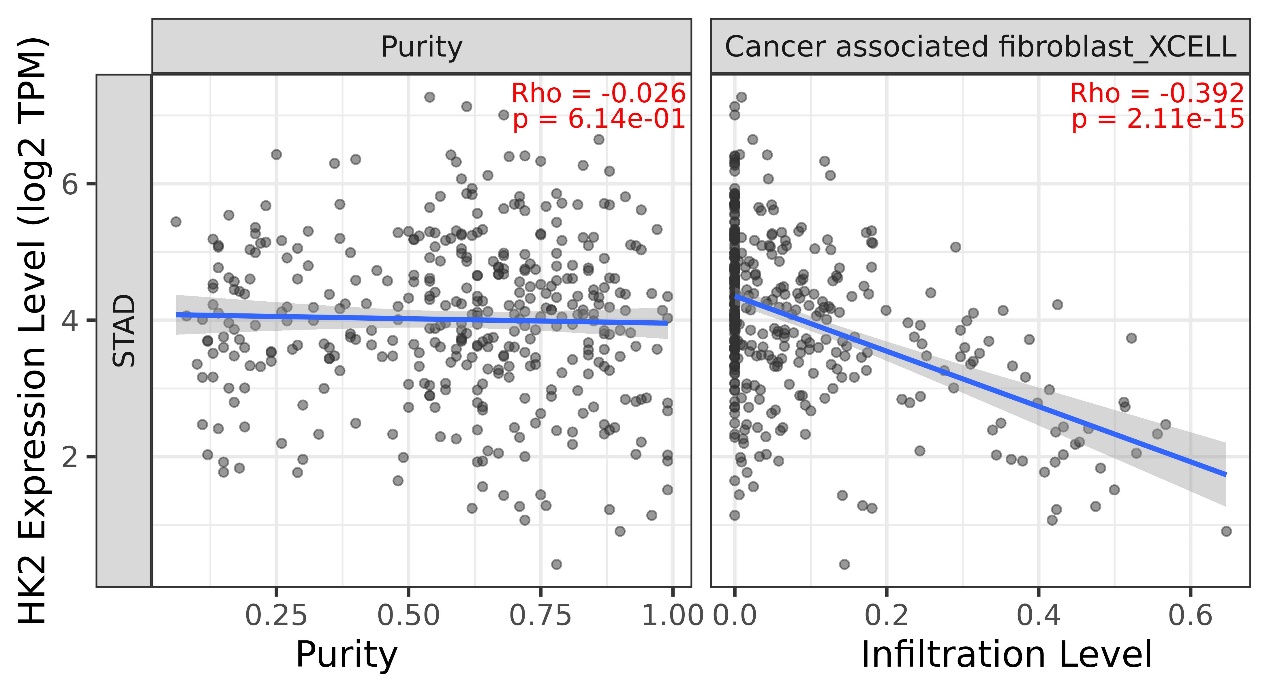


Fig 4c: Different algorithms were used to explore the potential correlation between the expression level of the HK2 gene and the infiltration level of T follicular helper cells.


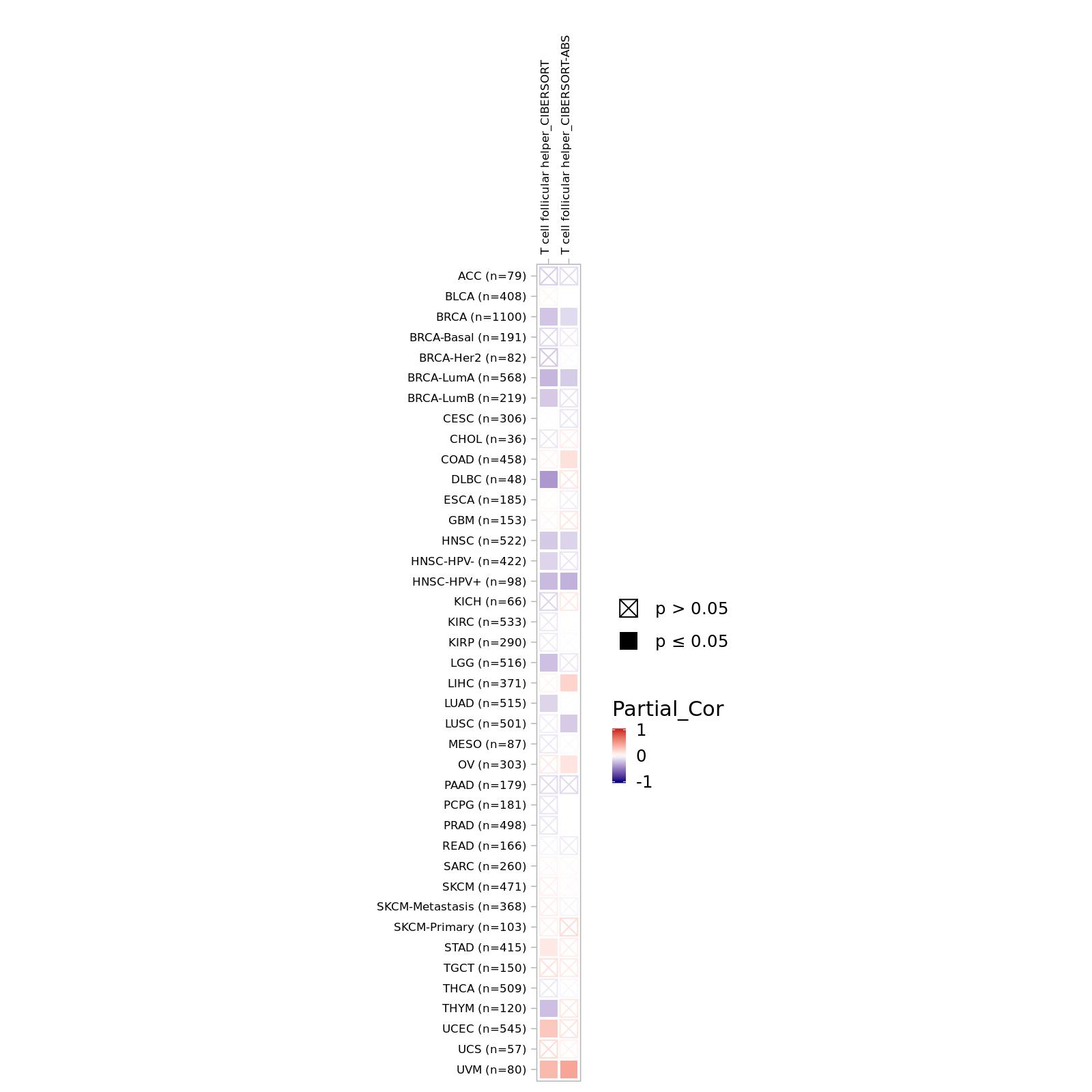


Fig 4d: Correlation between the expression level of the HK2 gene and the infiltration level of T follicular helper cells of BRCA, BRCA-LumA, HNSC, HNSC-HPV+, UVM.

BRCA


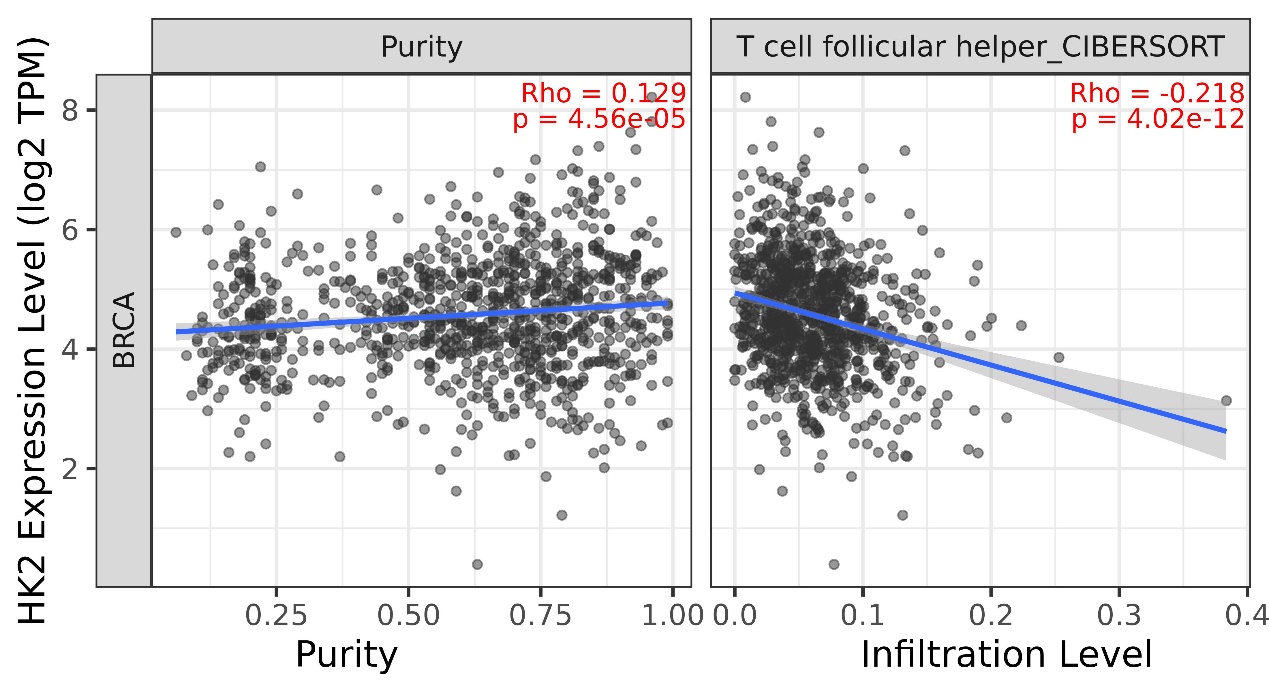


BRCA-LumA


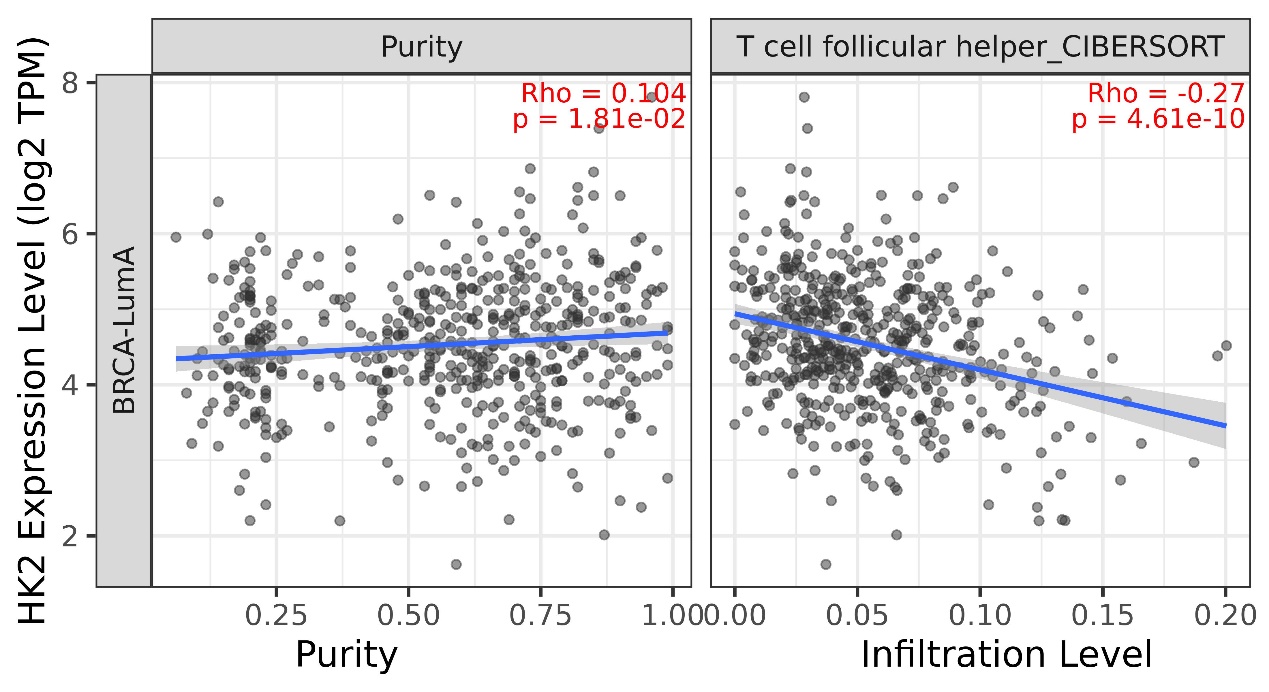


HNSC


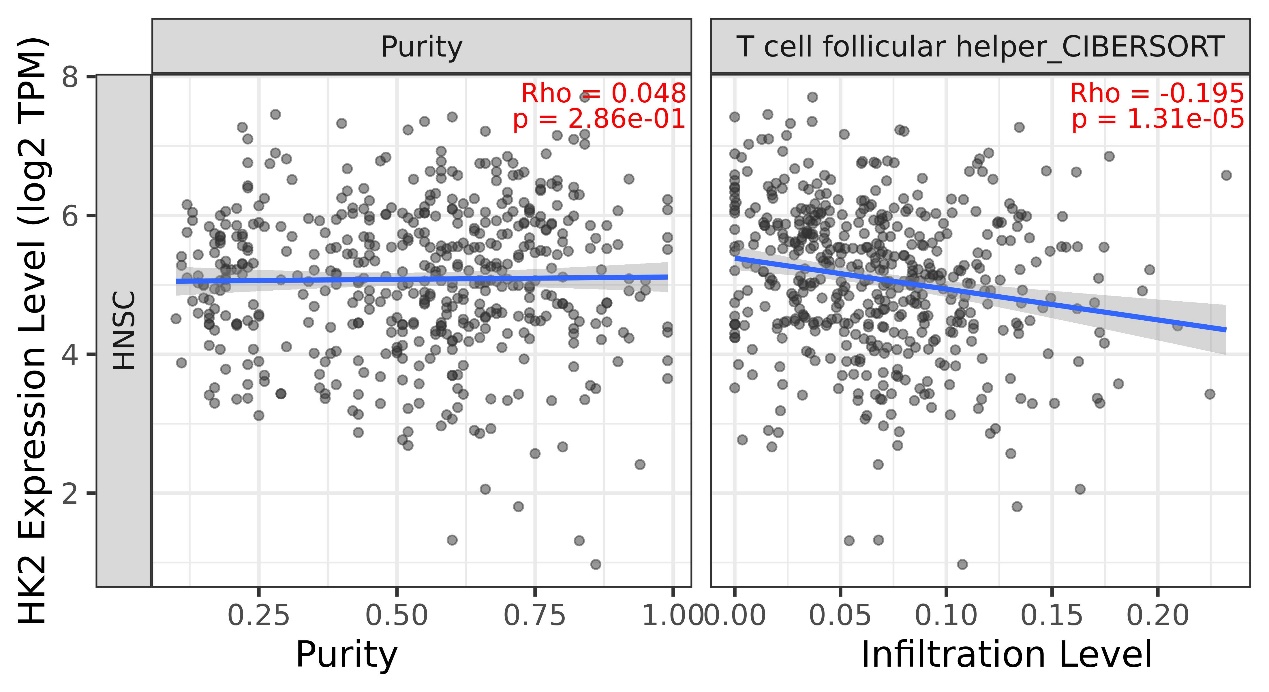


HNSC-HPV+


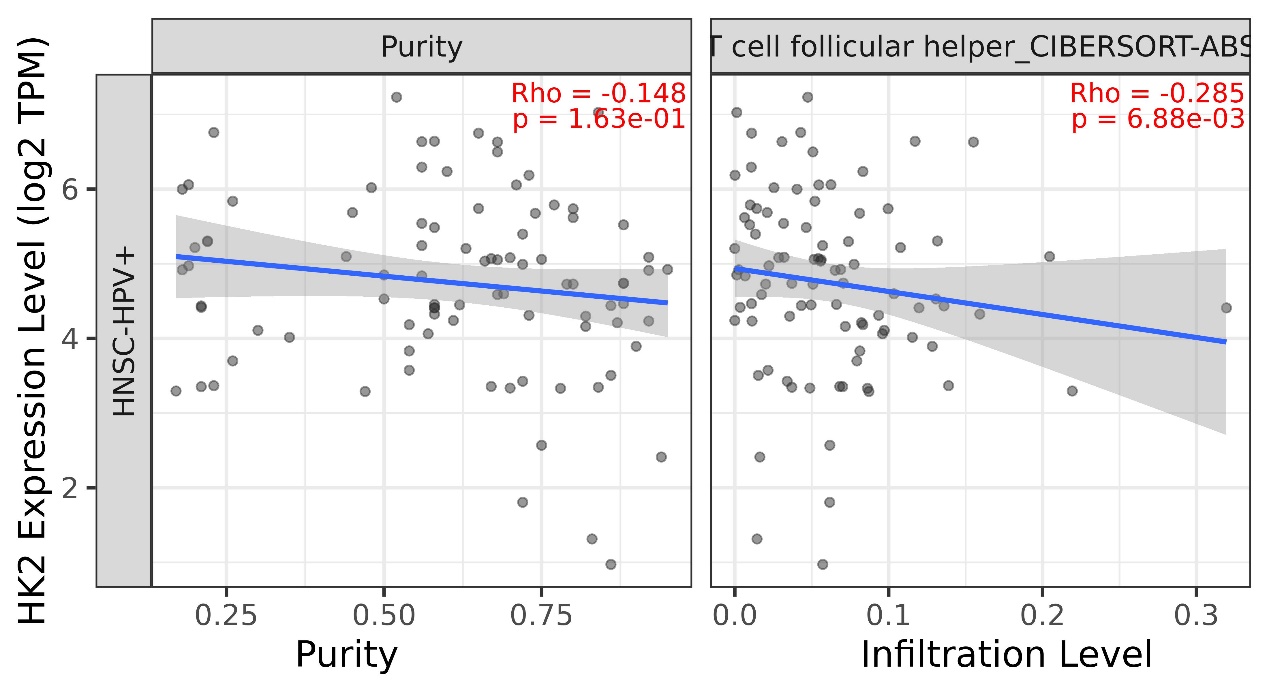


UVM


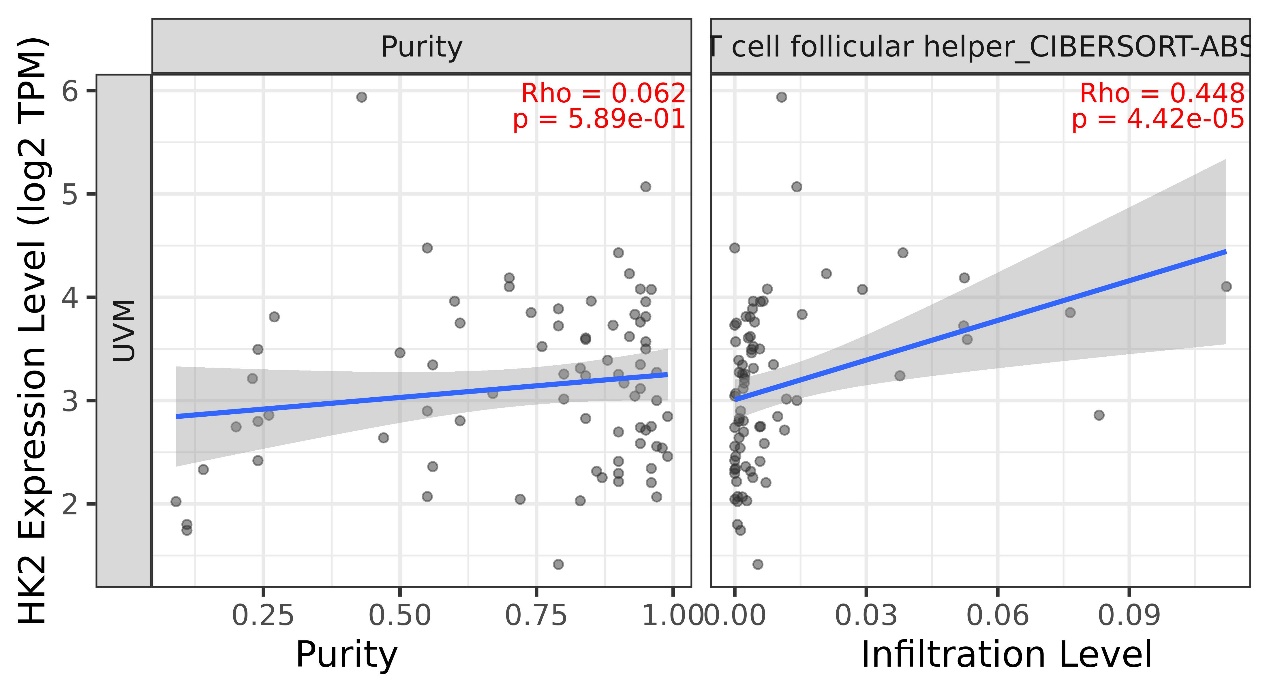

Supplement: Supplementary file 4 — Supplementary Information 4. [file 41598_2022_23598_MOESM4_ESM.docx]
