# Supplementary material for: A pan-cancer analysis of the role of hexokinase II (HK2) in human tumors
Source: Sci Rep. 2022 Nov 5;12:18807. doi: 10.1038/s41598-022-23598-8 (PMC9637150; doi:10.1038/s41598-022-23598-8)

Fig 5a: The STRING was used to obtain the available experimentally determined HK2-binding proteins


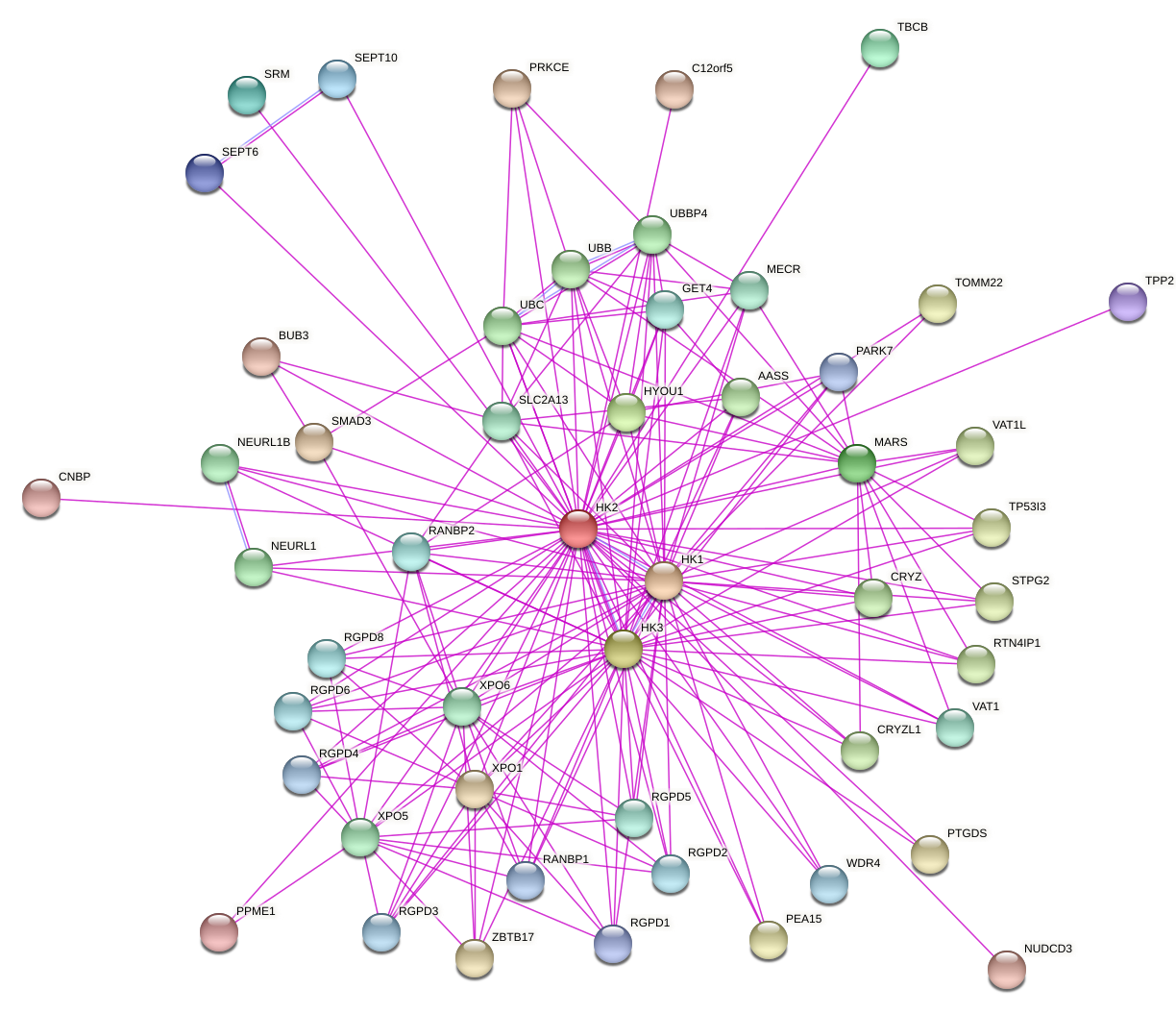


Fig 5b: Using the GEPIA2 approach, the top 10 HK2-correlated genes in TCGA projects and analyzed the expression correlation between HK2 and ACTR3, BZW1, CPSF2, GSK3B, GSPT1, KCMF1, MAPK6, NAA50, PGK1, and PSMD12.

ACTR3


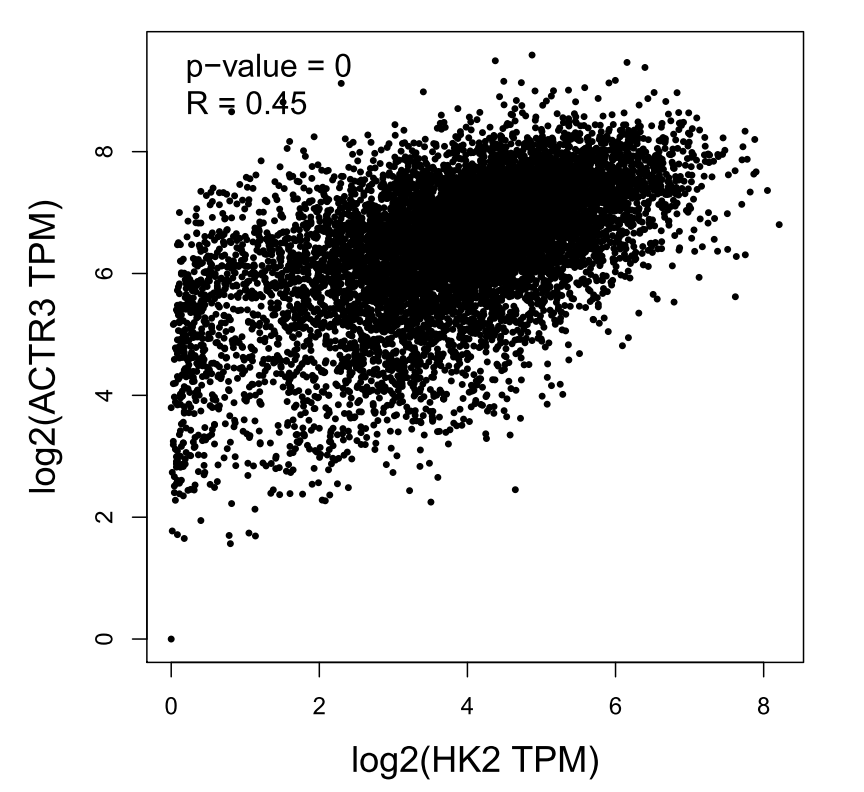


BZW1


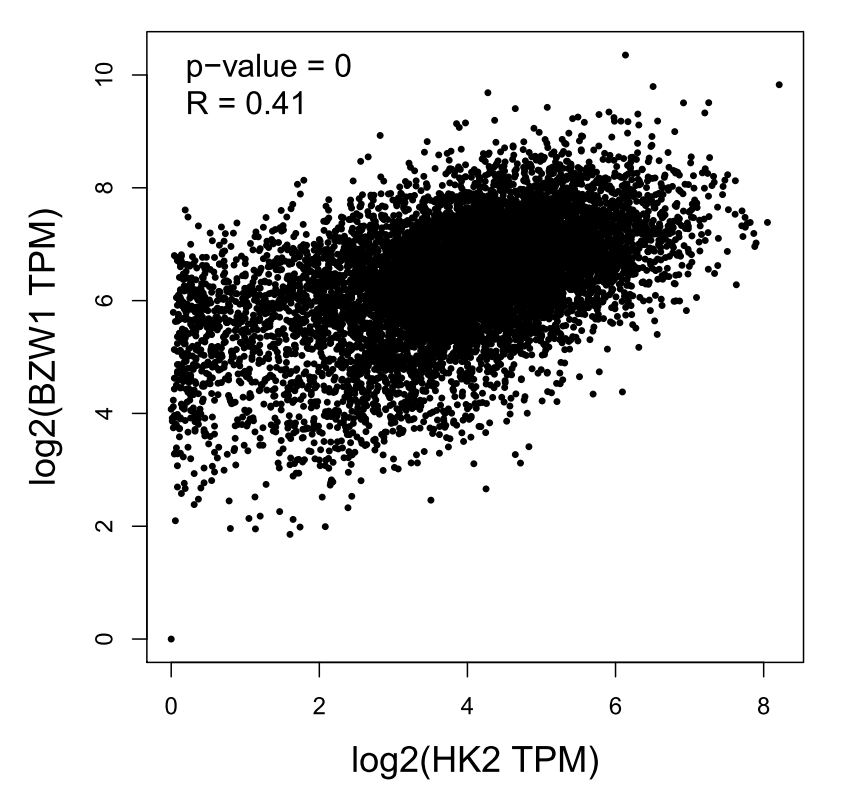


CPSF2


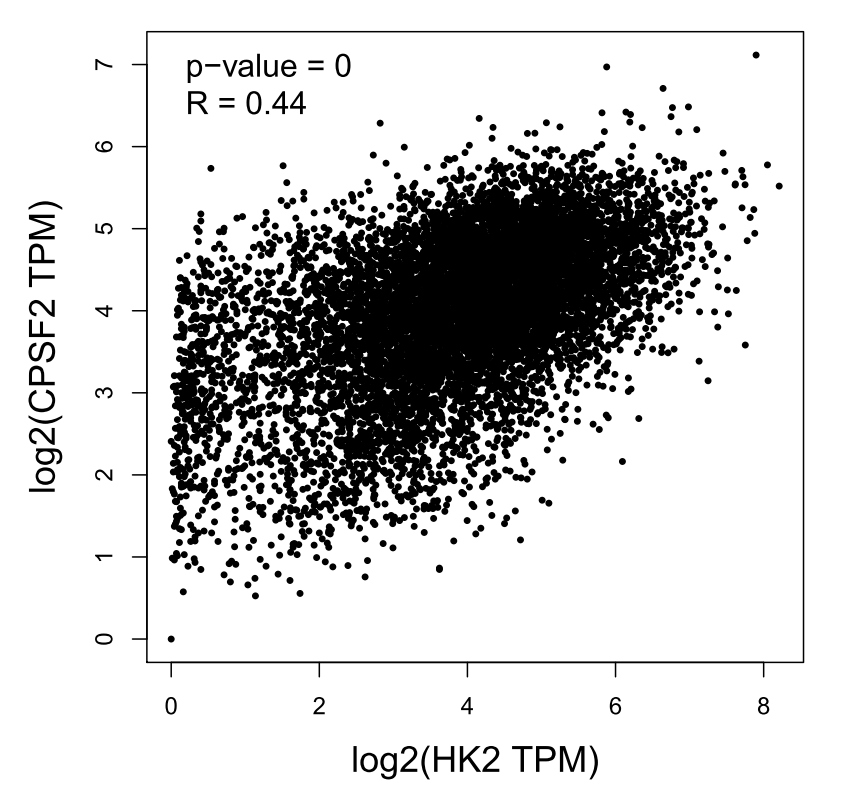


GSK3B


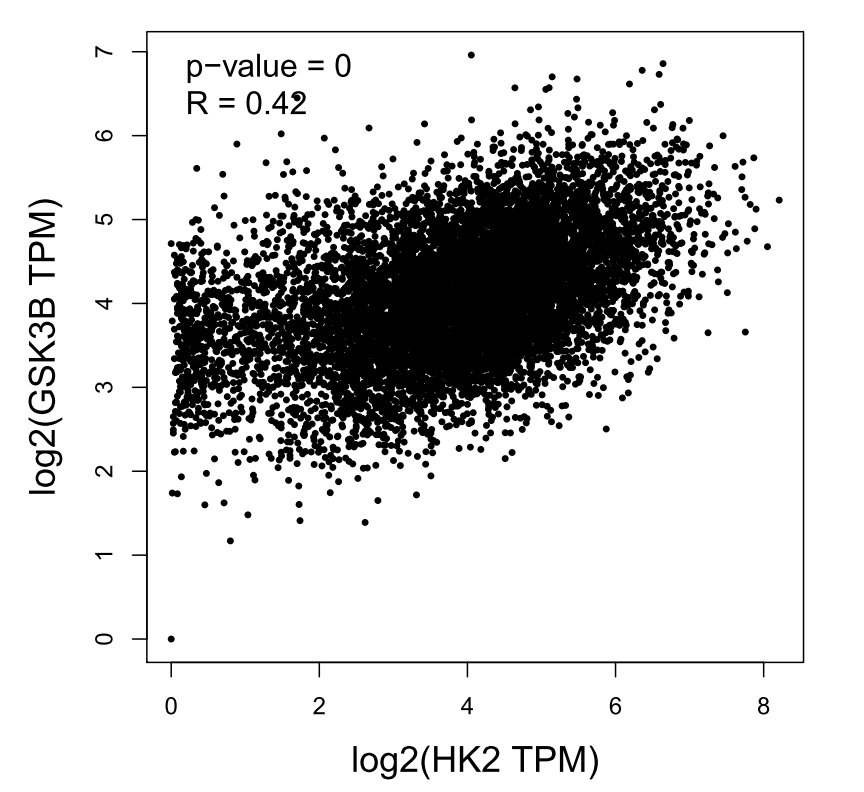


GSPT1


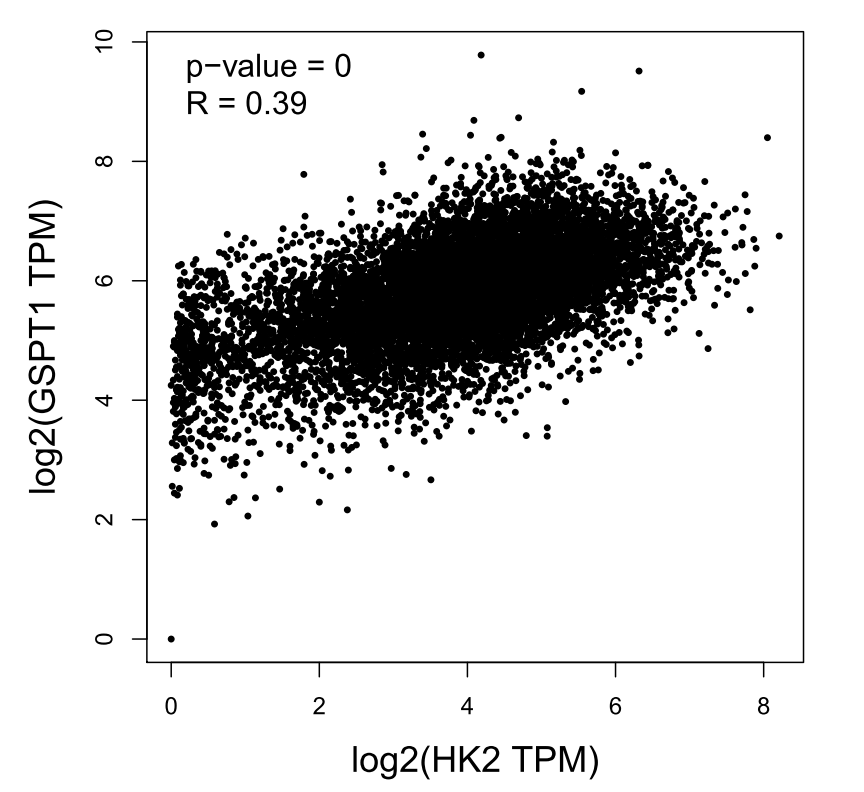


KCMF1


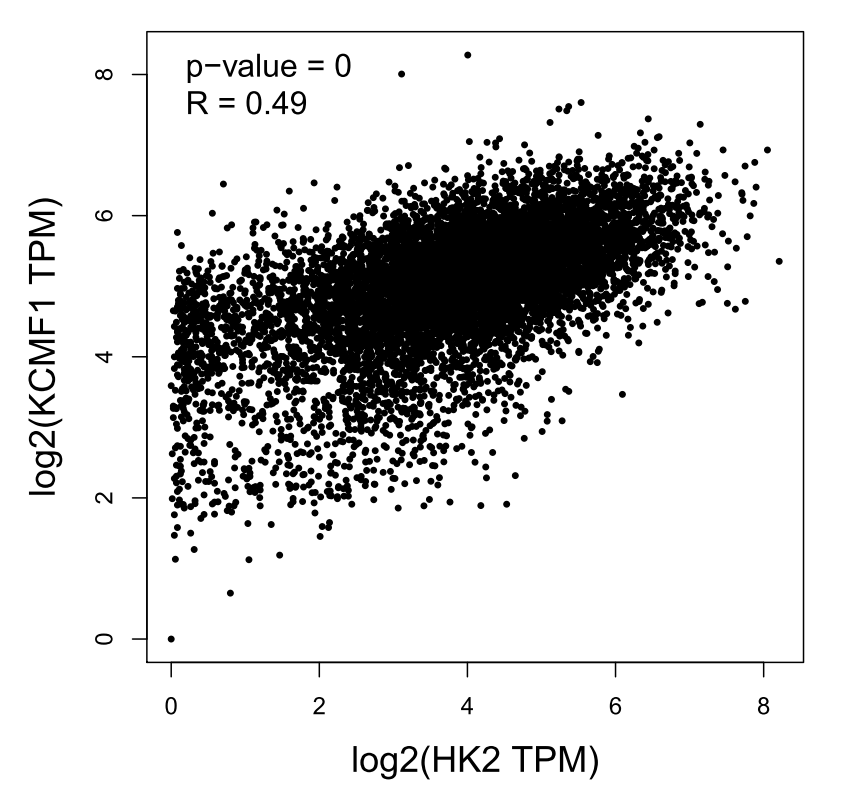


MAPK6


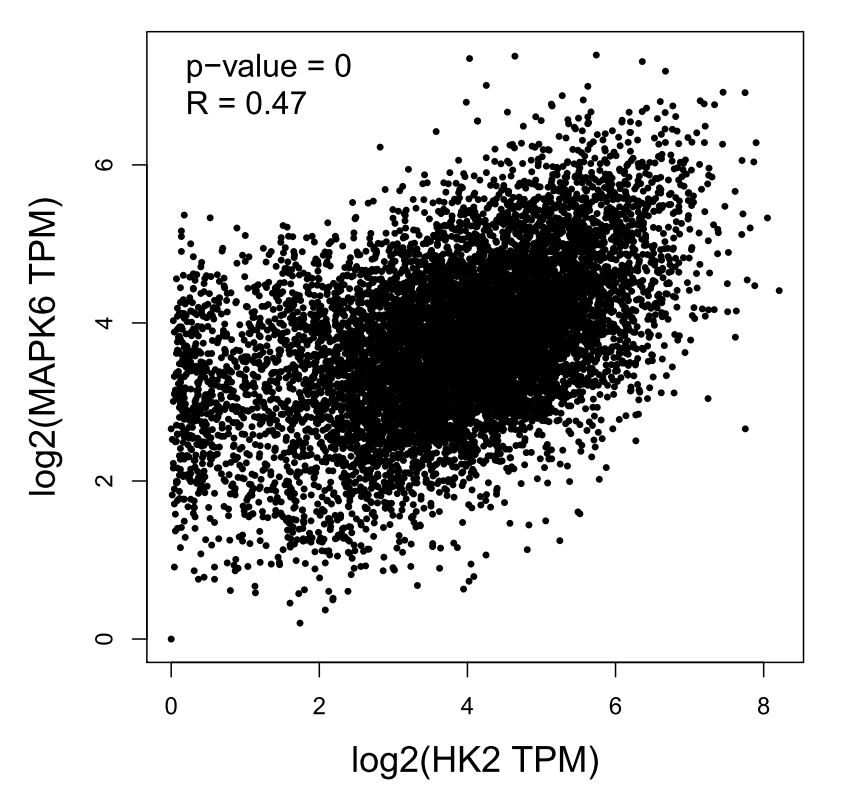


NAA50


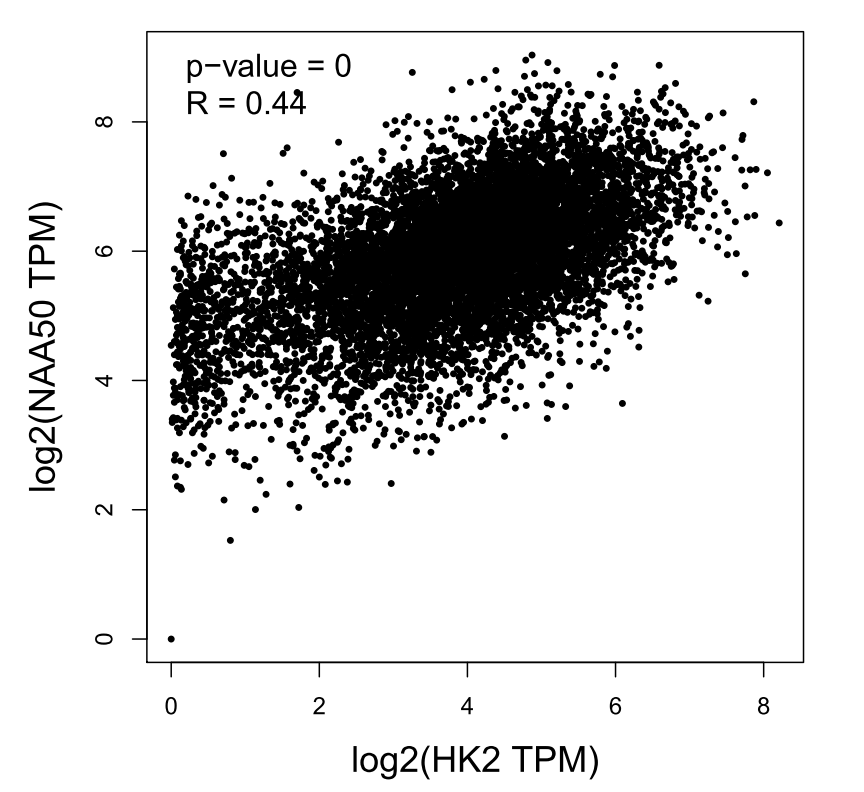


PGK1


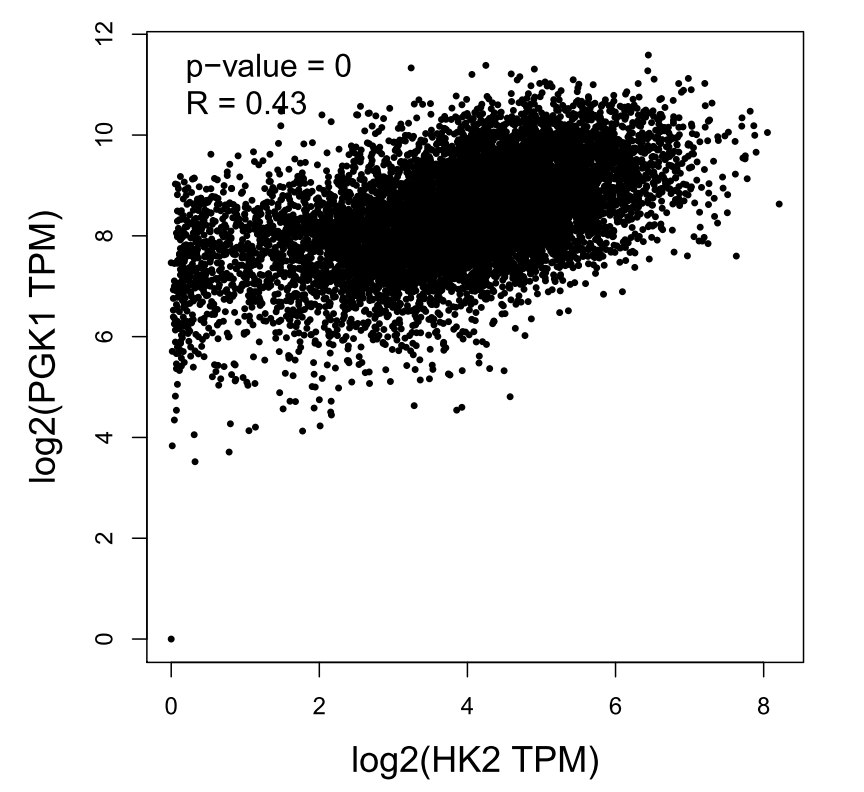


PSMD12


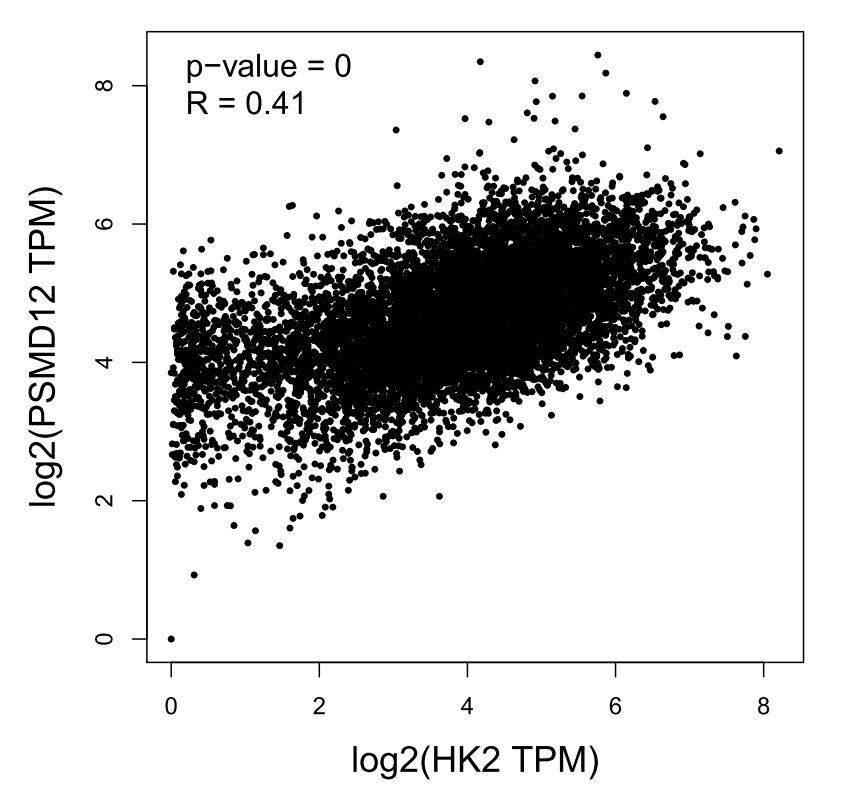


Fig 5c: The corresponding heatmap data in the detailed cancer types


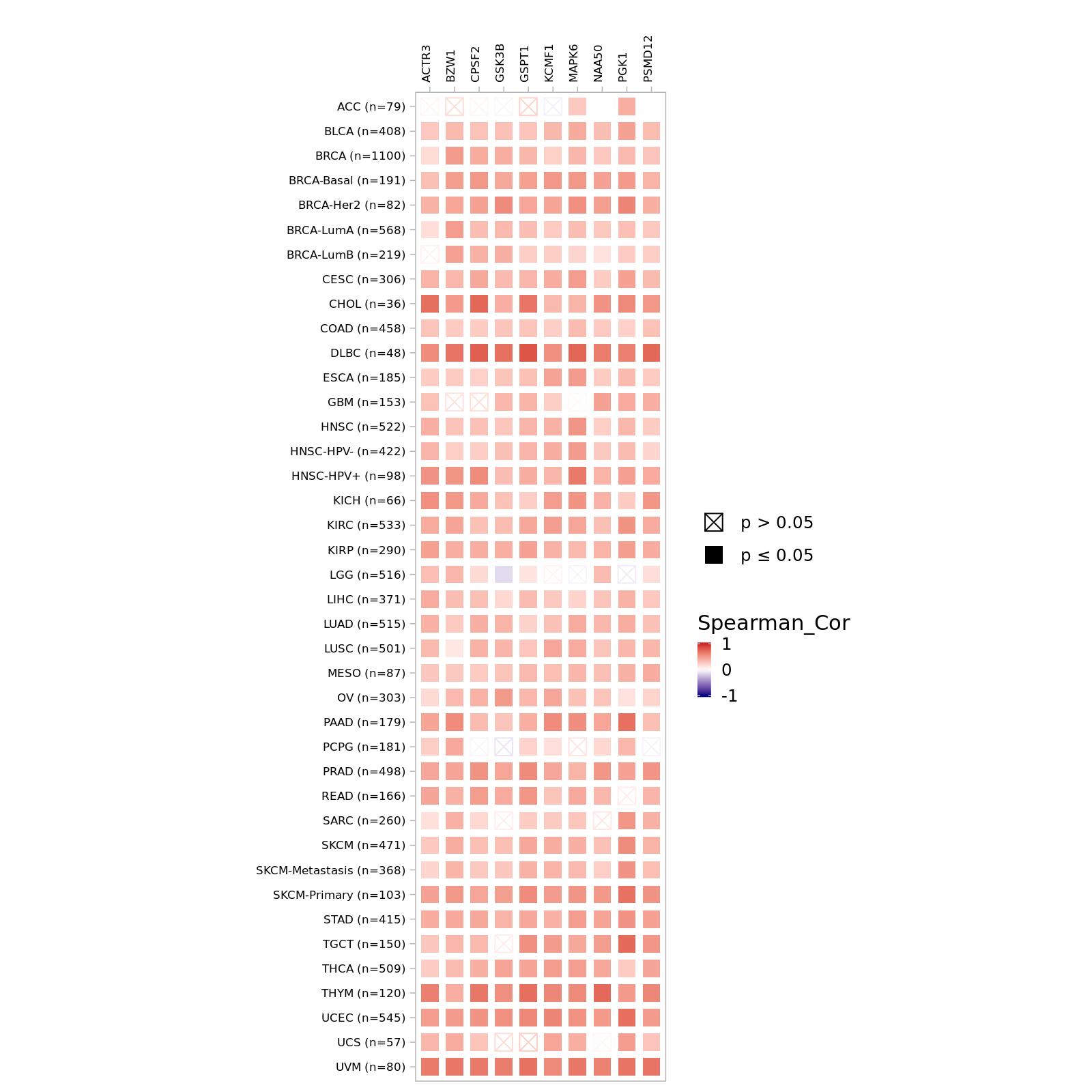


Fig 5d: An intersection analysis of the HK2-binding and correlated genes


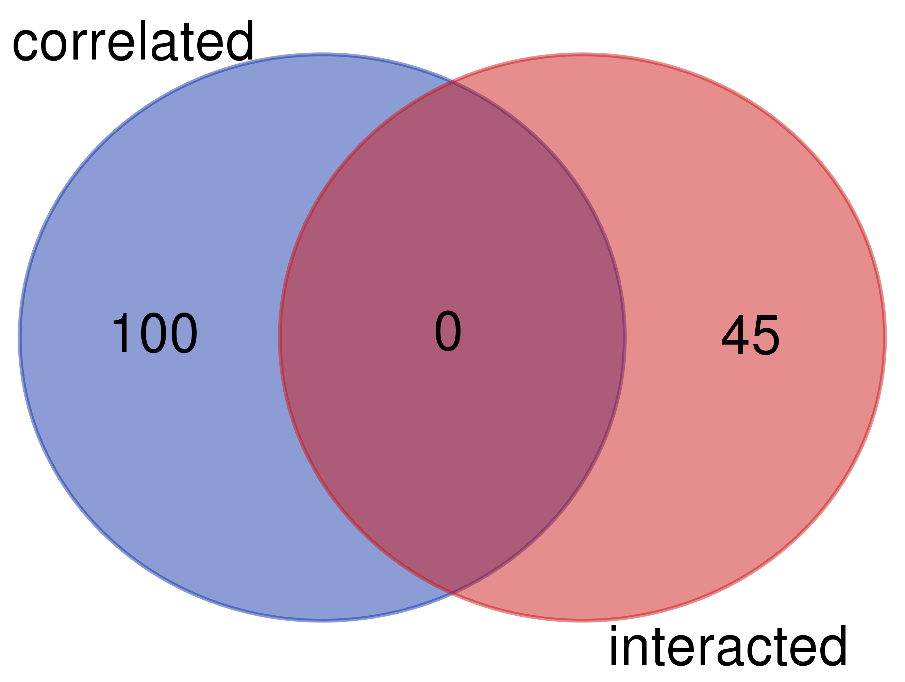


Fig 5e: KEGG pathway


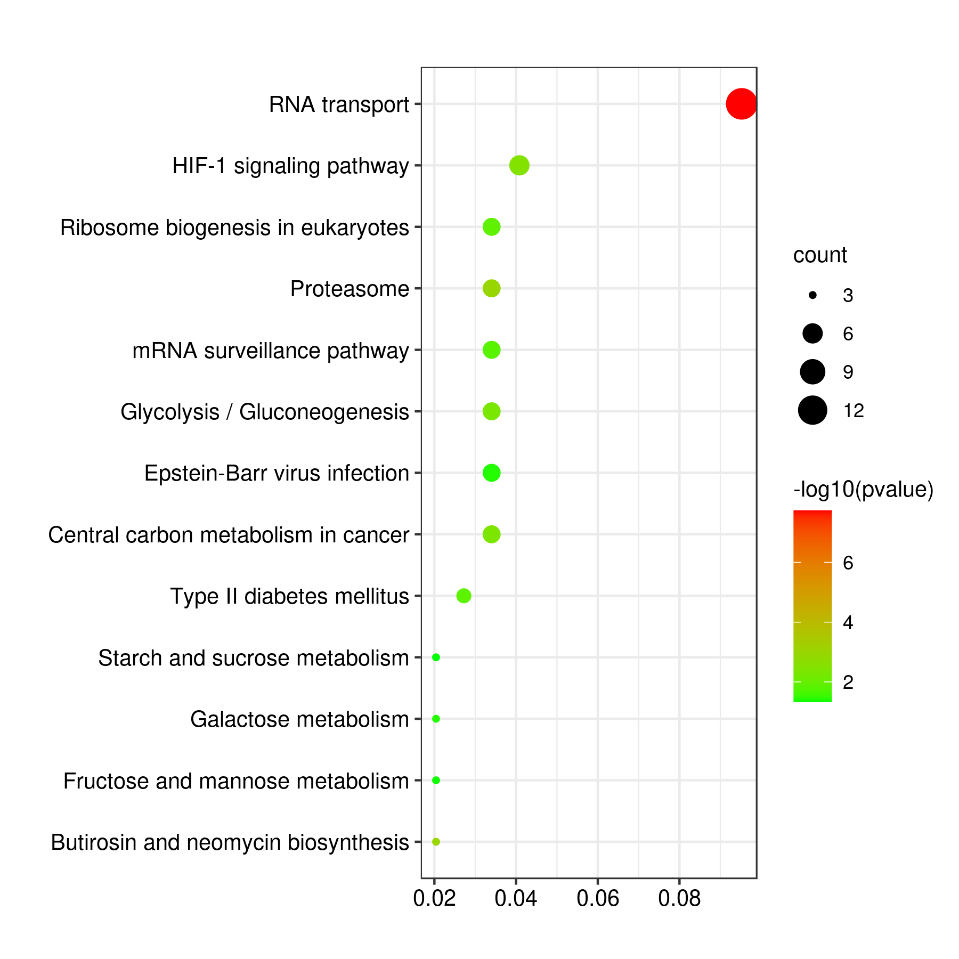

Supplement: Supplementary file 5 — Supplementary Information 5. [file 41598_2022_23598_MOESM5_ESM.docx]
