# Supplementary material for: A pan-cancer analysis of the role of hexokinase II (HK2) in human tumors
Source: Sci Rep. 2022 Nov 5;12:18807. doi: 10.1038/s41598-022-23598-8 (PMC9637150; doi:10.1038/s41598-022-23598-8)
Supplement: Supplementary file 7 — Supplementary Information 7. [file 41598_2022_23598_MOESM7_ESM.docx]

The visual workflow of the steps starting from the dataset


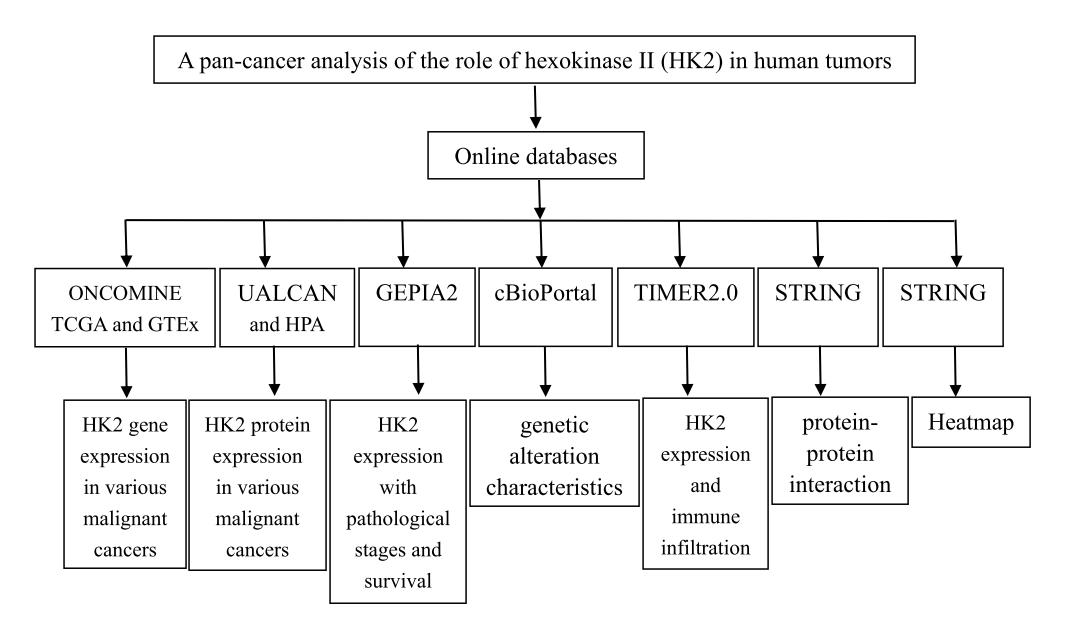


The visual workflow of the steps starting from the dataset. Gene expression of HK2 was from TCGA and GTEx databases. Protein expression was from UALCAN and HPA databases. Data on the relationship between HK2 expression and pathological stages, survival was from GEPIA2 dataset. Gene alteration characteristics was from cBioPortal. Immune infiltration was from TIMER2.0. Protein-protein interaction and heatmap was from STRING.
